# Lewis Acid-Catalyzed Alkylation of Azulene Derivatives with Epoxides and Oxetanes: A Regioselective Approach to Functionalized Azulene Alcohols

**DOI:** 10.1021/acs.orglett.5c02648

**Published:** 2025-08-18

**Authors:** Gonzalo Brotons, Patricia García-Martínez, Olaya Bernardo, Luis A. López

**Affiliations:** Departamento de Química Orgánica e Inorgánica, Instituto Universitario de Química Organometálica “Enrique Moles” and Centro de Innovación en Química Avanzada (ORFEO-CINQA), 16763 Universidad de Oviedo, Julián Clavería 8, 33006 Oviedo, Spain

## Abstract

Upon treatment with a catalytic amount of BF_3_·OEt_2_, the reaction between azulene derivatives and epoxides efficiently affords azulenyl­ethanol derivatives. In most cases, this ring-opening transformation proceeds with high regioselectivity, favoring nucleophilic attack at the more substituted position of the epoxide. Stereochemical studies support an S_N_2-type mechanism for the transformation. Furthermore, a preliminary investigation revealed that appropriately substituted oxetanes can also participate in this ring-opening reaction, delivering the corresponding homologated alcohols under similar catalytic conditions.

## Graphical abstract

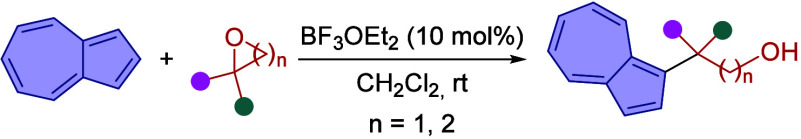

Azulene is an aromatic hydrocarbon distinguished by its unique physicochemical properties, particularly its intense blue color, which has attracted considerable interest in the chemical literature for decades. The distinctive properties of azulene and its functionalized derivatives have led to the development of a broad spectrum of applications in diverse scientific disciplines, such as organic electronics, photonics, medicinal chemistry, and materials science.^1^ As a result, the development of efficient and selective methodologies for the functionalization of azulene remains an active and important area of research in synthetic chemistry.^2^

On the other hand, ring-opening reactions of strained cyclic compounds play a pivotal role in organic synthesis, providing a versatile strategy for constructing structurally diverse and functionally rich molecules. Within this realm, epoxides have emerged as versatile reagents with broad utility in the synthesis of both value-added and commodity chemicals.^3^ While Lewis and Brønsted acid-assisted *intramolecular* Friedel–Crafts reactions of functionalized epoxides bearing a tethered phenyl group have been extensively studied, the corresponding *intermolecular* alkylation has remained largely restricted to a limited number of activated arene derivatives.^4,5^ In 2021, Lebœuf, Moran, and co-workers significantly advanced this field by showing that epoxides can undergo intermolecular Friedel–Crafts arylations with a broad range of arenes when catalyzed by trifluoro­methane­sulfonic acid (TfOH) in hexafluoro­isopropanol (HFIP).^6^

Continuing our interest in the functionalization of azulene derivatives,^7^ we envisioned that these electron-rich systems could undergo Friedel–Crafts-type alkylation with epoxides to afford ring-opening products. To the best of our knowledge, the reaction of azulene with tetracyano­ethylene oxide represents the only previously reported example of azulene’s reactivity toward epoxides. However, this transformation offers limited synthetic utility, as it results in a mixture of products.^8^ Here, we report BF_3_·OEt_2_-catalyzed reactions of azulenes with epoxides, providing a regioselective and efficient route to 2-azulenyl­ethanol derivatives. We also disclose preliminary results using oxetane substrates, which enabled access to homologated functionalized azulene derivatives.^9^

In our exploratory study, the reaction between azulene (**1a**) and racemic styrene oxide (**2a**) was investigated using 10 mol % of various Lewis and Brønsted acids (Table 1; for a detailed screening, see the Supporting Information). Among the tested catalysts, BF_**3**_·OEt_2_ in dichloromethane at room temperature proved most effective, delivering 2-(azulen-1-yl)-2-phenylethan-1-ol (**3aa**) in 85% isolated yield (entry 1). The ring opening of styrene oxide occurred with complete regioselectivity, favoring C–C bond formation at the benzylic position of the aziridine. As for the azulene component, as expected, the reaction took place at the C-1 carbon of the electron-rich five-membered ring.^10,11^ Other Lewis acids, such as InCl_3_ and Cu­(OTf)_2_, gave lower yields of **3aa** (65% and 48%, respectively; entries 2 and 3). Brønsted acids such as acetic acid and trifluoro­acetic acid also promoted the reaction but were less effective than BF_3_·OEt_2_ (entries 4 and 5). Although the combination of TfOH and HFIP has been effective in similar epoxide–arene reactions, it afforded only a low yield in this transformation (entry 6). The use of a BINOL-derived chiral phosphoric acid, (*R*)-(−)-VAPOL hydrogen phosphate, led to the formation of compound **3aa** in a low yield with negligible enantioselectivity (entry 7). Solvent screening revealed that toluene and 1,2-dichloroethane (DCE) were also viable with BF_3_·OEt_2_, yielding **3aa** in 75% and 67% yield, respectively (entries 8 and 9). However, diethyl ether significantly diminished the yield (17%, entry 10). Notably, using acetic acid as both solvent and Brønsted acid catalyst gave **3aa** in a respectable 54% yield without an additional catalyst (entry 11). As expected, no product formation was observed when the reaction was conducted in the absence of a catalyst, highlighting the essential role of the Lewis acid in promoting the transformation (entry 12).

**Table 1:**
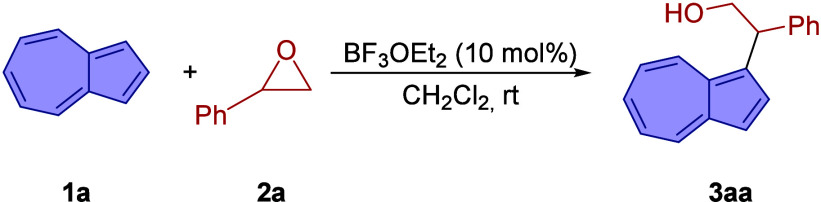
Summary of Optimization of Reaction Conditions^a^

entry	deviation from standard conditions	yield^b^ (%)
1	no changes	85
2	InCl_3_ as the catalyst	65
3	Cu(OTf)_2_ as the catalyst	48
4	CH_3_COOH as the catalyst	76
5	CF_3_COOH as the catalyst	70
6	TfOH in HFIP as the solvent	10
7^c^ ^,^ ^d^	BINOL-derived phosphoric acid as catalyst	25
8	toluene as the solvent	75
9	DCE as the solvent	67
10	Et_2_O as the solvent	17
11	CH_3_COOH as the solvent without BF_3_OEt_2_	54
12^e^	no catalyst	–
13	1 mmol scale	83

a Reaction conditions: azulene (**1a**, 0.6 mmol), styrene oxide (**2a**, 0.2 mmol), CH_2_Cl_2_ (1 mL), 25 °C, 1 h.

b Yield of isolated product after chromatographic purification (silica gel; hexane/ethyl acetate 3:1).

c (*R*)-(−)-VAPOL hydrogen phosphate.

d **3aa** isolated as a racemic mixture.

e Reaction time: 75 h.

To demonstrate the practicality and scalability of the method, the model reaction was successfully performed on a 1 mmol scale, providing the ring-opening product **3aa** without any significant erosion of the yield (entry 13).

With the optimal conditions established, we next explored the substrate scope of this Lewis acid-catalyzed alkylation of azulene (Table 2). We began by examining the reactivity of azulene (**1a**, R^1^ = R^2^ = R^3^ = H) with a range of electronically diverse aryl-substituted epoxides (**2**). *Para*-substituted aryl epoxides bearing electron-donating groups, such as methyl and acyloxy, proved to be excellent substrates, affording the corresponding ring-opening products **3ab** and **3ac** in 78% and 72% yield, respectively, with complete regioselectivity. Similarly, 2-(4-halophenyl) epoxides were well tolerated, delivering the corresponding functionalized azulene derivatives **3ad** (Ar = 4-FC_6_H_4_, 51%) and **3ae** (Ar = 4-ClC_6_H_4_, 57%). Epoxides with *meta*-substituted aryl groups also performed well under the reaction conditions, as demonstrated by the formation of azulene derivatives **3af** (Ar = 3-MeC_6_H_4_, 58%) and **3ag** (Ar = 3-O_2_NC_6_H_4_, 49%). In contrast, an *ortho*-methyl-substituted aryl epoxide exhibited markedly reduced reactivity, delivering only trace amounts of the desired product **3ah**, presumably due to steric hindrance imposed by the *ortho* substituent.

**Table 2:**
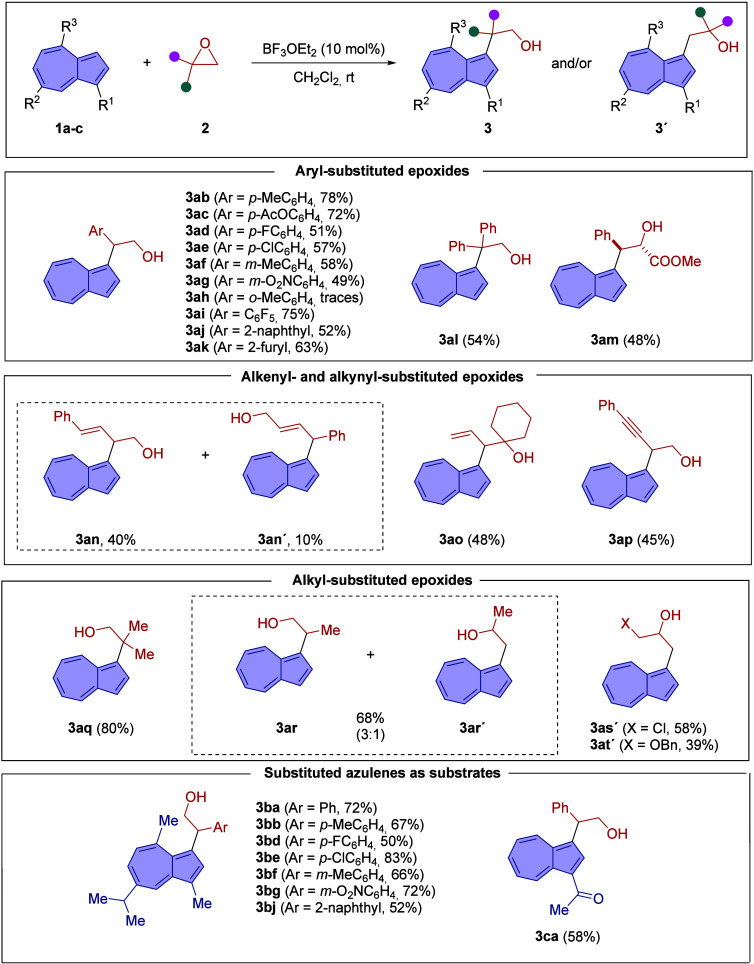
Scope of Reaction of Azulene Derivatives **1a–c** with Epoxides **2** ^a^ ^,^ ^b^

a Reaction conditions: **1** (0.6 mmol), **2** (0.2 mmol), BF_3_OEt_2_ (10 mol %), CH_2_Cl_2_ (1 mL), rt.

b Yields of isolated products are given.

Furthermore, the method was applicable to epoxides featuring highly electron-deficient aryl groups; for example, (pentafluoro­phenyl)­ethylene oxide reacted efficiently under the optimized conditions to provide azulene **3ai** in 75% yield.

Interestingly, 2-(naphthalen-2-yl)­oxirane and 2-(oxiran-2-yl)­furan both underwent smooth ring-opening reactions, furnishing the corresponding azulene derivatives **3aj** and **3ak** in 52% and 65% yield, respectively.

Both 1,1- and 1,2-disubstituted epoxides proved to be suitable substrates for this ring-opening transformation. For example, treatment of 2,2-diphenyl­oxirane with azulene under the optimized conditions afforded the functionalized azulene **3al** in 54% yield as a single regioisomer. Similarly, the use of methyl *trans*-3-phenyl­oxirane-2-carboxylate led to the regio- and stereoselective formation of azulene **3am** also in moderate yield.

The methodology was further extended to vinyl-substituted epoxides. Reaction of azulene with (*E*)-2-styryloxirane resulted in the formation of **3an** and **3an′** in moderate yield as a separable 4:1 mixture of regioisomers. In contrast, the spirocyclic epoxide 2-vinyl-1-oxaspiro[2.5]­octane underwent ring opening with complete regioselectivity, affording azulene derivative **3ao** in moderate yield. Under the developed conditions, an alkynyl-substituted epoxide was also a competent substrate, as demonstrated by the formation of compound **3ap** in 45% yield.

We then assessed the reactivity of alkyl-substituted oxiranes as reaction partners. Isobutylene oxide reacted with azulene under the optimized conditions to afford the functionalized product **3aq** in 80% yield with complete regioselectivity. In contrast, the reaction of propylene oxide with azulene (**1a**) also proceeded efficiently but resulted in a 3:1 inseparable mixture of regioisomers **3ar** and **3ar′**, isolated in 68% yield, indicating partial loss of regioselectivity in this case

To further demonstrate the synthetic utility of this Lewis acid-catalyzed ring-opening transformation, we examined the reactivity of azulene (**1a**) with epichloro­hydrin. Gratifyingly, the reaction proceeded with complete regioselectivity, affording the densely functionalized azulene derivative **3as′** in 58% isolated yield. Interestingly, in this case the reaction took place with complete inversion of the regioselectivity with exclusive nucleophilic attack of azulene to the less-substituted position of the epoxide.^12^ A similar regiochemical outcome was noted with a protected glycidol derivative, namely, benzyl glycidyl ether; however, in this case, the corresponding azulene derivative **3at′** was obtained in modest yield.

We also explored the scope of the reaction using guaiazulene (**1b**, R^1^ = R^3^ = Me; R^2^ = iPr) as the nucleophilic partner. Reactions with various aryl-substituted epoxides proceeded smoothly, delivering the corresponding 2-diaryl-substituted ethanol derivatives featuring an azulenyl group in moderate to good yields (50–83%) while maintaining complete regioselectivity throughout.

Interestingly, 1-acetylazulene (**1c**, R^1^ = COCH_3_; R^2^ = R^3^ = H) proved to be a suitable substrate for the transformation, affording the highly functionalized azulene derivative **3ca** in 58% yield with complete regioselectivity upon reaction with styrene oxide. This result demonstrates that the reaction is compatible with more elaborate azulene derivatives.

To determine whether the epoxide ring opening proceeds via an S_N_1 or S_N_2 mechanism under the developed conditions, we examined the reactivity of azulene (**1a**) with enantioenriched (*R*)- and (*S*)-styrene oxides (Scheme 1). These transformations provided the corresponding optically active products (*R*)- and (*S*)-**3aa**, respectively. The formation of these enantioenriched products strongly supports the stereospecific nature of the transformation and is consistent with a mechanism involving initial coordination of the epoxide oxygen to the Lewis acid, generating a partial positive charge at the benzylic carbon, followed by nucleophilic attack by azulene through a concerted S_N_2-type pathway^13^ rather than via a discrete carbocationic intermediate. Within this mechanistic framework, the formation of compound **3an′** as a minor product in the reaction of azulene with (*E*)-2-styryl­oxirane can be rationalized by a competitive S_N_2′ pathway.

**Scheme 1:**
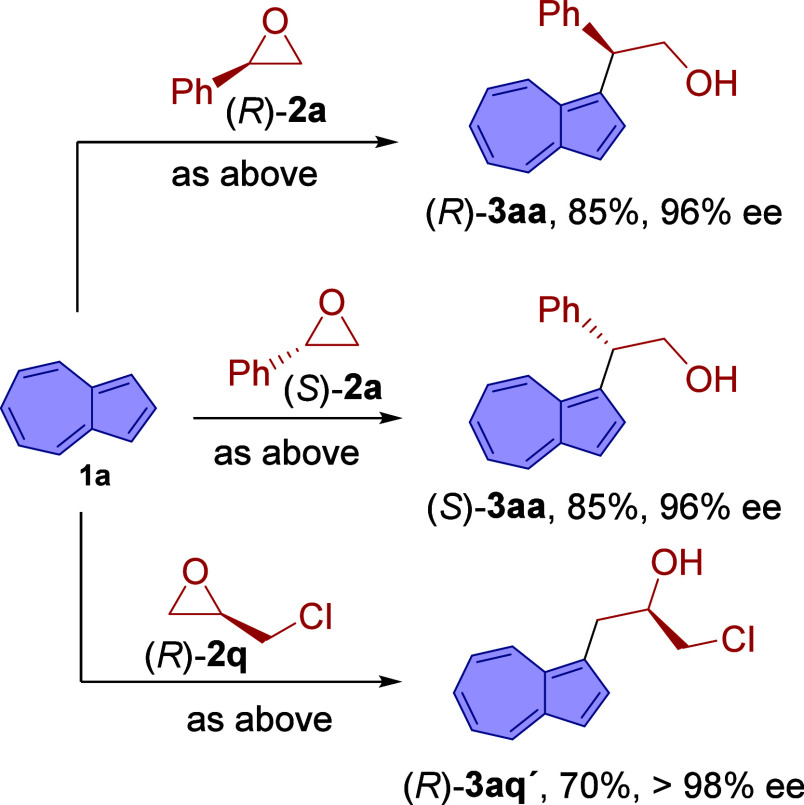
Reaction of Azulene (**1a**) with Optically Active Epoxides

Although less informative mechanistically, since the stereogenic center is not directly involved in the bond-forming event, the reaction of enantioenriched (*R*)-epichloro­hydrin furnished (*R*)-**3as′**, further showcasing the utility of this transformation for the efficient synthesis of enantioenriched azulene-based architectures.

Next, we investigated the suitability of oxetanes as substrates for this ring-opening reaction (Scheme 2). Under the developed conditions, the parent oxetane proved to be unreactive. However, we were pleased to find that the reaction of azulene (**1a**) with 2-phenyl­oxetane (**4a**) afforded the homologated azulene derivative **5aa** in 76% yield. This ring opening occurred with complete regioselectivity, with nucleophilic attack taking place at the more substituted benzylic position of the oxetane. This outcome is consistent with the activation of the oxetane by Lewis acid coordination, leading to preferential ring cleavage at the more stabilized carbocation-like center, analogous to the behavior observed in the corresponding epoxide transformations. The reaction of guaiazulene (**1b**) proceeded with comparable efficiency, affording the corresponding homologated azulene derivative **5ba** in good yield.

**Scheme 2:**
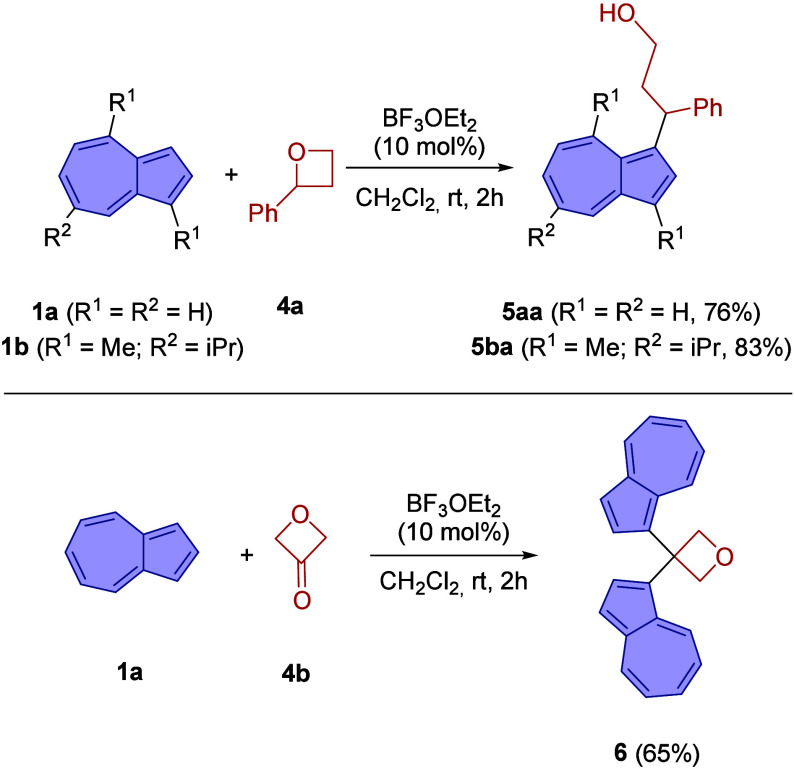
Reaction of Azulenes **1a** and **1b** with Oxetane Derivatives **4a** and **4b**

Under otherwise identical conditions, the reaction of oxetan-3-one (**4b**) with azulene (**1a**) did not afford any ring-opening product. Instead, 3,3-di­(azulen-1-yl)­oxetane (**6**) was isolated in 65% yield.^14^

In summary, we have developed a convenient and broadly applicable protocol for the synthesis of functionalized azulene derivatives via the regioselective ring opening of epoxides. In most cases, the transformation proceeded with complete regioselectivity. Moreover, the use of enantioenriched epoxides enabled access to chiral azulene derivatives through a stereospecific, concerted S_N_2-type mechanism A preliminary investigation also demonstrated the viability of suitably substituted oxetanes as alternative electrophilic partners in this transformation. Further studies aimed at exploring the applications and reactivity of the azulene derivatives accessible through this methodology are currently ongoing in our laboratory.

## Supplementary Material



## Data Availability

The data underlying this study are available in the published article and its Supporting Information.

## Notes

Experimental procedures and characterization data for all new compounds (PDF)

## †

Department of Chemistry and Department of Biochemistry and Molecular Biology, University of British Columbia, Vancouver, BC V6T 1Z1, Canada
