# Supplementary material for: Lewis Acid-Catalyzed Alkylation of Azulene Derivatives with Epoxides and Oxetanes: A Regioselective Approach to Functionalized Azulene Alcohols
Source: Org Lett. 2025 Aug 18;27(34):9412–6. doi: 10.1021/acs.orglett.5c02648 (PMC12400402; doi:10.1021/acs.orglett.5c02648)

Supporting Information

Lewis Acid-Catalyzed Alkylation of Azulene Derivatives with Epoxides and Oxetanes: A Regioselective Approach to Functionalized Azulene Alcohols

Gonzalo Brotons,[a] Patricia García-Martínez,[a] Olaya Bernardo,[a] Luis A. López[a]*

[a] Departamento de Química Orgánica e Inorgánica, Instituto Universitario de Química Organometálica "Enrique Moles", and Centro de Innovación en Química Avanzada (ORFEO-CINQA), Universidad de Oviedo, Julián Clavería 8, 33006-Oviedo, Spain

lalg@uniovi.es

Table of Contents

1. General Considerations S-3

2. Synthesis of Functionalized Azulene Derivatives **3**: Optimization S-5

3. General Procedure for the Preparation of Compounds **3** S-6

4. Characterization Data of Functionalized Azulene Derivatives **3** S-7

5. Experimental Procedure for the Synthesis of **3aa** (1.0 mmol Scale) S-25

6. Experimental Procedure for the Synthesis of **3aa'** S-26

7. Reaction of Azulene (**1a**) with (*R*)-(+)-Styrene Oxide S-27

8. Reaction of Azulene (**1a**) with (*S*)-(-)-Styrene Oxide S-28

9. Reaction of Azulene (1a) with (*R*)-(-)-Epichlorohydrin S-29

10. Extension of the Study to 2-Phenyloxetane (**4a**) S-30

11. Synthesis of 3,3-Di(azulen-1-yl)oxetane (**6**) S-32

12. References S-33

13. NMR Spectra for New Compounds S-34

1. General Considerations

All reactions were carried out using oven-dried glassware under an atmosphere of nitrogen (99.99%). 1,2-Dichloromethane (DCM) was distilled from CaH_2 before use. The solvents used in column chromatography were obtained from commercial suppliers and used without further distillation. TLC was performed on aluminum-backed plates coated with silica gel 60 with F254 indicator (Merck), using UV light as a visualizing agent and phosphomolybdic acid in ethanol, potassium permanganate solution or *p*-anisaldehyde in ethanol, and heat as developing agent. Flash chromatography was performed on silica gel (40-60 um). ^{1}H NMR (300, 400 MHz) and ^{13}C NMR (75.5, 100 MHz) spectra were measured in $CDCl_3$ at room temperature on a Bruker DPX-300, Bruker AV-300 MHz and Bruker AV-400 instruments, with $CDCl_3$ (δ = 7.26, ^{1}H NMR; δ = 77.16, ^{13}C NMR) as internal standard.

High-resolution mass spectra (HRMS) were determined by Universidad de Oviedo with a Bruker Impact II, Q-TOF mass Spectrometer and GC-Q-TOF Agilent 7250 with electronic ionization at 70 eV.

Enantiomer ratios were determined by chiral HPLC analyses with a Vis-UV photodiode Array 2996 or 996 as detector, and compared with the authentic racemic products.

The starting materials used in this study are displayed in Figure S1. Regarding the azulene component, this study was carried out using commercially available azulene (**1a**) and guaiazulene (**1b**). 1-Acetylazulene (**1c**) was prepared according to a literature procedure.[1] Styrene oxide (**2a**), (*R*)-**2a** (97% ee), and (*S*)-**2a** (98% ee) were purchased from commercial suppliers and used as received without further purification. Epichlorohydrin (**2s**) and enantiomerically enriched (*R*)-**2s** (>98%ee) were commercially available and obtained from commercial suppliers without further purification. All other epoxides used in this study were synthesized according to literature procedures.[2] 2-Phenyloxetane (**4a**) was synthesized from styrene oxide following a literature procedure.[3] Oxetane-3-one (**4b**) was commercially available and used without further purification. All other reagents used in this work were of the best commercial grade available and used without further purification.

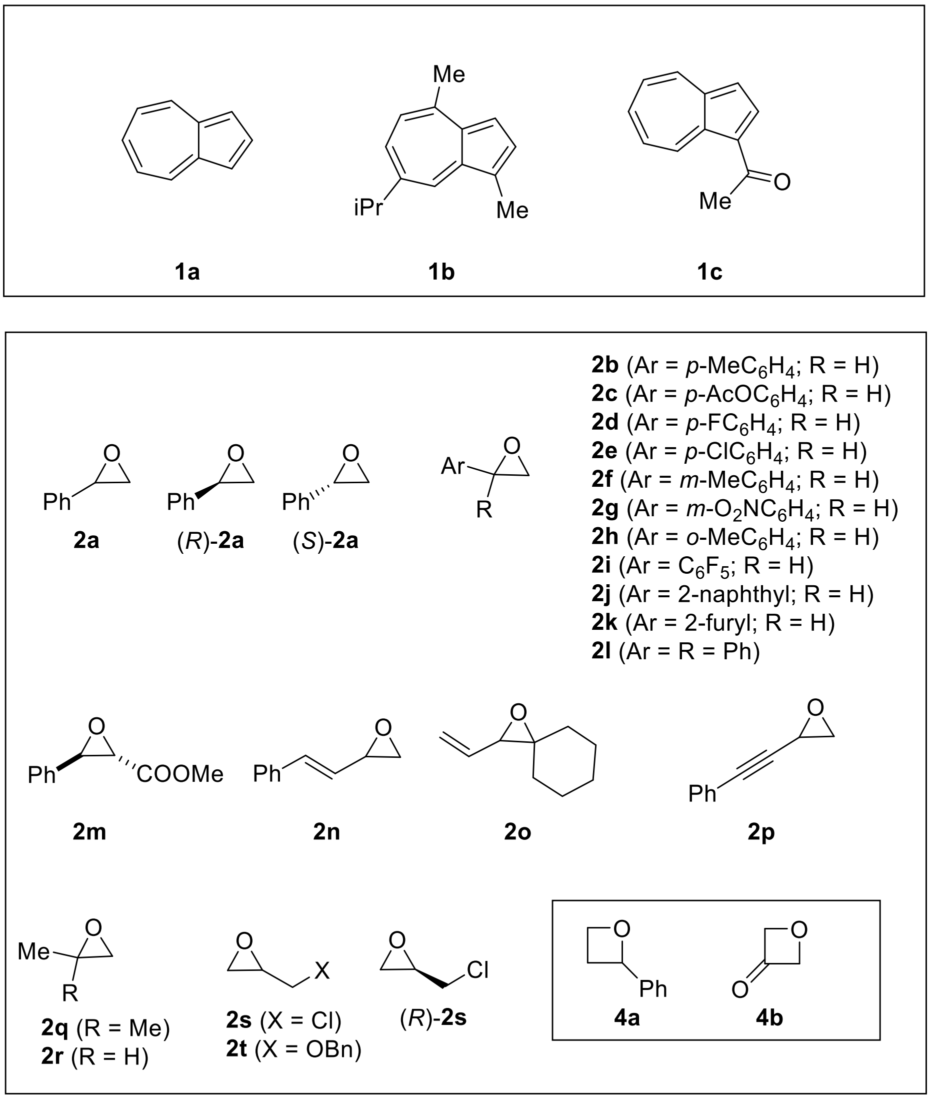

Figure S1. Starting materials used in this work

2. Synthesis of Functionalized Azulene Derivatives 3: Optimization

Table S1: Optimization of Reaction Conditions[a]

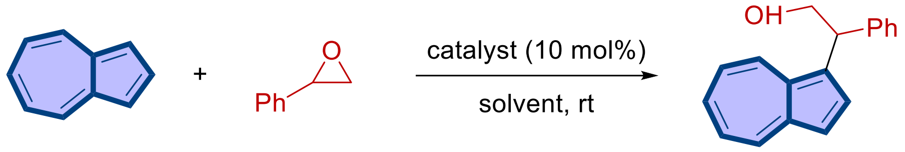

1a (3 equiv) **2a** **3aa**

Entry	Catalyst	Solvent	Yield **3aa** (%)[b]
1	CF_3COOH	CH_2Cl_2	70
2	CH_3COOH	CH_2Cl_2	76
3	$InCl_3$	CH_2Cl_2	65
4	$Cu(OTf)_2$	CH_2Cl_2	48
5	$BF_3{\cdot}OEt_2$	CH_2Cl_2	85
6	TfOH	CH_2Cl_2	64
7[c]	BINOL-derived chiral phosphoric acid	CH_2Cl_2	25[d]
8	TfOH	HFIP	7
9	$BF_3{\cdot}OEt_2$	Toluene	75
10	$BF_3{\cdot}OEt_2$	Et_2O	17
11	$BF_3{\cdot}OEt_2$	DCE	67
12	$BF_3{\cdot}OEt_2$	-	-
13	-	CH_3COOH	54
14[e]	-	CH_2Cl_2	-

[a] Reaction conditions: azulene (**1a**, 0.6 mmol), styrene oxide (**2a**, 0.2 mmol), solvent (1mL), 25 °C. These exploratory experiments were performed on a 0.2 mmol scale. [b] Yield of isolated product after chromatographic purification (silica gel; hexanes/ethyl acetate 3:1). [c] (*R*)-(-)-1,1′-Binaphthyl-2,2′-diyl hydrogenphosphate. [d] Isolated as a racemic mixture. [e] Reaction time: 75 h.

3. General Procedure for the Preparation of Compounds 3/3´

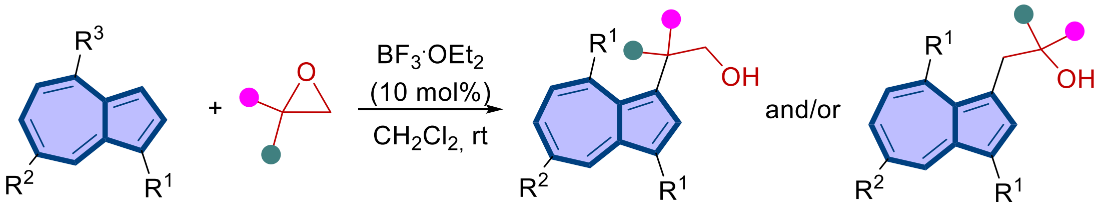

1a-c **2** **3** **3´**

To a solution of azulene (**1a**, 76.9 mg, 0.6 mmol) and the corresponding epoxide **2** (0.2 mmol) in CH_2Cl_2 (1 mL) was added $F_3B{\cdot}OEt_2$ (2.5 μL, 0.02 mmol, 10 mol%) at room temperature. The reaction mixture was stirred until complete consumption of the starting epoxide, as monitored by TLC. The solvent was then removed under reduced pressure, and the crude residue was purified by column chromatography (silica gel, hexanes/ethyl acetate 5:1) to afford the azulene derivatives **3** and/or **3′** as blue oils.

The same procedure was applied using guaiazulene (**1b**, 119.0 mg, 0.6 mmol) and 1-acetylazulene (**1c**, 102.1 mg, 0.6 mmol) in place of azulene (**1a**).

4. Characterization Data of Functionalized Azulene Derivatives 3

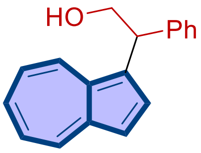

3aa

2-(Azulen-1-yl)-2-phenylethan-1-ol (3aa): The General Procedure was followed using azulene **1a** (76.9 mg, 0.6 mmol) and and styrene oxide (**2a**, 24.0 mg, 0.20 mmol). After 1 h, purification by flash chromatography (silica gel, hexanes/ethyl acetate 5:1) afforded compound **3aa** (42.2 mg, 85% yield) as a blue oil.

^{1}H NMR (300 MHz, $CDCl_3$): δ = 8.36 (d, *J* = 9.7 Hz, 1H), 8.31 (d, *J* = 9.2 Hz, 1H), 7.95 (d, *J* = 3.9 Hz, 1H), 7.57 (t, *J* = 9.8 Hz, 1H), 7.42 (d, *J* = 4.0 Hz, 1H), 7.31-7.06 (m, 7H), 4.90 (t, *J* = 7.2 Hz, 1H), 4.30 (d, *J* = 7.2 Hz, 2H), 1.68 (broad s, 1H) ppm.

^{13}C NMR (75 MHz, $CDCl_3$): δ = 142.5, 140.9, 137.9, 137.0, 136.7, 135.3, 133.7, 129.0, 128.7, 128.3, 126.7, 122.9, 122.5, 117.3, 67.2, 46.6 ppm.

HRMS (EI) m/z: $[M + Na]^+$ Calcd for $C_{18}H_{16}NaO$: 271.1093; Found: 271.1093.

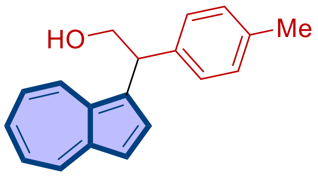

3ab

2-(Azulen-1-yl)-2-(*p*-tolyl)ethan-1-ol (3ab). The General Procedure was followed using azulene **1a** (76.9 mg, 0.6 mmol) and and 2-(*p*-tolyl)oxirane (**2b**, 27.0 mg, 0.20 mmol). After 1 h, purification by flash chromatography (silica gel, hexanes/ethyl acetate 5:1) afforded compound **3ab** (40.9 mg, 78% yield) as a blue oil.

^{1}H NMR (300 MHz, $CDCl_3$): δ = 8.49 (d, *J* = 9.8 Hz, 1H), 8.44 (d, *J* = 9.6 Hz, 1H), 8.09 (d, *J* = 3.9 Hz, 1H), 7.71 (t, *J* = 9.8 Hz, 1H), 7.55 (d, *J* = 3.9 Hz, 1H), 7.37-

7.19 (m, 6H), 5.00 (t, *J* = 7.3 Hz, 1H), 4.42 (d, *J* = 7.3 Hz, 2H), 2.45 (s, 3H) 1.92 (br s, 1H) ppm.

^{13}C NMR (75 MHz, $CDCl_3$): δ = 140.9, 139.3, 137.8, 136.9, 136.6, 136.2, 135.2, 133.7, 129.4, 129.3, 128.0, 122.8, 122.3, 117.2, 67.2, 46.1, 21.0 ppm.

HRMS (EI) m/z: $[M + H]^+$ Calcd for $C_{19}H_{19}O$: 263.1430 ; Found: 263.1430.

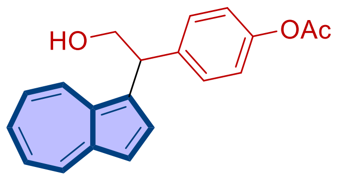

3ac

4-[1-(azulen-1-yl)-2-hydroxyethyl]phenyl acetate (3ac). The General Procedure was followed using azulene (**1a**; 76.9 mg; 0.6 mmol) and 4-(oxiran-2-yl)phenyl acetate (**2c**, 35.6 mg, 0.20 mmol). Purification by flash chromatography (silica gel, hexanes/ethyl acetate 5:1) afforded product **3ac** (44.1 mg, 72% yield).

^{1}H NMR (300 MHz, $CDCl_3$): δ = 8.31 (d, *J* = 9.7 Hz, 2H), 7.93 (d, *J* = 4.0 Hz, 1H), 7.58 (t, *J* = 9.8 Hz, 1H), 7.41 (d, *J* = 3.7 Hz, 1H), 7.32-7.27 (m, 2H), 7.12 (m, 2H), 7.02-6.97 (m, 2H), 4.88 (t, *J* = 7.2 Hz, 1H), 4.27 (d, *J* = 7.3 Hz, 2H), 2.27 (s, 3H), 1.26 (br s, 1H) ppm.

^{13}C NMR (75 MHz, $CDCl_3$): δ = 169.7, 149.3, 141.0, 140.1, 138.0, 137.1, 136.7, 135.3, 133.7, 129.2, 128.7, 128.1, 123.1, 122.5, 121.7, 117.3, 67.2, 46.0, 21.3 ppm.

HRMS (GC-TOF) m/z: $[M]^+$ Calcd for $C_{20}H_{18}O_3$: 306.1256; Found: 306.1256.

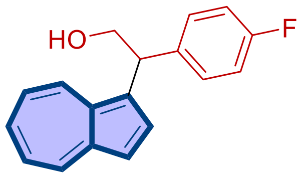

3ad

2-(Azulen-1-yl)-2-(4-fluorophenyl)ethan-1-ol (3ad). The General Procedure was followed using azulene (**1a**, 76.9 mg; 0.6 mmol) and 2-(4-fluorophenyl)oxirane (**2d**, 27.6 mg, 0.2 mmol). Purification by flash chromatography (silica gel, hexanes/ethyl acetate 5:1) afforded product **3ad** (27.2 mg, 51% yield).

^{1}H NMR (300 MHz, $CDCl_3$): δ = 8.33 (d, *J* = 2.1 Hz, 1H), 8.30 (d, *J* = 3.1 Hz, 1H), 7.91 (d, *J* = 3.9 Hz, 1H), 7.58 (t, *J* = 10.1 Hz, 1H), 7.41 (d, *J* = 3.9 Hz, 1H), 7.28-7.23 (m, 2H), 7.13 (dt, *J* = 9.9, 5.0 Hz, 2H), 7.00-6.95 (m, 2H), 4.87 (t, *J* = 7.2 Hz, 1H), 4.27 (dd, *J* = 7.2, 1.8 Hz, 2H), 1.64 (br s, 1H) ppm.

^{13}C NMR (75 MHz, $CDCl_3$): δ = 161.7 (d, *J* = 245.0 Hz), 141.0, 138.3 (d, *J* = 3.0 Hz), 138.1, 137.2, 136.6, 135.2, 133.7, 129.7 (d, *J* = 7.9 Hz), 128.8, 123.10, 122.6, 117.3, 115.5 (d, *J* = 21.2 Hz), 67.2, 45.8 ppm.

^{19}F NMR (282 MHz, $CDCl_3$): δ = -116.5 ppm.

HRMS (GC-TOF) m/z : $[M]^+$ Calcd for $C_{18}H_{15}FO$ 266.1101 ; Found: 266.1107

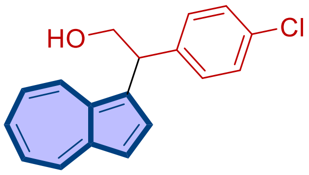

3ae

2-(Azulen-1-yl)-2-(4-chlorophenyl)ethan-1-ol (3ae). The General Procedure was followed using azulene (**1a**, 76.9 mg; 0.6 mmol) and 2-(4-chlorophenyl)oxirane (**2e**, 30.8 mg, 0.2 mmol). Purification by flash chromatography (silica gel, hexanes/ethyl acetate 5:1) afforded product **3ae** (32.2 mg, 57% yield) as a blue oil.

^{1}H NMR (300 MHz, $CDCl_3$): δ = 8.32 (d, *J* = 7.4 Hz, 1H), 8.29 (d, *J* = 6.5 Hz, 1H), 7.90 (d, *J* = 4.0 Hz, 1H), 7.59 (t, *J* = 10.1 Hz, 1H), 7.41 (d, *J* = 3.9 Hz, 1H), 7.28-7.23 (m, 2H), 7.13 (dt, *J* = 9.8, 5.7 Hz, 2H), 7.00-6.95 (m, 2H), 4.87 (t, *J* = 7.2 Hz, 1H), 4.27 (dd, *J* = 7.2, 1.8 Hz, 2H), 1.64 (br s, 1H) ppm.

^{13}C NMR (75 MHz, $CDCl_3$): δ = 141.2, 141.0, 138.1, 137.2, 136.6, 135.2, 133.7, 132.4, 129.6, 128.8, 128.5, 123.1, 122.6, 117.4, 67.0, 46.0 ppm.

HRMS (GC-TOF) m/z: $[M]^+$ Calcd for $C_{18}H_{15}ClO$: 282.0806; Found: 282.0811.

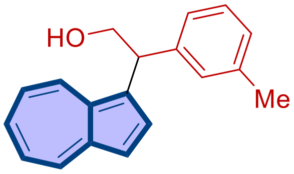

3af

2-(Azulen-1-yl)-2-(*m*-tolyl)ethan-1-ol (3af). The General Procedure was followed using azulene (**1a**, 76.9 mg; 0.6 mmol) and 2-(*m*-tolyl)oxirane (**2f**, 27.0 mg, 0.2 mmol). Purification by flash chromatography (silica gel, hexanes/ethyl acetate 5:1) afforded product **3af** as a blue solid (30.4 mg, 58% yield).

^{1}H NMR (300 MHz, $CDCl_3$): δ = 8.40 (d, *J* = 9.7 Hz, 1H), 8.34 (d, *J* = 9.5 Hz, 1H), 7.99 (d, *J* = 3.9 Hz, 1H), 7.60 (t, *J* = 9.9 Hz, 1H), 7.45 (d, *J* = 4.0 Hz, 1H), 7.22-7.07 (m, 6H), 7.01 (d, *J* = 7.5 Hz, 1H), 4.90 (t, *J* = 7.3 Hz, 1H), 4.32 (d, *J* = 7.3 Hz, 1H), 2.35 (s, 3H), 1.71 (br s, 1H) ppm.

^{13}C NMR (75 MHz, $CDCl_3$): δ = 142.4, 140.9, 138.4, 137.9, 137.0, 136.7, 135.4, 133.7, 129.3, 129.0, 128.6, 127.5, 125.3, 122.9, 122.4, 117.3, 67.3, 46.6, 21.6 ppm.

HRMS (EI) m/z: $[M + H]^+$ Calcd for $C_{19}H_{19}O$: 263.1430; Found: 263.1430.

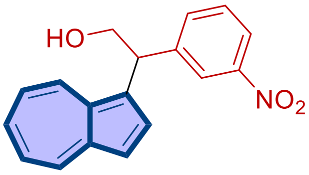

3ag

2-(Azulen-1-yl)-2-(3-nitrophenyl)ethan-1-ol (3ag). The General Procedure was followed using azulene (**1a**; 76.9 mg; 0.6 mmol) and 2-(3-nitrophenyl)oxirane (**2g**; 33.0 mg, 0.2 mmol). Purification by flash chromatography (silica gel, hexanes/ethyl acetate 5:1) afforded product **3ag** as a blue solid (28.7 mg, 49% yield).

^{1}H NMR (300 MHz, $CDCl_3$): δ = 8.37 (d, *J* = 10.6 Hz, 1H); 8.32 (d, *J* = 10.5 Hz, 1H), 7.97 (d, *J* = 3.9 Hz, 1H), 7.44 (d, *J* = 3.9 Hz, 1H), 7.39-7.08 (m, 7H), 4.92 (t, *J* = 7.3 Hz, 1H), 4.33 (d, *J* = 7.3 Hz, 2H), 1.66 (s, 1H) ppm.

^{13}C NMR (75 MHz, $CDCl_3$): δ = 148.5, 145.1, 141.0, 138.3, 137.4, 136.6, 135.2, 134.6, 133.5, 129.5, 127.4, 123.4, 123.1, 122.7, 121.7, 117.6, 66.7, 46.1 ppm.

HRMS (EI) m/z: $[M + Na]^+$ Calcd for $C_{18}H_{15}NaNO_3$: 316.0944; Found: 316.0945.

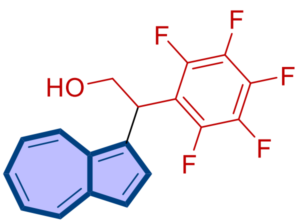

3ai

2-(Azulen-1-yl)-2-(perfluorophenyl)ethan-1-ol (3ai). The General Procedure was followed using azulene (**1a**, 76.9 mg; 0.6 mmol) and 2-(2,3,4,5,6-pentafluorophenyl)oxirane (**2i**, 42.0 mg, 0.2 mmol). Purification by flash chromatography (silica gel, hexanes/ethyl acetate 5:1) afforded product **3ai** as a blue oil (50.7 mg, 75% yield).

^{1}H NMR (300 MHz, $CDCl_3$): δ = 8.42 (d, *J* = 9.8 Hz, 1H), 8.32 (d, *J* = 9.5 Hz, 1H), 7.99 (dt, *J* = 3.9, 1.8 Hz, 1H), 7.64 (t, *J* = 9.9 Hz, 1H), 7.39 (d, *J* = 4.0 Hz, 1H), 7.21 (dt, *J* = 15.4, 9.8 Hz, 2H), 5.29 (t, *J* = 7.9 Hz, 1H), 4.44 (d, *J* = 7.9 Hz, 2H), 1.88 (s, 1H) ppm.

^{13}C NMR (75 MHz, $CDCl_3$): δ = 145.4 (dm, *J* = 245.1 Hz), 141.0, 140.0 (dm *J* = 252 Hz), 138.2, 138.1, 137.8 (dm, *J* = 252 Hz), 137.2, 136.7, 135.4 (t, *J* = 3.4 Hz), 133.0, 125.1, 123.5, 123.0, 117.5, 116.1 (t, *J* = 17,8 Hz), 64.5 (t, *J* = 3.7 Hz), 36.8 ppm.

^{19}F NMR (282 MHz, $CDCl_3$): δ = - 141.9 (dd, *J* = 22.6, 7.7 Hz), - 156.8 (t, *J* = 21.0 Hz), - 161.9 (m) ppm.

HRMS (GC-TOF) m/z: $[M]^+$ Calcd for $C_{18}H_{11}F_5O$: 338.0725; Found: 338.0730.

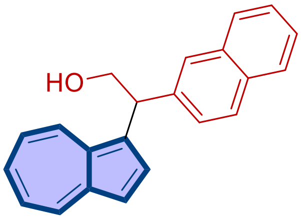

3aj

2-(Azulen-1-yl)-2-(naphthalen-2-yl)ethan-1-ol (3aj). The General Procedure was followed using azulene (**1a**, 76.9 mg; 0.6 mmol) and 2-(naphthalen-2-yl)oxirane (**2j**, 34.0 mg, 0.2 mmol). Purification by flash chromatography (silica gel, hexanes/ethyl acetate 5:1) afforded product **3aj** as a blue oil (31.0 mg, 52% yield).

^{1}H NMR (300 MHz, $CDCl_3$): δ = 8.39 (d, *J* = 9.8 Hz, 1H), 8.32 (d, *J* = 9.5 Hz, 1H), 7.99 (d, *J* = 3.9 Hz, 1H), 7.83-7.72 (m, 4H), 7.57 (t, *J* = 9.9 Hz, 1H), 7.47-7.38 (m, 4H), 7.12 (td, *J* = 9.7, 6.5 Hz, 2H), 5.06 (t, *J* = 7.1 Hz, 1H), 4.56-4.23 (m, 2H), 1.67 (s, 1H).

^{13}C NMR (75 MHz, $CDCl_3$): δ = 141.0, 140.0, 138.0, 137.1, 136.7, 135.5, 133.8, 133.6, 132.5, 129.0, 128.5, 127.9, 127.7, 126.8, 126.6, 126.2, 125.7, 123.0, 122.5, 117.3, 67.1, 46.8 ppm.

HRMS (EI) m/z: $[M + H]^+$ Calcd for $C_{22}H_{19}O$: 299.1430 ; Found: 299.1424.

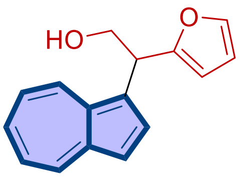

3ak

2-(Azulen-1-yl)-2-(furan-2-yl)ethan-1-ol (3ak). The General Procedure was followed using azulene (**1a**, 76.9 mg; 0.6 mmol) and 2-(oxiran-2-yl)furan (**2k**, 22.0 mg, 0.2 mmol). Purification by flash chromatography (silica gel, hexanes/ethyl acetate 5:1) afforded product **3ak** as a blue oil (30.0 mg, 63% yield).

^{1}H NMR (300 MHz, $CDCl_3$): δ = 8.41 (d, *J* = 9.8 Hz, 1H), 8.32 (d, *J* = 9.0 Hz, 1H), 7.90 (d, *J* = 3.9 Hz, 1H), 7.61 (t, *J* = 9.9 Hz, 1H), 7.39 (d, J = 3.9 Hz, 1H), 7.37

(dd, J = 1.9 and 0.9 Hz, 1H), 7.18 (t, *J* = 9.8 Hz, 1H), 7.16 (t, *J* = 9.5 Hz, 1H), 6.31 (dd, J = 3.2 and 1.8 Hz, 1H), 6.09 (dd, J = 3.2 and 0.9 Hz, 1H), 4.95 (t, *J* = 7.0 Hz, 1H), 4.45-4.23 (m, 1H), 4.22-4.08 (m, 1H), 1.72 (br s, 1H) ppm.

^{13}C NMR (75 MHz, $CDCl_3$): δ = 155.8, 141.6, 141.1, 137.9, 137.0, 136.3, 136.0, 133.3, 126.5, 123.0, 122.5, 117.3, 110.2, 106.4, 66.0, 40.7 ppm.

HRMS (EI) m/z: $[M + H]^+$ Calcd for $C_{16}H_{15}O_2$: 239.1067; Found: 239.1058.

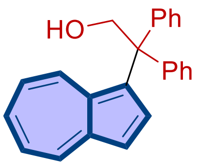

3al

2-(Azulen-1-yl)-2,2-diphenylethan-1-ol (3al). The General Procedure was followed using azulene (**1a**, 76.9 mg; 0.6 mmol) and 2,2-diphenyloxirane (**2l**, 39.2 mg, 0.2 mmol). Purification by flash chromatography (silica gel, hexanes/ethyl acetate 5:1) afforded product **3al** as a blue oil (35.0 mg, 54% yield).

^{1}H NMR (300 MHz, $CDCl_3$): δ = 8.34 (d, *J* = 8.5 Hz, 1H), 7.84 (d, *J* = 9.9 Hz, 1H), 7.76 (d, *J* = 4.0 Hz, 1H), 7.51 (t, *J* = 9.9 Hz, 1H), 7.37-7.21 (m, 11H), 7.14 (t, *J* = 9.6 Hz, 1H), 6.94-6.79 (m, 1H), 4.81 (d, *J* = 3.4 Hz, 2H), 1.53 (s, 1H) ppm.

^{13}C NMR (75 MHz, $CDCl_3$): δ = 145.4, 142.0, 140.2, 137.6, 137.1, 136.6, 135.6, 132.1, 129.1, 128.3, 126.5, 123.2, 122.2, 116.8, 71.0, 57.0 ppm.

HRMS (EI) m/z: $[M + H]^+$ Calcd for $C_{24}H_{21}O$: 325.1587 ; Found: 325.1578.

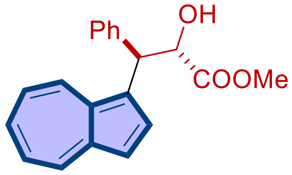

3am

Methyl (2*S,3*R**)-3-(azulen-1-yl)-2-hydroxy-3-phenylpropanoate (3am).** The General Procedure was followed using azulene (**1a**, 76.9 mg; 0.6 mmol) and methyl (2*S**,3*R**)-3-phenyloxirane-2-carboxylate (**2m**, 35.6 mg, 0.2 mmol).

Purification by flash chromatography (silica gel, hexanes/ethyl acetate 5:1) afforded product **3am** as a blue oil (29.4 mg, 48% yield).

^{1}H NMR (300 MHz, $CDCl_3$): δ = 8.31 (s, 1H), 8.30 (d, *J* = 13.8 Hz, 1H), 8.21 (d, *J* = 9.4 Hz, 1H), 7.53 (t, *J* = 9.8 Hz, 1H), 7.40 (d, *J* = 4.0 Hz, 1H), 7.30-7.17 (m, 5H), 7.14-7.01 (m, 2H), 5.15 (d, *J* = 3.8 Hz, 1H), 5.02 (d, *J* = 3.8 Hz, 1H), 3.71 (s, 3H), 2.83 (s, 1H) ppm.

^{13}C NMR (75 MHz, $CDCl_3$): δ = 174.1, 140.6, 139.8, 137.5, 137.3, 136.7, 135.7, 133.1, 129.0, 128.4, 127.0, 122.8, 122.2, 117.1, 74.6, 52.5, 47.7 ppm.

HRMS (EI) m/z: $[M + H]^+$ Calcd for $C_{20}H_{19}O_3$: 307.1329 ; Found: 307.1317.

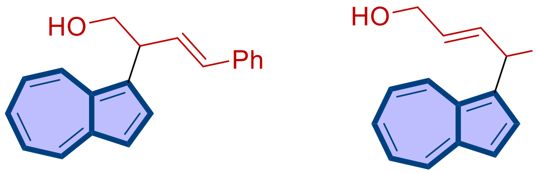

3an **3an'**

(*E*)-2-(Azulen-1-yl)-4-phenylbut-3-en-1-ol (3an) and (*E*)-4-(Azulen-1-yl)-4-phenylbut-2-en-1-ol (3an'). The General Procedure was followed using azulene (**1a**, 76.9 mg; 0.6 mmol) and (*E*)-2-styryloxirane (**2n**, 29.2 mg, 0.2 mmol). Purification by column chromatography (silica gel, hexanes/ethyl acetate: 5:1) afforded a separable mixture of azulene derivatives **3an** (21.6 mg, 40% yield) and **3an'** (5.5 mg, 10%).

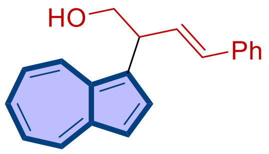

3an

(*E*)-2-(Azulen-1-yl)-4-phenylbut-3-en-1-ol (3an)

^{1}H NMR (300 MHz, $CDCl_3$): δ = 8.39 (d, *J* = 9.0 Hz, 1H), 8.50 (d, *J* = 9.8 Hz, 1H), 7.98 (d, *J* = 3.9 Hz, 1H), 7.67 (t, *J* = 9.9 Hz, 1H), 7.48 (d, *J* = 3.9 Hz, 1H), 7.45-

7.29 (m, 5H), 7.25-7.17 (m, 2H), 6.69-6.50 (m, 2H), 4.52-4.34 (m, 1H), 4.12 (dt, *J* = 7.9, 3.7 Hz, 2H), 1.60 (s, 1H) ppm.

^{13}C NMR (75 MHz, $CDCl_3$) δ = 141.0, 138.0, 137.1, 137.0, 136.4, 135.1, 133.5, 131.5, 130.5, 128.5, 128.2, 127.4, 126.3, 122.9, 122.4, 117.4, 66.8, 44.3 ppm.

HRMS (EI) m/z: $[M + Na]^+$ Calcd for $C_{20}H_{18}NaO$: 297.1250; Found: 297.1250.

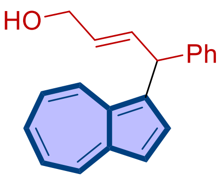

3an'

(E)-4-(Azulen-1-yl)-4-phenylbut-2-en-1-ol (3an')

^{1}H NMR (300 MHz, $CDCl_3$): δ = 8.32 (d, *J* = 9.5 Hz, 1H), 8.31 (d, *J* = 9.8 Hz, 1H), 7.77 (d, *J* = 3.9 Hz, 1H), 7.58 (t, *J* = 9.9 Hz, 1H), 7.38 (d, *J* = 3.9 Hz, 1H), 7.34-7.26 (m, 3H), 7.24-7.06 (m, 4H), 6.37 (ddt, *J* = 15.3, 7.1, 1.5 Hz, 1H), 5.64 (dtd, *J* = 15.3, 5.7, 1.4 Hz, 1H), 5.44 (d, *J* = 7.1 Hz, 1H), 4.20 (d, *J* = 5.7 Hz, 1H) ppm.

^{13}C NMR (75 MHz, $CDCl_3$): δ = 144.2, 141.1, 137.7, 137.0, 135.4, 135.2, 133.7, 131.2, 130.3, 128.5, 128.4, 126.4, 122.8, 122.2, 117.01, 63.7, 46.5 ppm.

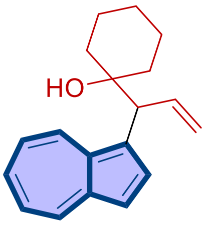

3ao

1-[1-(Azulen-1-yl)allyl]cyclohexan-1-ol (3ao). The General Procedure was followed using azulene (**1a**; 76.9 mg; 0.6 mmol) and 2-vinyl-1-oxaspiro[2.5]octane (**2o**, 27.6, 0.2 mmol). Purification by flash chromatography (silica gel, hexanes/ethyl acetate 5:1) afforded product **3ao** (25.6 mg, 48% yield) as a blue oil.

^{1}H NMR (300 MHz, $CDCl_3$): δ = 8.41 (d, *J* = 9.9 Hz, 1H), 8.29 (d, *J* = 9.4 Hz, 1H), 7.95 (d, *J* = 3.9 Hz, 1H), 7.57 (t, *J* = 9.9 Hz, 1H), 7.40 (d, *J* = 3.9 Hz, 1H), 7.15 (t, *J* = 9.7 Hz, 1H), 7.12 (t, *J* = 9.5 Hz, 1H), 6.51-6.36 (m, 1H), 5.19-5.09 (m, 2H), 4.01 (d, *J* = 9.4 Hz, 1H), 1.83-1.70 (m, 1H), 1.63-1.35 (m, 10H) ppm.

^{13}C NMR (75 MHz, $CDCl_3$) δ = 140.9, 138.5, 137.8, 136.9, 136.67, 136.63, 133.9, 128.8, 122.7, 122.2, 117.5, 116.9, 73.8, 53.8, 35.7, 35.2, 25.9, 22.1, 22.0 ppm.

HRMS (EI) m/z: $[M + Na]^+$ Calcd for $C_{19}H_{22}NaO$: 289.1563 ; Found: 289.1567.

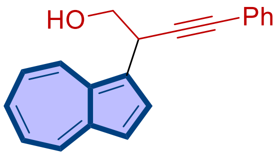

3ap

2-(Azulen-1-yl)-4-phenylbut-3-yn-1-ol (3ap). The General Procedure was followed using azulene (**1a**, 76.9 mg; 0.6 mmol) and 2-(phenylethynyl)oxirane (**2p**, 28.8 mg, 0.2 mmol). Purification by flash chromatography (silica gel, hexanes/ethyl acetate 5:1) afforded product **3ap** as a blue oil (24.5 mg, 45% yield).

^{1}H NMR (300 MHz, $CDCl_3$) δ = 8.50 (d, *J* = 9.8 Hz, 1H), 8.34 (d, *J* = 9.5 Hz, 1H), 8.09 (d, *J* = 3.9 Hz, 1H), 7.63 (t, *J* = 9.9 Hz, 1H), 7.52-7.46 (m, 2H), 7.41 (d, *J* = 3.9 Hz, 1H), 7.34-7.29 (m, 3H), 7.21 (t, *J* = 9.5 Hz, 1H), 7.18 (t, *J* = 9.4 Hz, 1H), 4.77 (t, *J* = 7.0 Hz, 1H), 4.16-3.88 (m, 2H), 1.96 (t, *J* = 6.9 Hz, 1H) ppm.

^{13}C NMR (75 MHz, $CDCl_3$) δ = 141.6, 138.1, 137.3, 136.8, 135.7, 133.5, 131.9, 128.4, 128.2, 125.5, 123.4, 123.3, 122.7, 117.3, 89.4, 83.6, 67.7, 35.1 ppm.

HRMS (EI) m/z: $[M + Na]^+$ Calcd for $C_{20}H_{16}NaO$: 295.1093 ; Found: 295.1092.

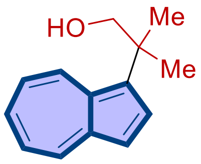

3aq

2-(Azulen-1-yl)-2-methylpropan-1-ol (3aq). The General Procedure was followed using azulene (**1a**, 76.9 mg; 0.6 mmol) and 2,2-dimethyloxirane (**2q**, 14.4 mg, 0.2 mmol). Purification was carried out by column chromatography (silica gel, hexane/ethyl acetate: 5:1), yielding product **3aq** (32.0 mg, 80% yield) as a blue oil.

^{1}H NMR (300 MHz, $CDCl_3$): δ = 8.66 (d, J = 10.0 Hz, 1H), 8.30 (d, J = 9.6 Hz, 1H), 7.91 (d, J = 3.9 Hz, 1H), 7.58 (t, J = 9.8 Hz, 1H), 7.34 (d, J = 3.9 Hz, 1H), 7.16-7.04 (m, 2H), 3.94 (s, 2H), 1.62 (s, 6H), 1.25 (s, 1H) ppm.

^{13}C NMR (75 MHz, $CDCl_3$) δ = 142.1, 137.7, 137.0, 135.5, 134.0, 122.7, 121.6, 117.2, 72.9, 39.8, 27.2 ppm.

HRMS (EI) m/z: $[M + Na]^+$ Calcd for $C_{14}H_{16}NaO$: 223.1093 ; Found: 223.1101.

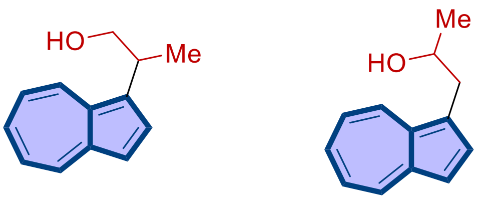

3ar **3ar'**

2-(Azulen-1-yl)propan-1-ol (3ar) and 1-(azulen-1-yl)propan-2-ol (3ar'). The General Procedure was followed using azulene (**1a**; 76.9 mg; 0.6 mmol) and 2-methyloxirane (**2r**, 11.6 mg, 0.2 mmol). Purification was carried out by column chromatography (silica gel, hexane/ethyl acetate: 5:1), yielding 25.3 mg (68% yield) of a 3:1 inseparable mixture of azulene derivatives **3ar** and **3ar'**.

^{1}H NMR (300 MHz, $CDCl_3$, major isomer): δ = 8.39 (d, J = 9.8 Hz, 1H), 8.30 (d, J = 8.5 Hz, 1H), 7.87 (d, J = 3.9 Hz, 1H), 7.58 (t, J = 10.0 Hz, 1H), 7.40 (d, J = 3.9 Hz, 1H), 7.13 (td, J = 9.7, 7.1 Hz, 2H), 3.91-3.77 (m, 2H), 3.70 (q, J = 6.8 Hz, 1H), 1.44 (d, J = 6.8 Hz, 3H) 1.32 (s, 1H) ppm.

^{13}C NMR (75 MHz, $CDCl_3$, major isomer) δ = 141.0, 137.9, 136.9, 134.4, 133.5, 132.1, 122.6, 122.1, 117.5, 117.2, 69.4, 34.6, 18.6 ppm.

HRMS (GC-TOF) m/z: $[M]^+$ Calcd for $C_{13}H_{14}O$: 186.1039; Found: 186.1039.

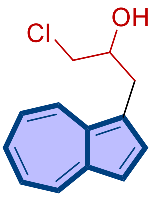

3as'

1-(Azulen-1-yl)-3-chloropropan-2-ol (3as’). The General Procedure was followed using azulene (**1a**; 76.9 mg; 0.6 mmol) and epichlorohydrin (**2s**; 18.4 mg; 0.2 mmol). Purification was carried out by column chromatography (silica gel, hexane/ethyl acetate: 5:1), affording product **3as’** (25.6 mg, 58% yield).

^{1}H NMR (300 MHz, $CDCl_3$): δ = 8.35 (d, *J* = 9.8 Hz, 1H), 8.30 (d, *J* = 8.8 Hz, 1H), 7.84 (d, *J* = 3.8 Hz, 1H), 7.60 (t, *J* = 9.6 Hz, 1H), 7.38 (d, *J* = 3.8 Hz, 1H), 7.22-7.05 (m, 2H), 4.18 (qd, *J* = 6.4, 4.1 Hz, 1H), 3.64-3.48 (m, 2H), 3.39 (d, *J* = 6.6 Hz, 2H), 2.30 (s, 1H) ppm.

^{13}C NMR (75 MHz, $CDCl_3$): δ = 141.0, 137.9, 136.9, 136.85, 133.8, 124.7, 122.9, 122.3, 117.2, 72.4, 49.4, 32.4 ppm.

HRMS (EI) m/z: $[M + H]^+$ Calcd for $C_{13}H_{14}ClO$: 221.0728 ; Found: 221.0727.

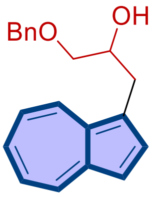

3at'

1-(Azulen-1-yl)-3-(benzyloxy)propan-2-ol (3at’). The General Procedure was followed using azulene (**1a**, 76.9 mg; 0.6 mmol) and benzyl glycidyl ether (**2t**, 32.8 mg, 0.2 mmol). Purification was carried out by column chromatography (silica gel, hexane/ethyl acetate: 5:1), affording product **3at’** (22.8 mg, 39% yield).

^{1}H NMR (300 MHz, $CDCl_3$): δ = 8.33 (d, *J* = 9.7 Hz, 1H), 8.28 (d, *J* = 9.5 Hz, 1H), 7.80 (d, *J* = 3.8 Hz, 1H), 7.57 (t, *J* = 9.9 Hz, 1H), 7.40-7.27 (m, 6H), 7.12 (t, *J* = 9.8 Hz, 1H), 7.11 (t, *J* = 9.5 Hz, 1H), 4.53 (s, 2H), 4.18 (dt, *J* = 10.4, 5.2 Hz, 1H) 3.55-3.39 (m, 2H), 3.36 (dd, *J* = 9.4, 6.6 Hz, 1H), 3.31 (d, *J* = 6.6 Hz, 2H) ppm.

^{13}C NMR (75 MHz, $CDCl_3$): δ = 140.8, 138.0, 137.9, 137.6, 136.5, 133.7, 128.5, 127.8, 125.8, 122.5, 121.9, 117.0, 73.7, 73.4, 71.6, 31.7 ppm.

HRMS (EI) m/z: $[M + H]^+$ Calcd for $C_{20}H_{21}O_2$: 293.1536 ; Found: 293.1535.

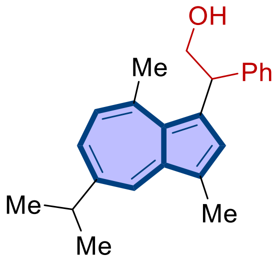

3ba

2-(5-Isopropyl-3,8-dimethylazulen-1-yl)-2-phenylethan-1-ol (3ba). The General Procedure was followed using guaiazulene (**1b**; 118.8 mg; 0.6 mmol) and styrene oxide (**2a**, 24.0 mg, 0.20 mmol). Purification by column chromatography (silica gel; hexane/ethyl acetate: 5:1) afforded product **3ba** (45.8 mg, 72% yield).

^{1}H NMR (300 MHz, $CDCl_3$): δ = 8.24 (d, *J* = 2.2 Hz, 1H), 7.84 (s, 1H), 7.45-7.20 (m, 6H), 6.82 (d, *J* = 10.8 Hz, 1H), 5.44 - 5.31 (m, 1H), 4.45-4.11 (m, 2H), 3.14 (p, *J* = 6.9 Hz, 1H), 3.02 (s, 3H), 2.79 (s, 3H), 1.83 (br s, 1H), 1.45 (d, *J* = 6.9 Hz, 6H) ppm.

^{13}C NMR (75 MHz, $CDCl_3$): δ =145.7, 143.7, 139.7, 137.8, 137.0, 135.2, 134.2, 133.7, 128.7, 128.6, 127.3, 126.3, 125.7, 124.9, 68.1, 48.6, 37.7, 27.9, 24.7, 13.3 ppm.

HRMS (GC-TOF) m/z: $[M]^+$ Calcd for $C_{23}H_{26}O$: 318.1978; Found: 318.1983.

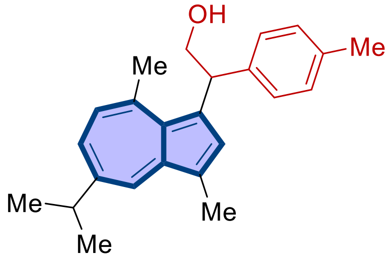

3bb

2-(5-Isopropyl-3,8-dimethylazulen-1-yl)-2-(*p*-tolyl)ethan-1-ol (3bb). The General Procedure was followed using guaiazulene (**1b**; 118.8 mg; 0.6 mmol) and 2-(*p*-tolyl)oxirane (**2b**, 26.8 mg, 0.20 mmol). Purification by column chromatography (silica gel; hexane/ethyl acetate: 5:1) afforded product **3bb** (44.6 mg, 67% yield).

^{1}H NMR (300 MHz, $CDCl_3$): δ = 8.32 (d, *J* = 2.2 Hz, 1H), 7.92 (s, 1H), 7.46 (dd, *J* = 13.0, 2.2 Hz, 1H), 7.38-7.16 (m, 4H), 7.02 (d, *J* = 10.8 Hz, 1H), 5.40 (t, *J* = 7.3 Hz, 1H), 4.46-4.30 (m, 2H), 3.23 (dq, *J* = 13.6, 6.9 Hz, 1H), 3.11 (s, 3H), 2.87 (s, 3H), 2.49 (s, 3H), 1.95 (s, 1H), 1.54 (d, *J* = 6.9 Hz, 6H) ppm.

^{13}C NMR (75 MHz, $CDCl_3$): δ = 145.7, 140.5, 139.6, 137.7, 136.8, 135.8, 135.1, 134.1, 133.6, 129.3, 128.5, 127.2, 125.9, 124.8, 68.1, 48.1, 37.7, 27.8, 24.6, 21.0, 13.2 ppm.

HRMS (EI) m/z: $[M + H]^+$ Calcd for $C_{24}H_{29}O$: 333.2213; Found: 333.2201.

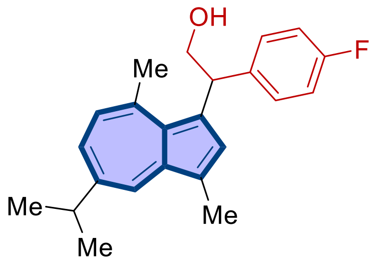

3bd

2-(4-Fluorophenyl)-2-(7-isopropyl-3,5-dimethylazulen-1-yl)ethan-1-ol (3bd). The General Procedure was followed using guaiazulene (1b; 118.8 mg; 0.6 mmol) and 2-(4-fluorophenyl)oxirane (**2d**, 27.6 mg, 0.2 mmol). Purification was carried out by column chromatography (silica gel, hexane:ethyl acetate (5:1)), yielding product **3bd** (33.6 mg, 50% yield).

^{1}H NMR (300 MHz, $CDCl_3$): δ (ppm) = 8.16 (d, *J* = 2.2 Hz, 1H), 7.72 (s, 1H), 7.31(dd, *J* = 10.8, 2.2 Hz, 1H), 7.20-7.09 (m, 2H), 7.03-6.91 (m, 2H), 6.87 (d, *J* = 10.9 Hz, 1H), 5.24 (t, *J* = 7.3 Hz, 1H), 4.33-4.02 (m, 2H), 3.05 (hept, *J* = 6.4 Hz, 1H), 2.91 (s, 3H), 2.70 (s, 3H), 1.73 (s, 1H), 1.37 (d *J* = 6.9 Hz, 6H).

^{13}C NMR (75 MHz, $CDCl_3$): δ (ppm) =161.5 (d, *J* = 245.0 Hz), 145.6, 139.9, 139.5, 137.8, 136.7, 135.3, 134.2, 133.9, 130.2, 130.1, 127.4, 125.2 (d, *J* = 28.8 Hz), 115.6 (d, *J* = 21.2 Hz), 68.1, 47.8, 37.8, 27.8, 24.7, 13.3.

^{19}F NMR (282 MHz, $CDCl_3$): δ = -116.8 ppm.

HRMS (GC-TOF) m/z: $[M]^+$ Calcd for $C_{23}H_{25}FO$: 336.1884; Found: 336.1893.

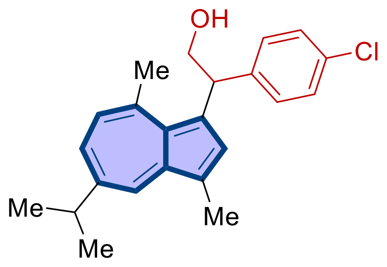

3be

2-(4-Chlorophenyl)-2-(7-isopropyl-3,5-dimethylazulen-1-yl)ethan-1-ol (3be). The General Procedure was followed using guaiazulene (**1b**; 118.8 mg; 0.6 mmol) and 2-(4-chlorophenyl)oxirane (**2e**, 30.8 mg, 0.2 mmol). Purification by flash chromatography (silica gel, hexanes/ethyl acetate 5:1) afforded product **3be** (58.6 mg, 83% yield).

^{1}H NMR (300 MHz, $CDCl_3$): δ (ppm) = 8.19 (s, 1H), 7.73 (s, 1H), 7.34 (d, *J* = 10.8, 1H), 7.28 (d, *J* = 8.5 Hz, 2H), 7.14 (d, *J* = 8.5 Hz, 2H), 6.90 (d, *J* = 10.8 Hz, 1H), 5.27 (t, *J* = 7.2 Hz, 1H), 4.33-4.13(m, 2H),3.09 (m, 1H), 2.93 (s, 3H), 2.72 (s, 3H), 1.75 (s, 1H), 1.40 (d *J* = 6.8 Hz, 6H).

^{13}C NMR (75 MHz, $CDCl_3$): δ =145.6, 142.3, 140.0, 137.9, 136.7, 135.4, 134.2, 133.9, 132.1, 130.1, 128.8, 127.5, 125.4, 67.9, 47.9, 37.8, 27.8, 24.7, 13.3 ppm.

HRMS (GC-TOF) m/z: $[M]^+$ Calcd for $C_{23}H_{25}ClO$: 352.1588; Found: 352.1596.

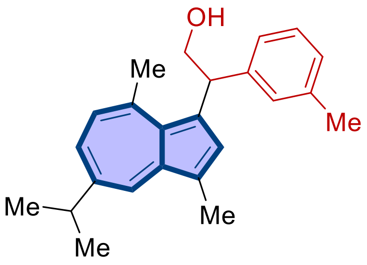

3bf

2-(5-Isopropyl-3,8-dimethylazulen-1-yl)-2-(m-tolyl)ethan-1-ol (3bf). The General Procedure was followed using guaiazulene (**1b**; 118.8 mg; 0.6 mmol) and 2-(m-tolylphenyl)oxirane (**2f**, 26.8 mg, 0.2 mmol). Purification by flash chromatography (silica gel, hexanes/ethyl acetate 5:1) afforded product **3bf** (43.9 mg, 66% yield).

^{1}H NMR (300 MHz, $CDCl_3$): δ = 8.15 (d, *J* = 2.2 Hz, 1H), 7.75 (s, 1H), 7.29 (dd, *J* = 10.7, 2.2 Hz, 1H), 7.18 (dd, *J* = 8.6, 6.9 Hz, 1H), 7.06-6.97 (m, 3H), 6.86 (d, *J* = 10.8 Hz, 1H), 5.23 (t, *J* = 7.3 Hz, 1H), 4.30-4.13 (m, 2H), 3.12-2.99 (m, 1H), 2.94 (s, 3H), 2.70 (s, 3H), 2.30 (s, 3H), 1.74 (s, 1H), 1.36 (d, *J* = 6.9 Hz, 6H) ppm.

^{13}C NMR (75 MHz, $CDCl_3$): δ = 145.7, 143.4, 139.6, 138.2, 137.7, 136.9, 135.1, 134.0, 133.6, 129.4, 128.5, 127.2, 127.1, 125.7, 124.9, 68.1, 48.4, 37.7, 27.9, 24.6, 21.6, 13.3 ppm.

HRMS (EI) m/z: $[M + H]^+$ Calcd for $C_{24}H_{29}O$: 333.2213; Found: 333.2201.

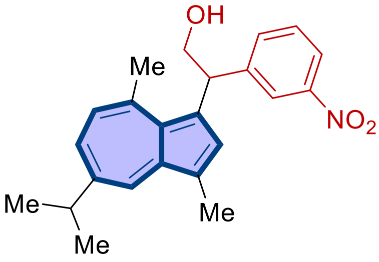

3bg

2-(7-Isopropyl-3,5-dimethylazulen-1-yl)-2-(3-nitrophenyl)ethan-1-ol (3bg). The General Procedure was followed using guaiazulene (**1b**; 118.8 mg; 0.6 mmol) and 2-(3-nitrophenyl)oxirane (**2g**, 33.0 mg, 0.2 mmol). Purification by flash chromatography (silica gel, hexanes/ethyl acetate 5:1) afforded product **3bg** (52.3 mg, 72% yield).

^{1}H NMR (300 MHz, $CDCl_3$): δ = 8.16 (d, *J* = 2.2 Hz, 1H), 8.08 (t, *J* = 2.1 Hz,1H), 8.03 (m, 1H), 7.65 (s, 1H), 7.52 (d, *J* = 7.8 Hz, 1H), 7.42 (t, *J* = 7.9 Hz, 1H), 7.32 (dd, *J* = 10.8, 2.2 Hz, 1H), 6.88 (d, *J* = 10.8 Hz, 1H), 5.37 (t, *J* = 7.2 Hz, 1H), 4.24-4.02 (m, 2H), 3.12-2.98 (m, 1H), 2.91 (s, 3H) 2.67 (s, 3H), 1.80 (s, 1H), 1.35 (d *J* = 7.0 Hz, 6H) ppm.

^{13}C NMR (75 MHz, $CDCl_3$): δ (ppm) = 148.6, 146.2, 145.3, 140.4, 138.1, 136.7, 135.5, 135.1, 134.2, 129.4, 127.7, 125.4, 124.0, 123.5, 121.5, 67.7, 48.1, 37.8, 27.9, 24.7, 13.3 ppm.

HRMS (GC-TOF) m/z: $[M]^+$ Calcd for $C_{23}H_{25}NO_3$: 363.1829; Found: 363.1838.

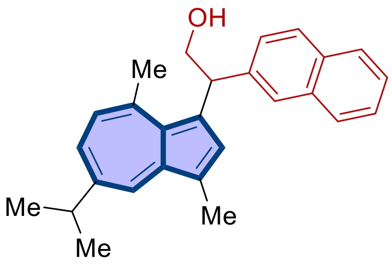

3bj

2-(5-Isopropyl-3,8-dimethylazulen-1-yl)-2-(naphthalen-2-yl)ethan-1-ol (3bj). The General Procedure was followed using guaiazulene (**1b**; 118.8 mg; 0.6 mmol) and 2-(naphthalen-2-yl)oxirane (**2j**, 34.0 mg, 0.2 mmol). Purification by flash chromatography (silica gel, hexanes/ethyl acetate 5:1) afforded product **3bj** (47.2 mg, 64% yield).

^{1}H NMR (300 MHz, $CDCl_3$): δ = 8.19 (d, *J* = 2.2 Hz, 1H), 7.85 – 7.73 (m, 4H), 7.61 (s, 1H), 7.52 – 7.35 (m, 3H), 7.31 (dd, *J* = 10.8, 2.2 Hz, 1H), 6.86 (d, *J* = 10.8 Hz, 1H), 5.44 (t, *J* = 7.2 Hz, 1H), 4.33 (d, *J* = 6.9 Hz, 2H), 3.13 – 3.02 (m, 1H), 2.97 (s, 3H), 2.73 (s, 3H), 1.83 (s, 1H), 1.38 (d, *J* = 6.8 Hz, 6H) ppm.

^{13}C NMR (75 MHz, $CDCl_3$): δ = 145.7, 141.3, 139.8, 137.9, 137.2, 135.2, 134.3, 133.8, 133.7, 132.3, 128.3, 127.9, 127.7, 127.4, 127.3, 127.2, 126.1, 125.7, 125.6, 125.0, 68.0, 48.7, 37.8, 27.9, 24.7, 13.4 ppm.

HRMS (EI) m/z: $[M + H]^+$ Calcd for $C_{27}H_{29}O$: 369.2213; Found: 369.2216.

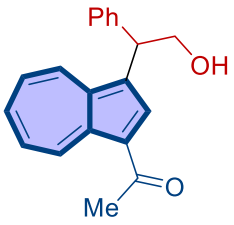

3ca

1-[3-(2-Hydroxy-1-phenylethyl)azulen-1-yl]ethan-1-one (3ca). The General Procedure was followed using 1-acetylazulene (**1c**, 102.1 mg, 0.6 mmol) and styrene oxide (**2a**, 24.0 mg, 0.20 mmol). Purification by flash chromatography (silica gel, hexanes/ethyl acetate 5:1) afforded product **3ca** (33.7 mg, 58% yield) as a blue oil.

^{1}H NMR (300 MHz, $CDCl_3$): δ = 9.65 (d, J = 9.5 Hz, 1H), 8.22 (d, J = 10.0 Hz, 1H), 8.12 (s, 1H), 7.57 (t, J = 9.8 Hz, 1H), 7.35 (t, J = 9.8 Hz, 1H), 7.19 (t, J = 9.8 Hz, 1H), 7.14-6.99 (m, 5H), 4.61 (t, J = 6.9 Hz, 1H), 4.13 (d, J = 6.9 Hz, 2H), 2.51 (s, 3H), 1.66 (br s, 1H) ppm.

^{13}C NMR (75 MHz, $CDCl_3$): δ = 195.5, 141.9, 141.8, 140.9, 139.9, 139.7, 139.0, 135.7, 129.6, 129.0, 128.9, 128.3, 127.1, 127.1, 123.9, 67.0, 46.4, 29.4 ppm.

HRMS (EI) m/z: $[M + H]^+$ Calcd for $C_{20}H_{19}O_2$: 291.1380; Found: 291.1382.

5. Experimental Procedure for the Synthesis of 3aa (1.0 mmol Scale)

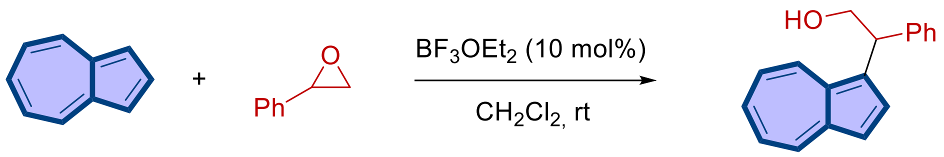

1a **2a** (1.0 mmol) **3aa** (206.1 mg, 83%)

To a solution of azulene **1a** (384.5 mg, 3.0 mmol) and and styrene oxide (**2a**, 120.2 mg, 1.0 mmol) was added $F_3B{\cdot}OEt_2$ (12.3 μL, 0.10 mmol, 10 mol%) at room temperature. The reaction mixture was stirred until complete consumption of the starting epoxide, as monitored by TLC (90 minutes). Then, the solvent was removed under reduced pressure and the resulting mixture was purified by flash chromatography (silica gel, hexanes/ethyl acetate 5:1) to yield compound **3aa** (206.1 mg, 83%) as a blue solid. The spectroscopic data of compound **3aa** match with those reported for the 0.20 mmol scale (see page S-7).

6. Experimental Procedure for the Synthesis of 3aa’

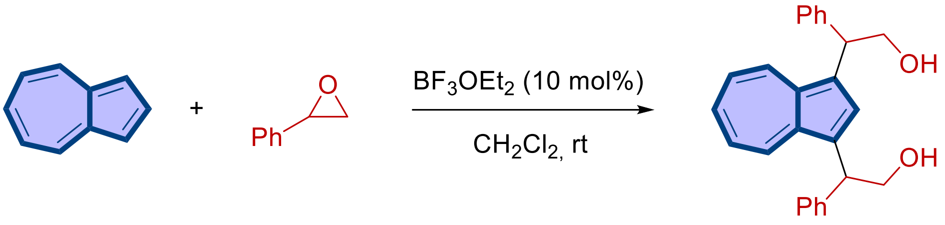

1a **2a** (3 equivalents) **3aa'** (60%, dr = 1:1)

To a solution of azulene (**1a**, 76.9 mg, 0.2 mmol) and styrene oxide (**2a**, 72.1 mg, 0.6 mmol) in CH_2Cl_2 (1 mL) was added $F_3B{\cdot}OEt_2$ (2.5 μL, 0.02 mmol, 10 mol%) at room temperature. The reaction mixture was stirred until complete consumption of the starting epoxide, as monitored by TLC. The solvent was then removed under reduced pressure, and the crude residue was purified by column chromatography (silica gel, hexanes/ethyl acetate 5:1) to afford the compound **3aa’** (44.2 mg, 60%) as a mixture of diastereoisomers. A second careful column chromatography step allowed the isolation of one of the isomers in pure form (14.0 mg, 19%). The following data correspond to this isolated isomer, whose stereochemistry could not be unambiguously assigned.

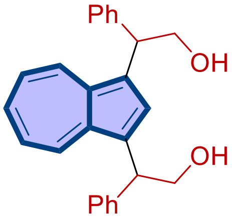

3aa'

^{1}H NMR (300 MHz, $CDCl_3$): δ = 8.28 (d, *J* = 9.6 Hz, 2H), 7.98 (s, 1H), 7.51 (t, *J* = 9.6 Hz, 1H), 7.54-7.20 (m, 10 H), 7.04 (t, *J* = 9.9 Hz, 2H), 4.87 (t, *J* = 7.0 Hz, 2H), 4.29 (d, *J* = 7.0 Hz, 4H), 1.92 (s, 2H) ppm.

^{13}C NMR (75 MHz, $CDCl_3$): δ = 142.5, 138.3, 137.4, 133.8, 133.7, 128.8, 128.3, 128.2, 126.7, 122.4, 67.2, 46.6 ppm.

HRMS (EI) m/z: $[M + Na]^+$ Calcd for $C_{26}H_{24}NaO_2$: 391.1669; Found: 391.1662.

7. Reaction of Azulene (1a) with (*R*)-(+)-Styrene Oxide

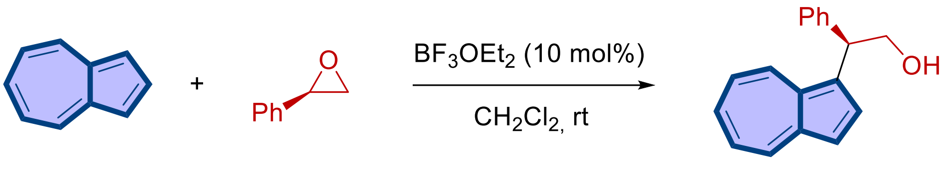

1a (*R*)-**2a** (97% ee) (*R*)-**3aa** (85%, 96% ee)

$BF_3{\cdot}OEt_2$ (2.5 μl, 0.02 mmol, 10 mol%) was added to a solution of azulene (**1a**, 76.9 mg, 0.6 mmol) and and (*R*)-(+)-styrene oxide (**2a**, 24.0 mg, 0.20 mmol, 97% ee) in CH_2Cl_2 (1 mL). The resulting mixture was stirred at room temperature. After 1 h, purification by flash chromatography (silica gel, hexanes/ethyl acetate 5:1) afforded compound (*R*)-**3aa** (42.2 mg, 85% yield, 96% ee) as a blue oil. The spectroscopic data of compound (*R*)-**3aa** match with those reported for the reaction with racemic epoxide (see page S-7). Specific rotation [α] of (*R*)-**3aa** not measurable due to dark color of solution even at very low concentrations. Enantioselectivity was determined by chiral HPLC analysis at room temperature [OD-H, 2-propanol/n-hexane = 10/90, flow rate = 1.0 mL/min, l = 281.7 nm, t_R = 14 min (minor), 21 min (mayor)] (Figure S2).

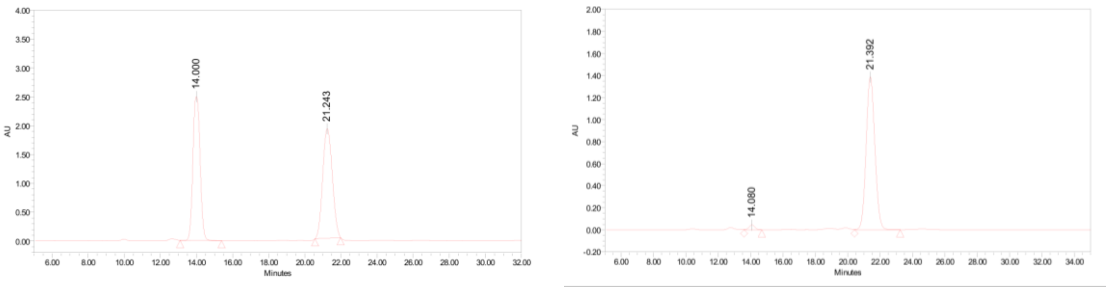

Peak Results

	RT	Area	Height	% Area
1	14.000	68482418	2497029	49.45
2	21.243	70013037	1897373	50.55

Peak Results

	RT	Area	Height	% Area
1	14.080	1045430	43062	1.97
2	21.392	51959033	1386346	98.03

Figure S2. HPLC traces of racemic (left) and enantioenriched (right) (*R*)-**3aa**

8. Reaction of Azulene (1a) with (*S*)-(-)-Styrene Oxide.

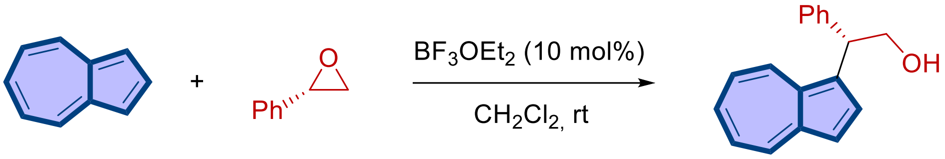

1a (*S*)-**2a** (98% ee) (*S*)-**3aa** (85%, 96% ee)

$BF_3 \cdot OEt_2$ (2.5 μl, 0.02 mmol, 10 mol%) was added to a solution of azulene (**1a**, 76.9 mg, 0.6 mmol) and and (*S*)-(-)-styrene oxide (**2a**, 24.0 mg, 0.20 mmol, 97% ee) in CH_2Cl_2 (1 mL). The resulting mixture was stirred at room temperature. After 1 h, purification by flash chromatography (silica gel, hexanes/ethyl acetate 5:1) afforded compound (*S*)-**3aa** (42.2 mg, 85% yield, 96% ee) as a blue oil. The spectroscopic data of compound **3aa** match with those reported for the reaction with racemic aziridine (see page S-7). Specific rotation [α] of (*S*)-**3aa** not measurable due to dark color of solution even at very low concentrations. Enantioselectivity was determined by chiral HPLC at room temperature [OD-H, 2-propanol/n-hexane = 10/90, flow rate = 1.0 mL/min, l = 281.7 nm, t_R = 13 min (minor), 20 min (mayor)] (Figure S3).

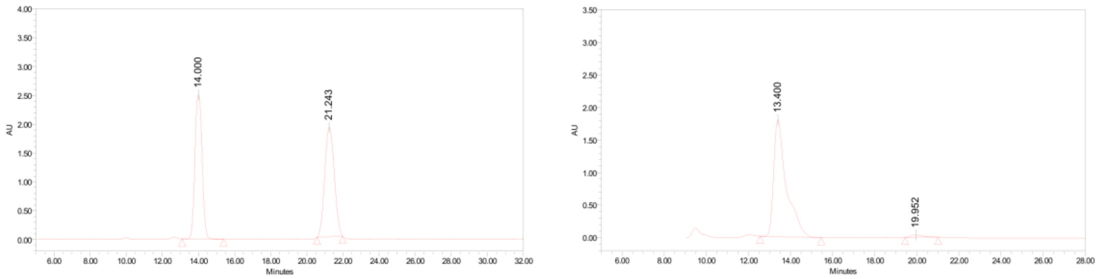

Peak Results

	RT	Area	Height	% Area
1	14.000	68482418	2497029	49.45
2	21.243	70013037	1897373	50.55

Peak Results

	RT	Area	Height	% Area
1	13.400	75239168	1794603	98.01
2	19.952	1530404	37961	1.99

Figure S3. HPLC traces of racemic (left) and enantioenriched (right) (*S*)-**3aa**

9. Reaction of Azulene (1a) with (*R*)-(-)Epichlorohydrin

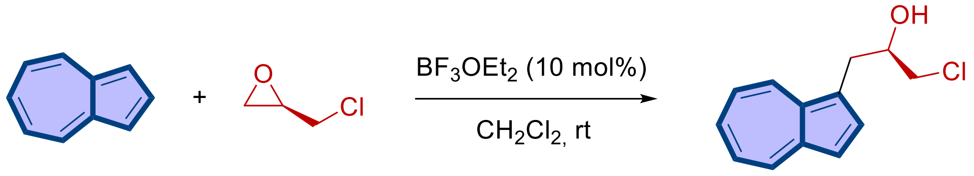

1a (*R*)-**2s** (> 99% ee) (*R*)-**3as'** (70%, > 98% ee)

$BF_3 \cdot OEt_2$ (2.5 μl, 0.02 mmol, 10 mol%) was added to a solution of azulene (**1a**, 76.9 mg, 0.6 mmol) and and (*R*)-(-)-epichlorohydrin (**2s**, 18.5 mg, 0.20 mmol, >99% ee) in CH_2Cl_2 (1 mL). The resulting mixture was stirred at room temperature. After 1 h, purification by flash chromatography (silica gel, hexanes/ethyl acetate 5:1) afforded compound (*R*)-**3as'** (30.9 mg, 70% yield, >98% ee) as a blue oil. The spectroscopic data of compound (*R*)-**3as'** match with those reported for the reaction with racemic aziridine (see page S-18). Specific rotation [α] of (*R*)-**3as'** not measurable due to dark color of solution even at very low concentrations. Enantioselectivity was determined by chiral HPLC analysis at room temperature [OD-H, 2-propanol/n-hexane = 10/90, flow rate = 1.0 mL/min, l = 278.6 nm, t_R = 11 min (mayor), 15 min (minor)] (Figure S4).

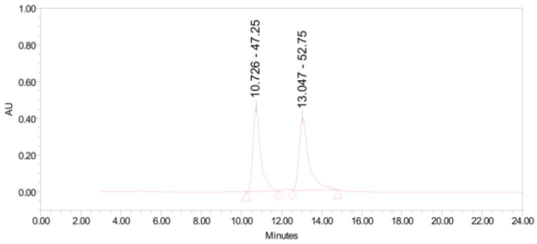

Processed Channel: 2998 PDA 277.1 nm (2998 (190-400)nm)

	Retention Time (min)	Area	% Area	Height
1	10.726	12223187	47.25	460875
2	13.047	13647977	52.75	396917

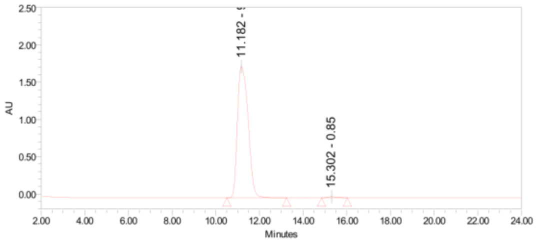

Processed Channel: 2998 PDA 278.6 nm (2998 (190-400)nm)

	Retention Time (min)	Area	% Area	Height
1	11.182	59950366	99.15	1762323
2	15.302	513722	0.85	13023

Figure S4. HPLC traces of racemic (left) and enantioenriched (right) (*R*)-**3as'**

10. Extension of the Study to 2-Phenyloxetane (4a)

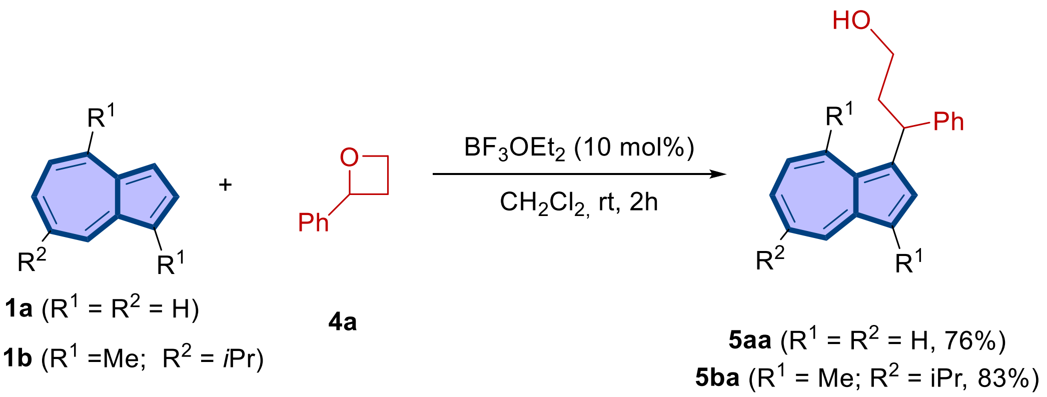

$F_3B{\cdot}OEt_2$ (2.5 μL, 0.02 mmol, 10 mol%) was added at room temperature to a solution of azulene (**1a**, 76.9 mg, 0.6 mmol) and 2-phenyloxetane (**4a**, 26.8 mg, 0.2 mmol) in CH_2Cl_2 (1 mL). The reaction mixture was stirred until complete consumption of the starting epoxide, as monitored by TLC (2 hours). The solvent was then removed under reduced pressure, and the crude residue was purified by column chromatography (silica gel, hexanes/ethyl acetate 5:1) to afford 3-(azulen-1-yl)-3-phenylpropan-1-ol (**5aa**, 39.9 mg, 76%) as a blue oil.

The same procedure was applied using guaiazulene (**1b**, 119.0 mg, 0.6 mmol) in place of azulene (**1a**). In this case, the reaction provided 55.2 mg (83%) of azulene derivarive **5ba**.

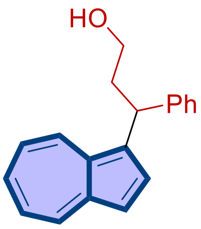

5aa

3-(Azulen-1-yl)-3-phenylpropan-1-ol (5aa)

^{1}H NMR (300 MHz, $CDCl_3$): δ = 8.38 (d, *J* = 9.7 Hz, 1H), 8.26 (d, *J* = 9.3 Hz, 1H), 7.94 (d, *J* = 3.9 Hz, 1H), 7.53 (t, *J* = 9.9 Hz, 1H), 7.38 (d, *J* = 3.9 Hz, 1H), 7.33–7.21 (m, 4H), 7.20–7.02 (m, 3H), 4.82 (t, *J* = 7.8 Hz, 1H), 3.71–3.56 (m, 2H), 2.49 (dt, *J* = 7.9, 6.3 Hz, 2H), 1.55 (s, 1H) ppm.

^{13}**C NMR** (75 MHz, $CDCl_3$) δ = 145.6, 140.8, 137.6, 136.7, 135.6, 135.3, 133.4, 132.5, 128.5, 127.7, 126.0, 122.5, 122.0, 117.1, 61.3, 39.7, 39.7 ppm.

HRMS (EI) m/z: $[M + H]^+$ Calcd for $C_{19}H_{19}O$: 263.1430; Found: 263.1426.

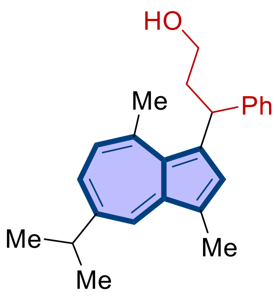

5ba

3-(5-Isopropyl-3,8-dimethylazulen-1-yl)-3-phenylpropan-1-ol (5ba)

^{1}**H NMR** (300 MHz, $CDCl_3$): δ = 8.10 (d, *J* = 2.2 Hz, 1H), 7.72 (s, 1H), 7.32-7.07 (m, 6H), 6.81 (d, *J* = 10.8 Hz, 1H), 5.24 (t, *J* = 7.7 Hz, 1H), 3.74-3.58 (m, 2H), 3.09-2.99 (m, 1H), 2.97 (s, 3H), 2.67 (s, 3H), 2.48-2.36 (m, 2H), 1.66-1.45 (m, 1H), 1.35 (d, *J* = 6.9 Hz, 6H) ppm.

^{13}**C NMR** (75 MHz, $CDCl_3$) δ = 147.0, 145.3, 139.3, 137.9, 137.6, 134.6, 133.5, 132.6, 129.1, 128.4, 128.3, 126.8, 125.6, 124.7, 61.5, 41.4, 41.2, 37.6, 27.9, 24.6, 13.2 ppm.

HRMS (EI) m/z: $[M + H]^+$ Calcd for $C_{24}H_{29}O$: 333.2213; Found: 333.2210.

11. Synthesis of 3,3-Di(azulen-1-yl)oxetane (6)

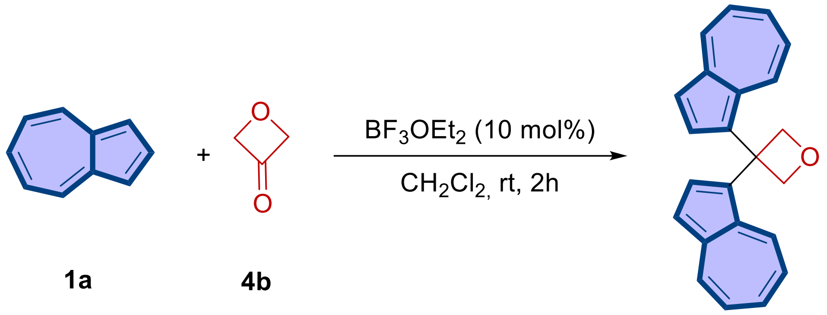

1a **4b**

6 (65%)

$F_3B{\cdot}OEt_2$ (2.5 μL, 0.02 mmol, 10 mol%) was added at room temperature to a solution of azulene (**1a**, 76.9 mg, 0.6 mmol) and oxetan-3-one (**4b**, 14.4 mg, 0.2 mmol) in CH_2Cl_2 (1 mL). The reaction mixture was stirred until complete consumption of **4b** as monitored by TLC (2 hours). The solvent was then removed under reduced pressure, and the crude residue was purified by column chromatography (silica gel, hexanes/ethyl acetate 5:1) to afford 3,3-di(azulen-1-yl)oxetane (**6**, 40.4 mg, 65%) as a blue oil.

^{1}H NMR (300 MHz, $CDCl_3$): δ = 8.34 (t, *J* = 9.5 Hz, 4H), 7.65 (d, *J* = 3.9 Hz, 2H), 7.55 (t, *J* = 9.9 Hz, 2H), 7.32 (d, *J* = 3.9 Hz, 2H), 7.13 (t, *J* = 9.7 Hz, 2H), 7.03 (t, *J* = 9.8 Hz, 2H), 5.76 (s, 4H) ppm.

^{13}C NMR (75 MHz, $CDCl_3$): δ = 141.5, 137.8, 137.3, 136.8, 135.0, 134.7, 134.4, 123.0, 122.4, 116.3, 85.8, 45.9 ppm.

HRMS (EI) m/z: $[M + Na]^+$ Calcd for $C_{23}H_{18}NaO$: 332.1250; Found: 332.1255.

12. References

1. Anderson, A. G.; Nelson, J. A.; Tazuma, J. M. Azulene. III. Electrophilic Substitution, J. Am. Chem. Soc. **1953**, *75*, 4980-4989.

2 a) Gudla, V.; Balamurugan, R. Synthesis of 1-Arylnaphthalenes by Gold-Catalyzed One-Pot Sequential Epoxide to Carbonyl Rearrangement and Cyclization with Arylalkynes. *Chem. Asian J.* 2013, *8*, 414-428; b) Sakakibara, K.; Nakano, K.; Nozaki, K. Regio-controlled ring-opening polimerization of perfluoroalkyl-substituted epoxides. *Chem. Commun.* **2006**, 3334-3336; c) Li, L.; Sivaguru, P.; Wei, D.; Liu, M.; Zhu, Q.; Dong, S.; Casali, E.; Li, N.; Zanoni, G.; Bi, X. Silver-Catalyzed Direct Conversion of Epoxides into Cyclopropanes Using N-Triftosylhydrazones. *Nat. Commun.* **2024**, *15*, 1951; d) Chen, E. F. W.; Li, C.-J. Palladium-Catalysed Tsuji-Trost-Type Vinyl Epoxide Cross-Coupling with Umpolung Hydrazones. *Chem. Sci.* **2024**. 15, 18943; e) Minkler, S. R. K.; Isley, N. A.; Lippincott, D. J.; Krause, N.; Lipshutz, B. H. Leveraging the Micellar Effect: Gold-Catalyzed Dehydrative Cyclizations in Water at Room Temperature. *Org. Lett.* **2014**, 16, 724.

3. Kwon, D. W.; Kim, Y. H.; Lee, K. Highly Regioselective Cleavages and Iodinations of Cyclic Ethers Utilizing SmI_2. *J. Org. Chem.* **2002**, *67*, 9488

^{1}H NMR of compound 3aa (300 MHz, $CDCl_3$)

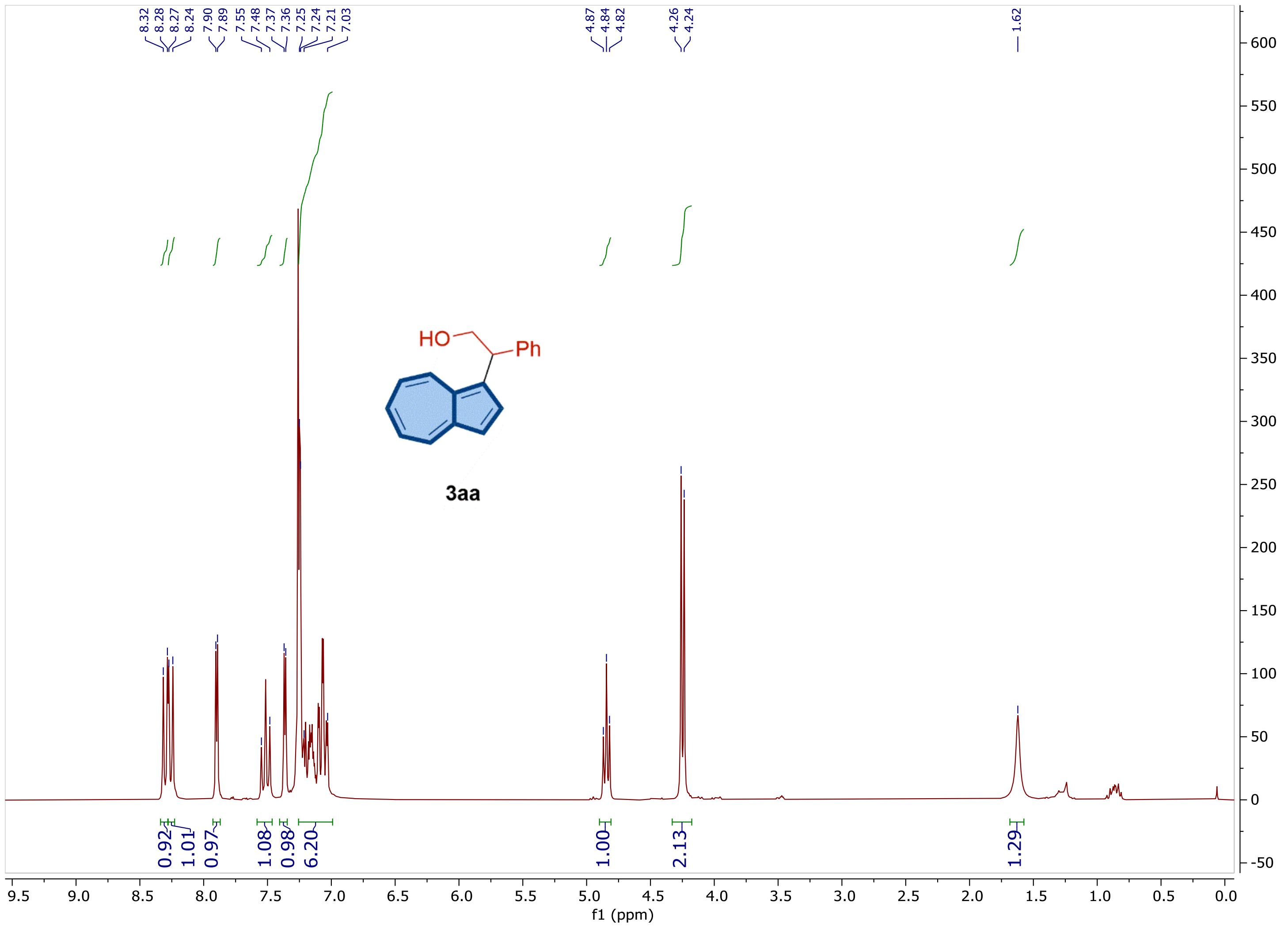

^{13}C NMR of compound 3aa (75 MHz, $CDCl_3$)

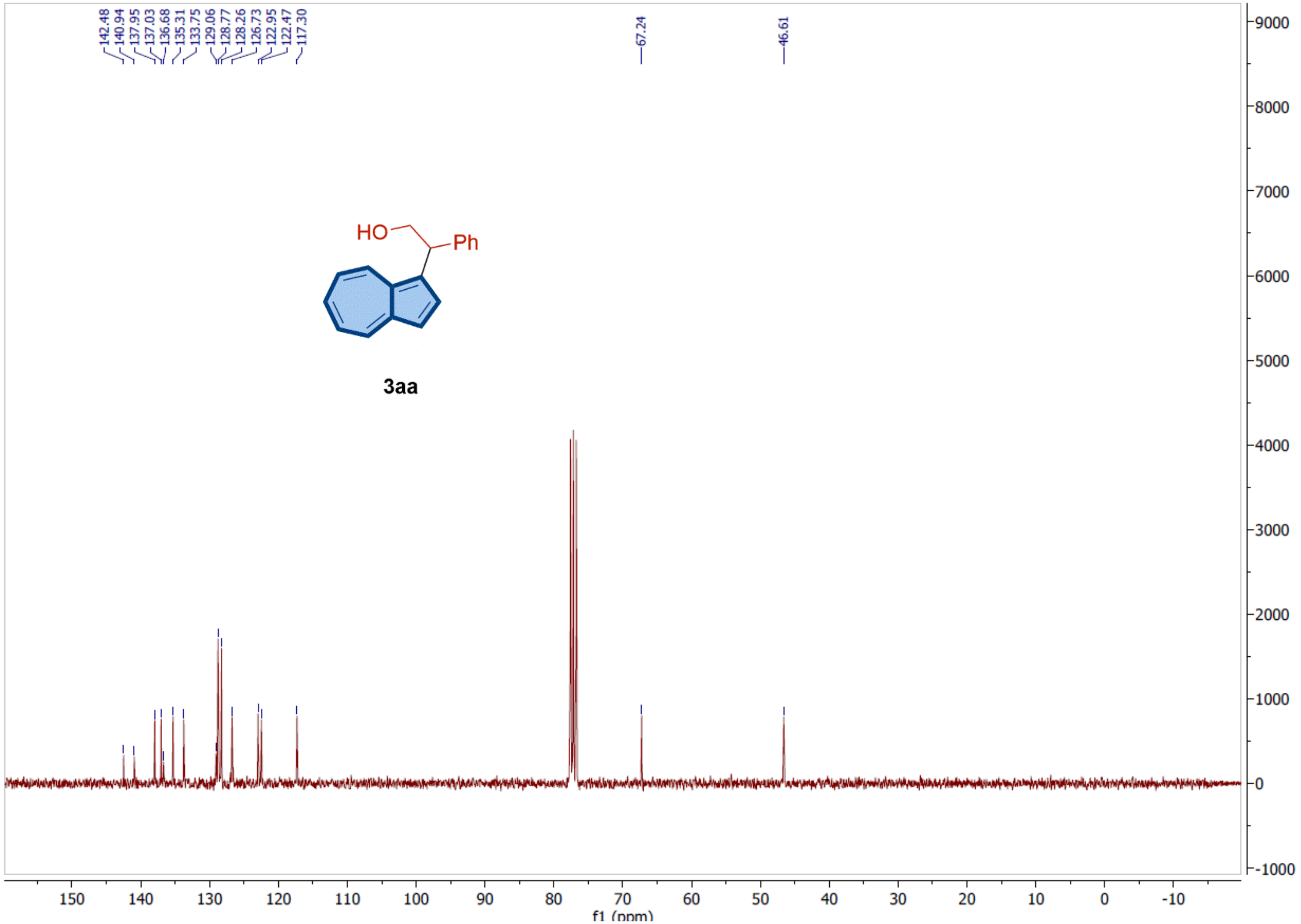

¹H NMR of compound 3ab (300 MHz, $CDCl_3$)

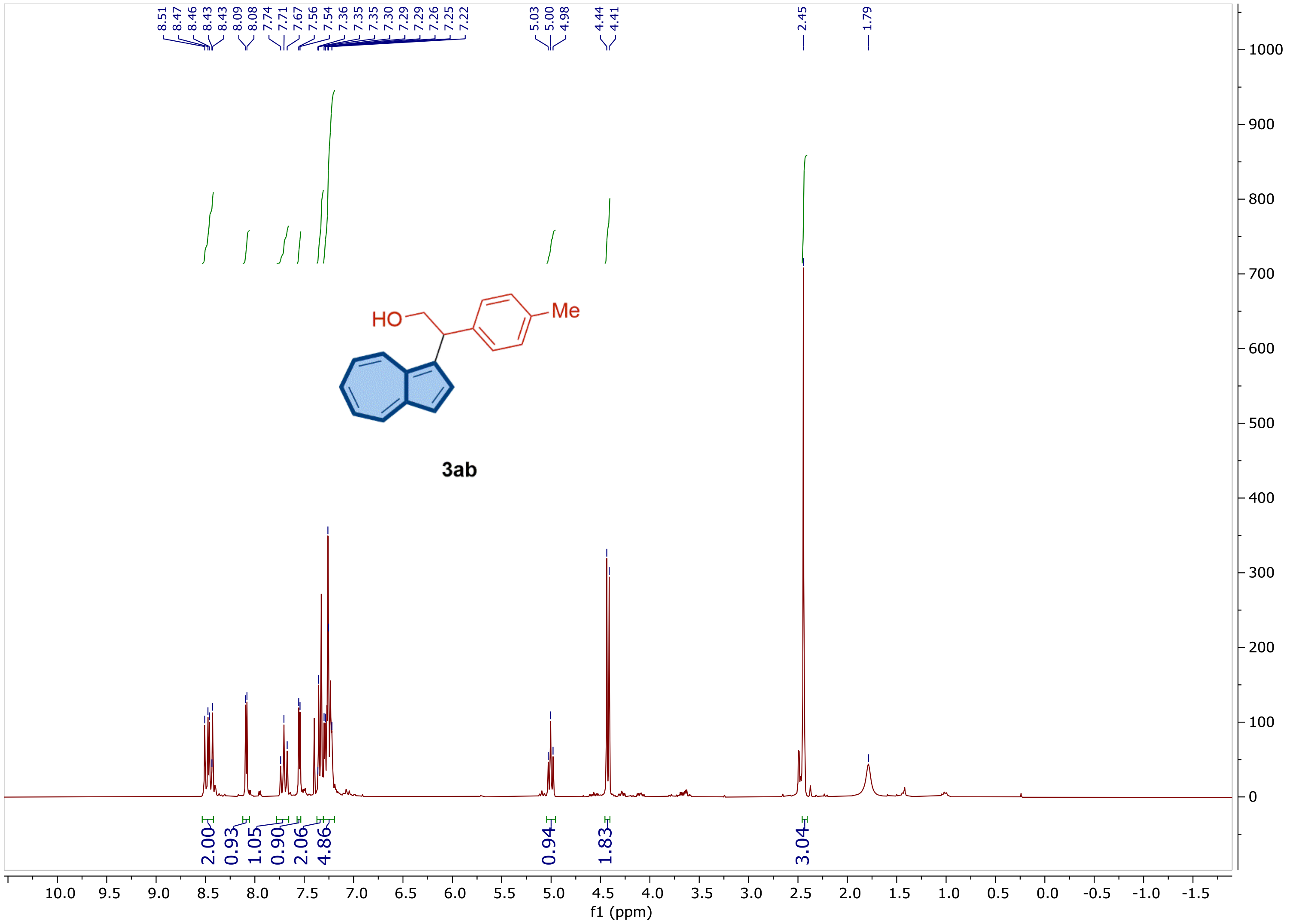

^{13}C NMR of compound 3ab (75 MHz, $CDCl_3$)

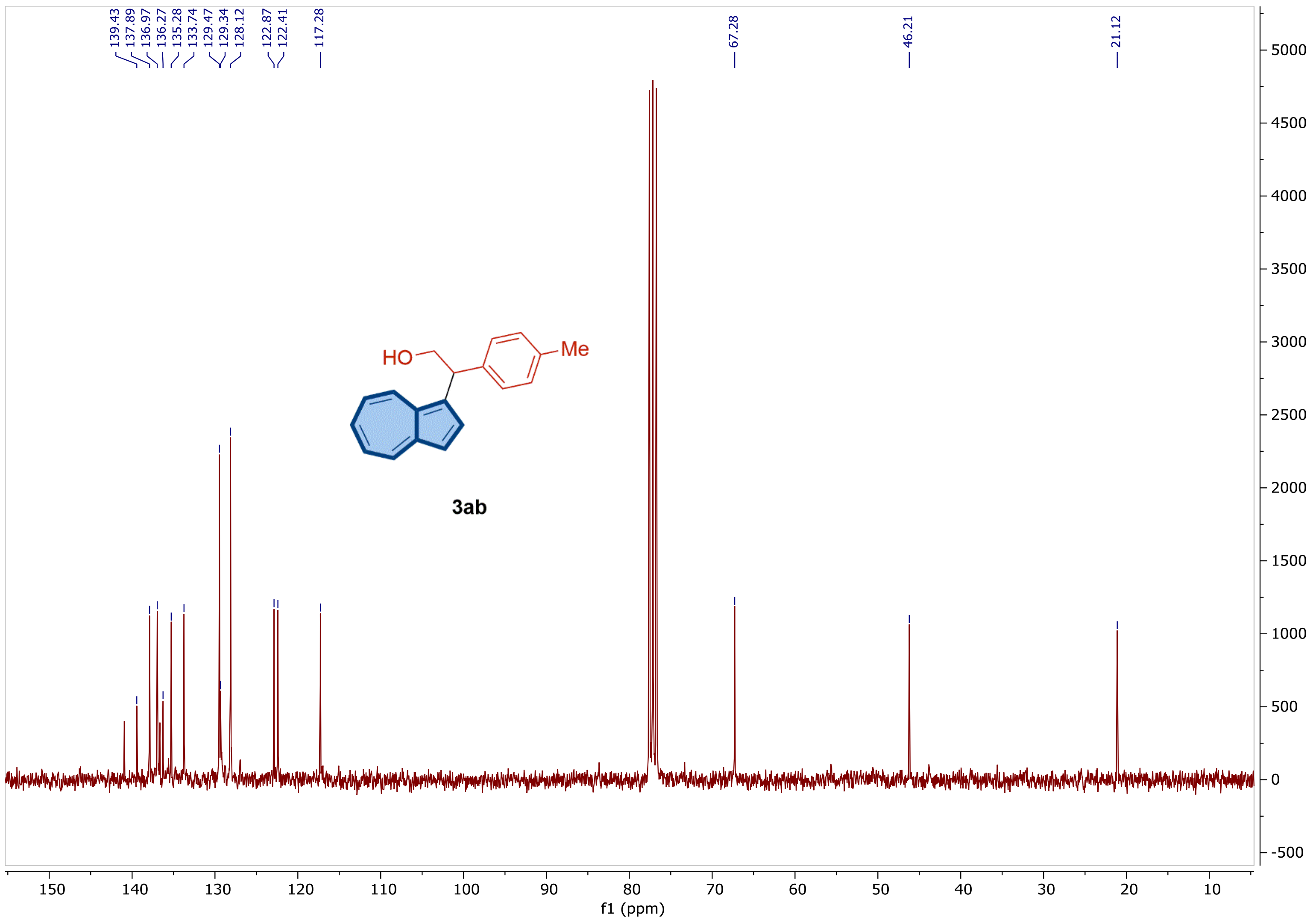

^{1}H NMR of compound 3ac (300 MHz, CDCl3)

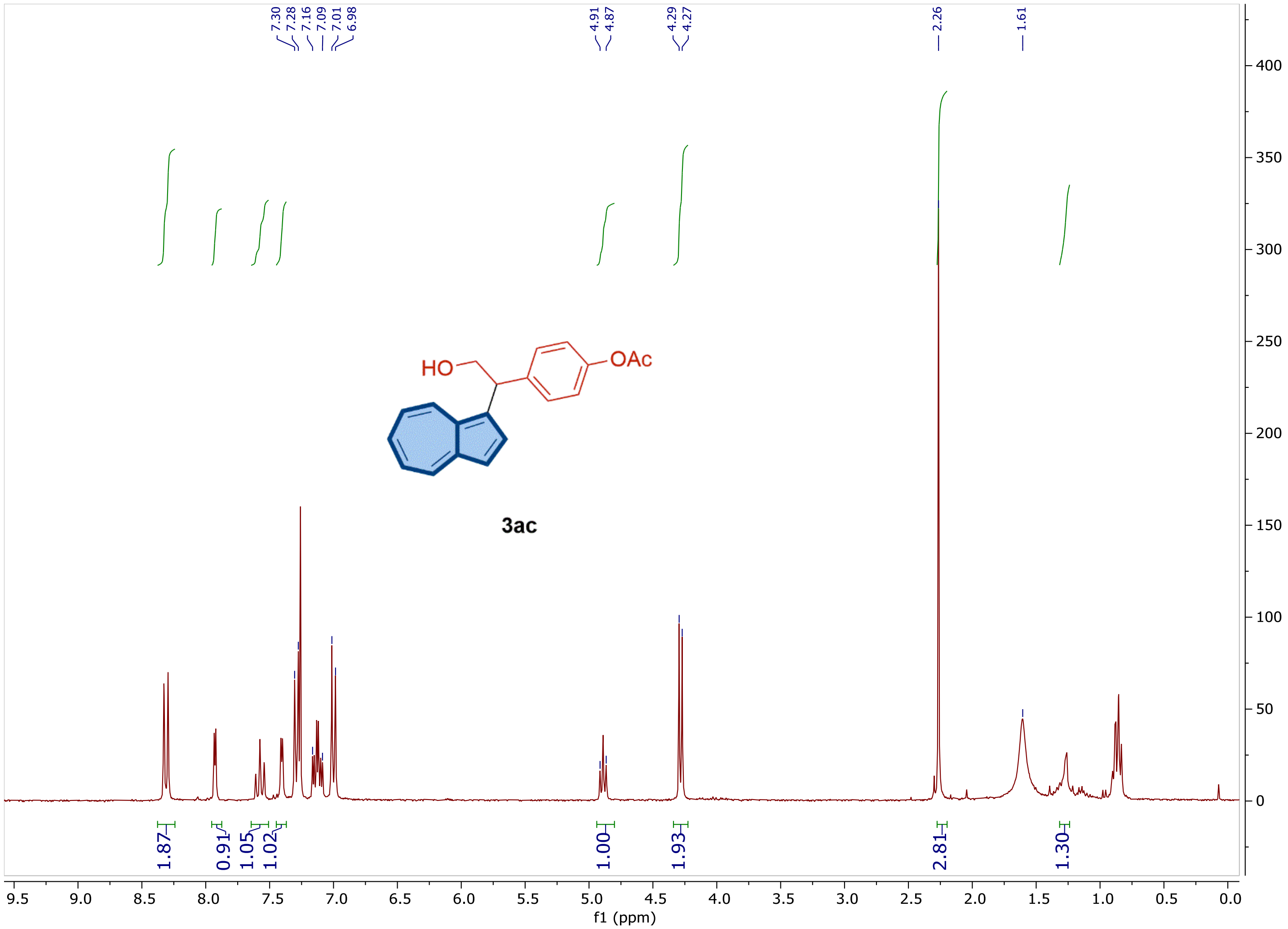

^{13}C NMR of compound 3ac (75 MHz, CDCl$_3$)

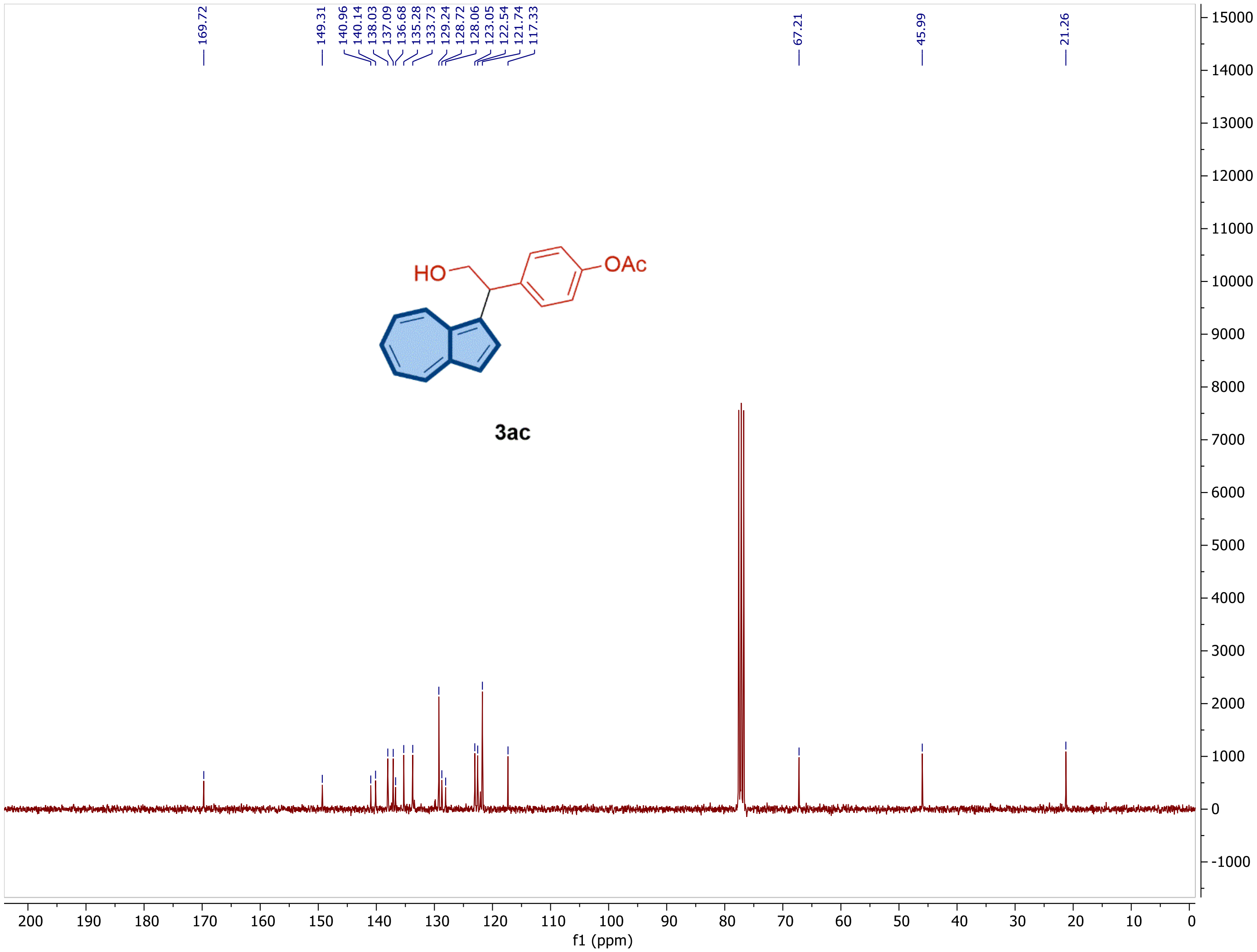

^{1}H NMR of compound 3ad (300 MHz, $CDCl_3$)

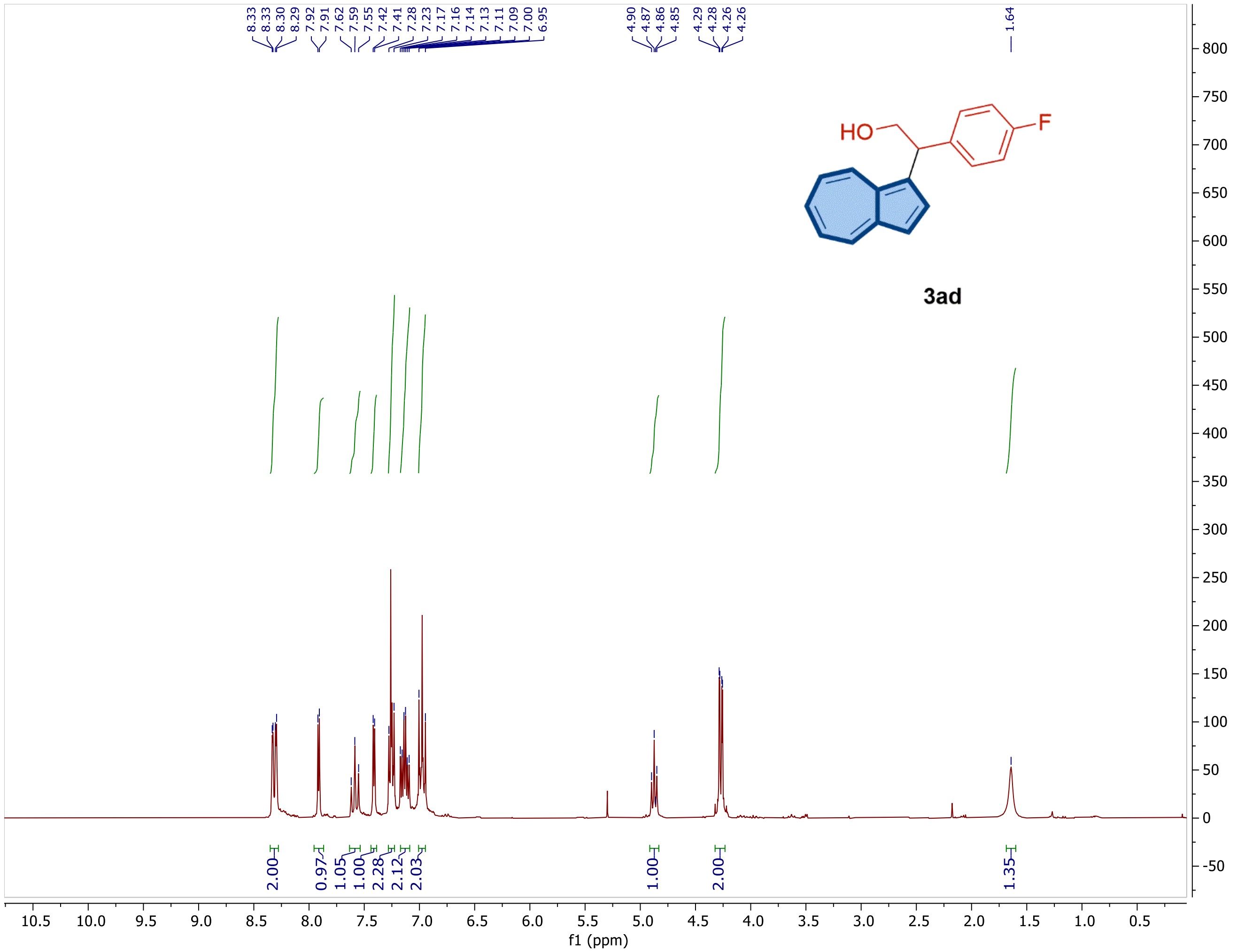

^{13}C NMR of compound 3ad (75 MHz, $CDCl_3$)

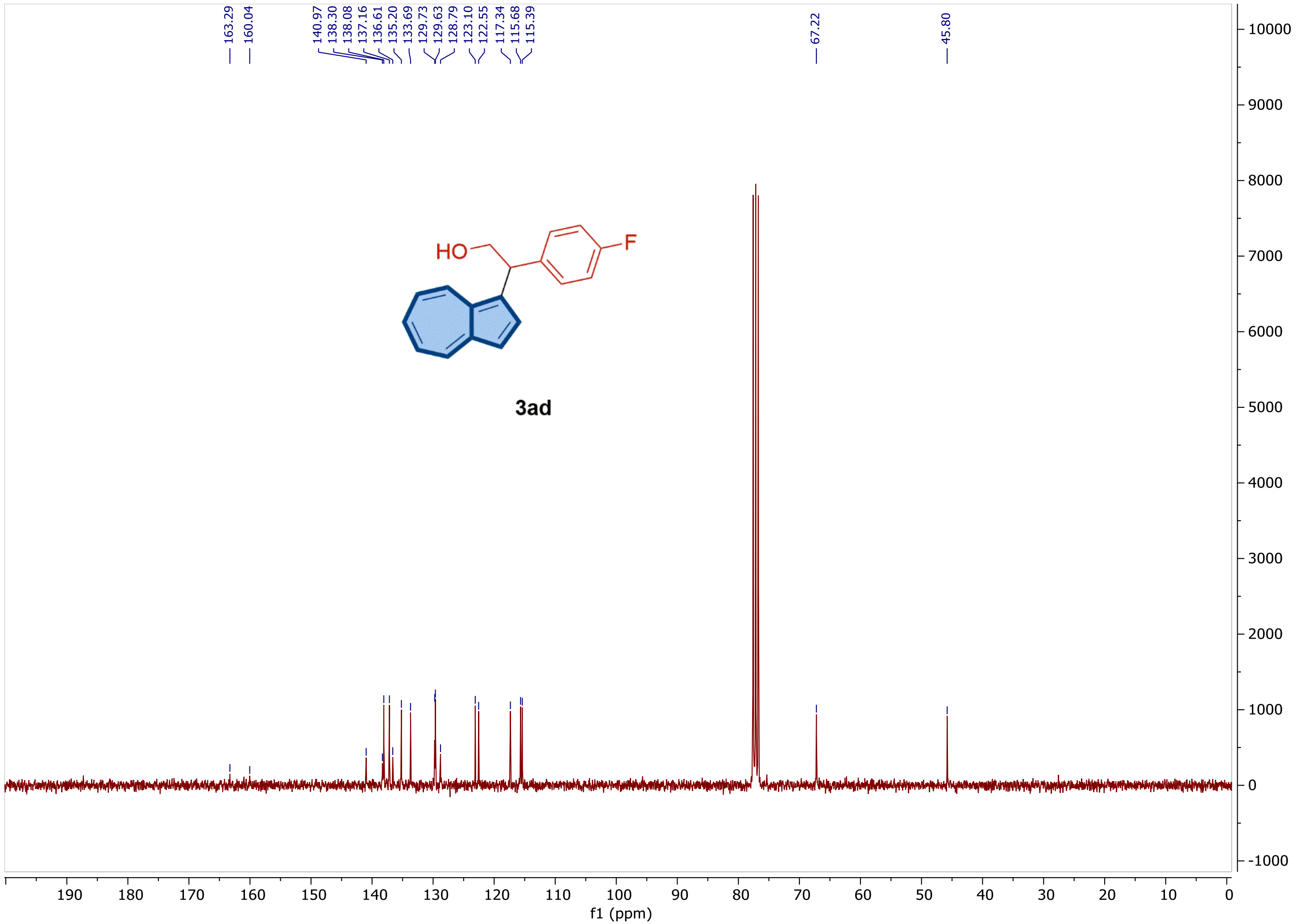

^{19}F NMR of compound 3ad (282 MHz, $CDCl_3$)

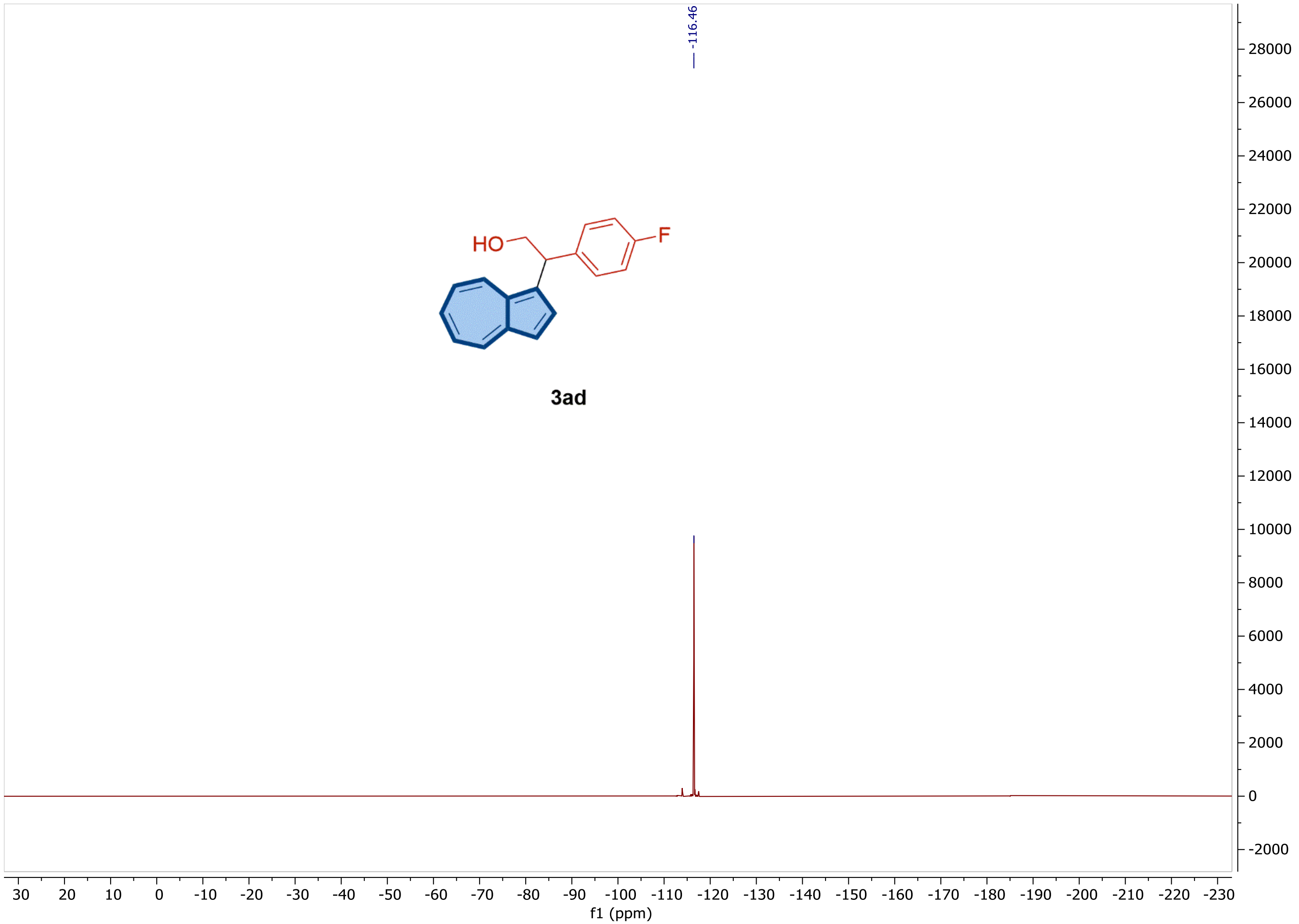

^{1}H NMR of compound 3ae (300 MHz, $CDCl_3$)

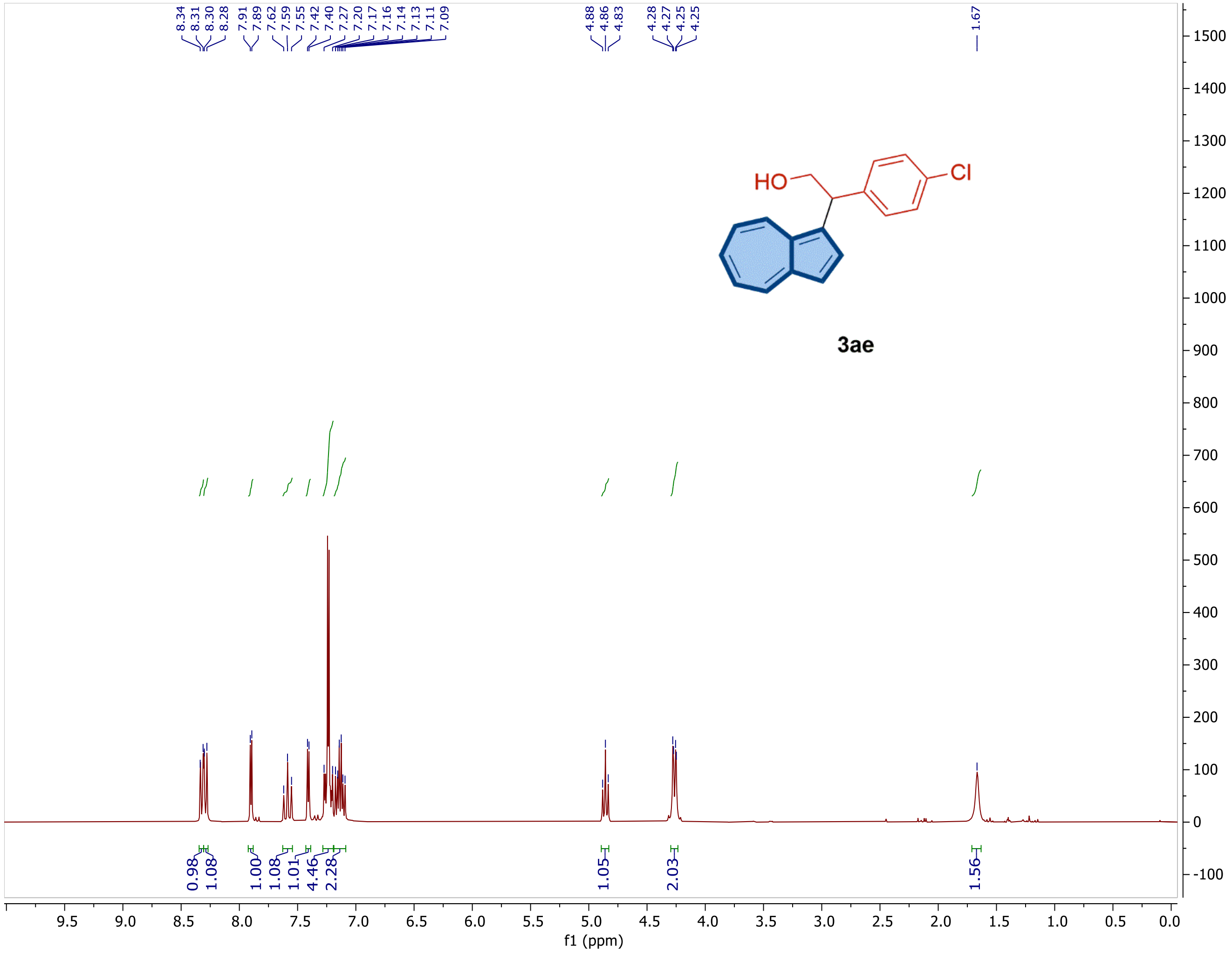

^{13}C NMR of compound 3ae (75 MHz, $CDCl_3$)

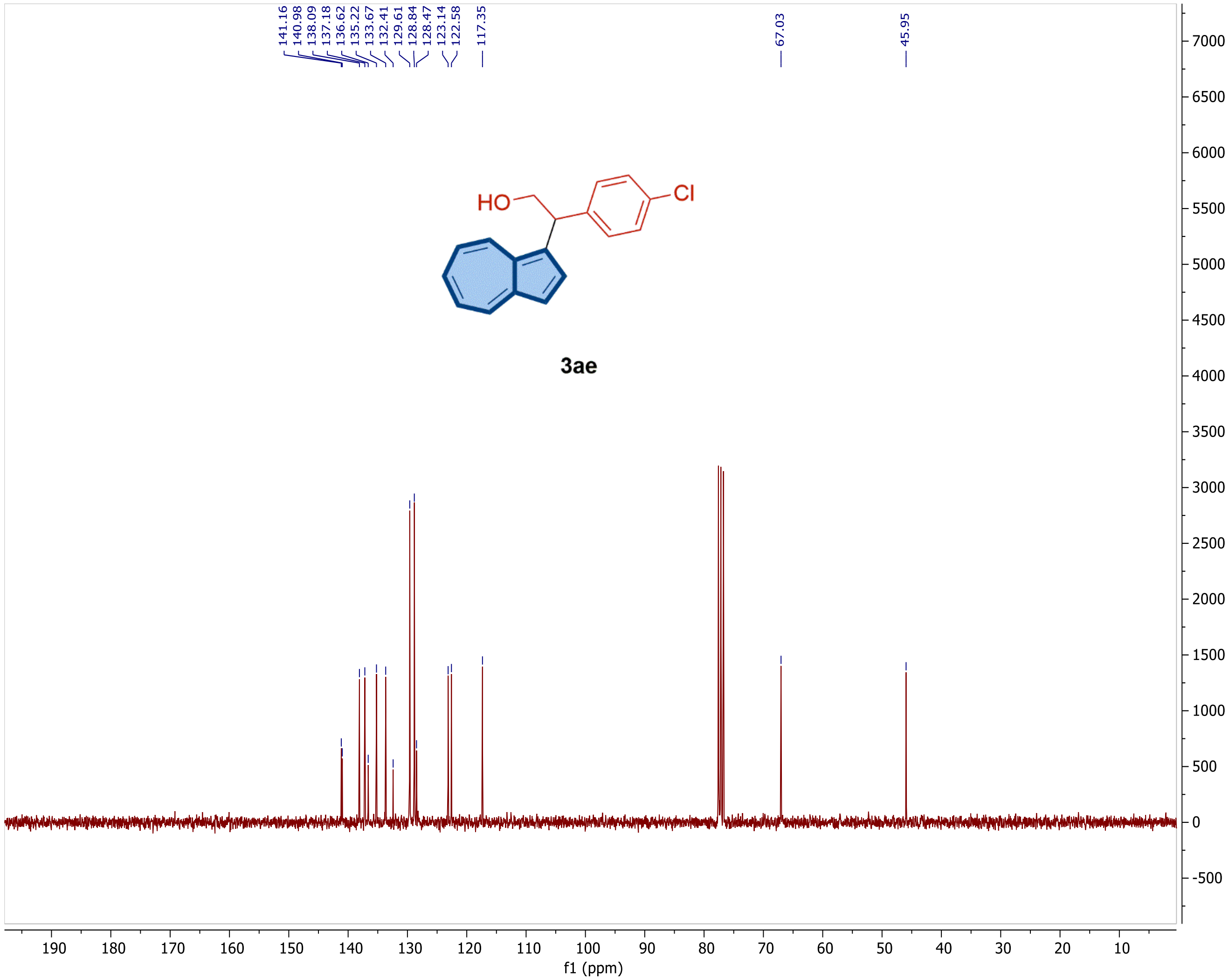

^{1}H NMR of compound 3af (300 MHz, $CDCl_3$)

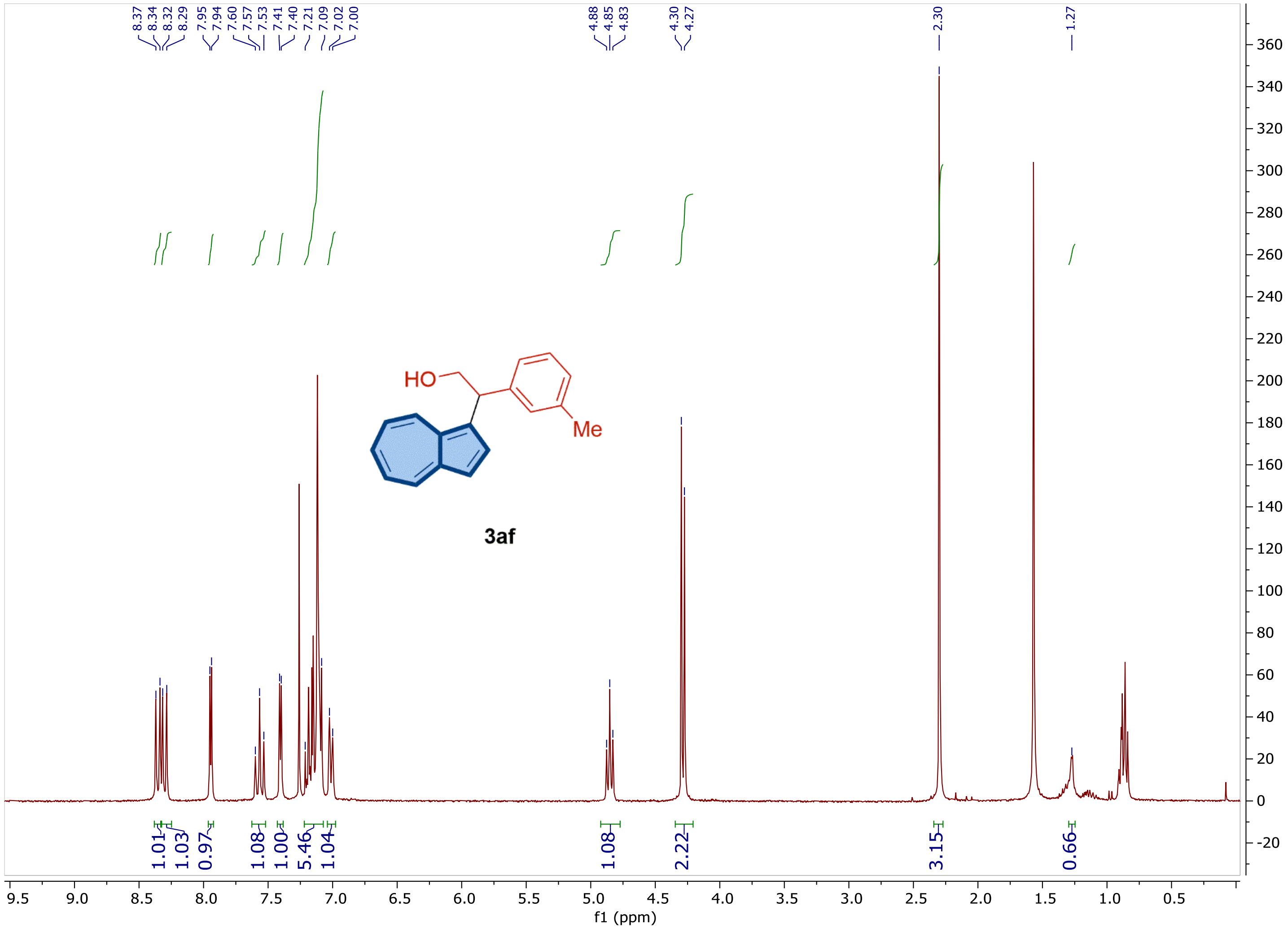

^{13}C NMR of compound 3af (75 MHz, $CDCl_3$)

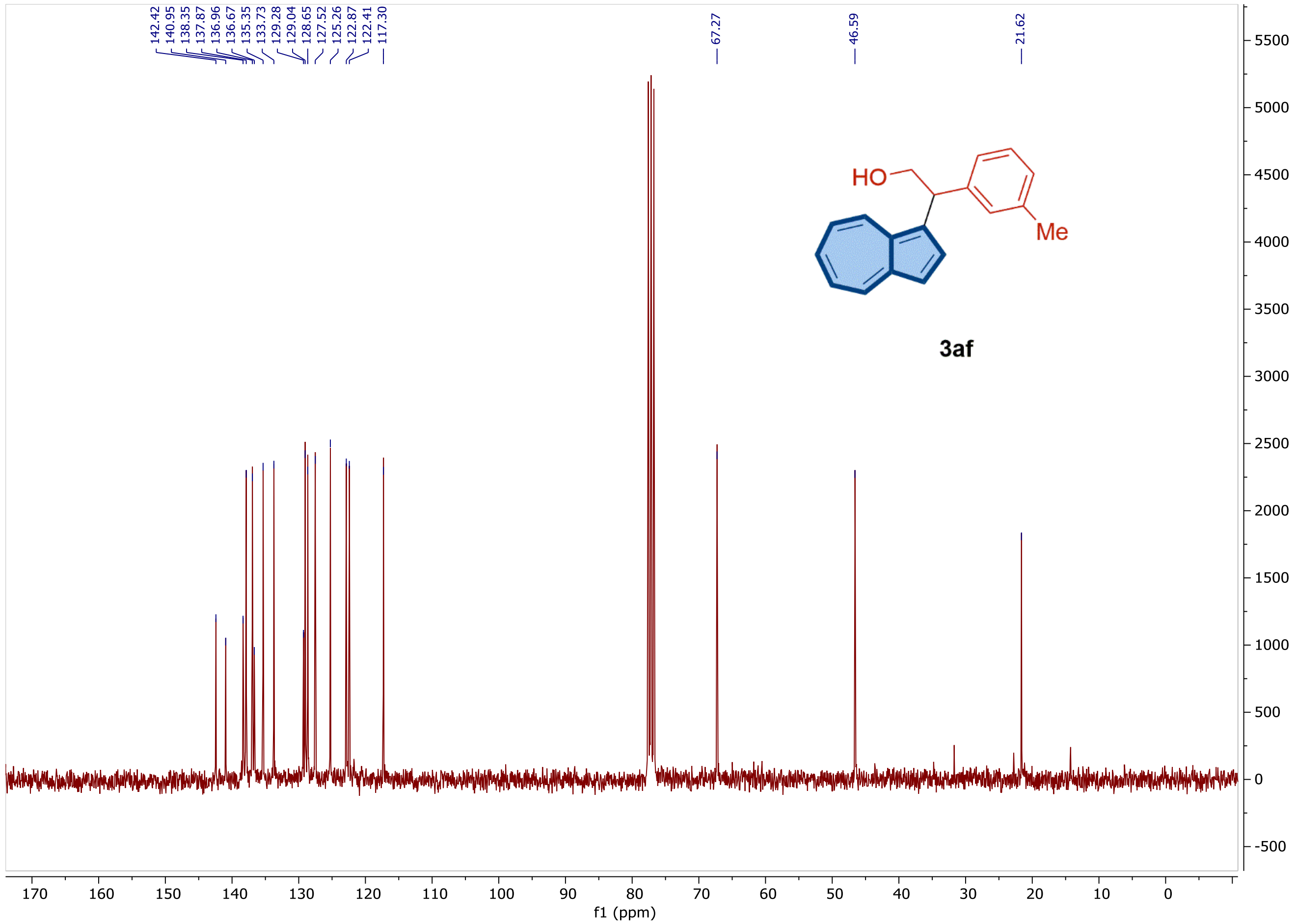

^{1}H NMR of compound 3ag (300 MHz, $CDCl_3$)

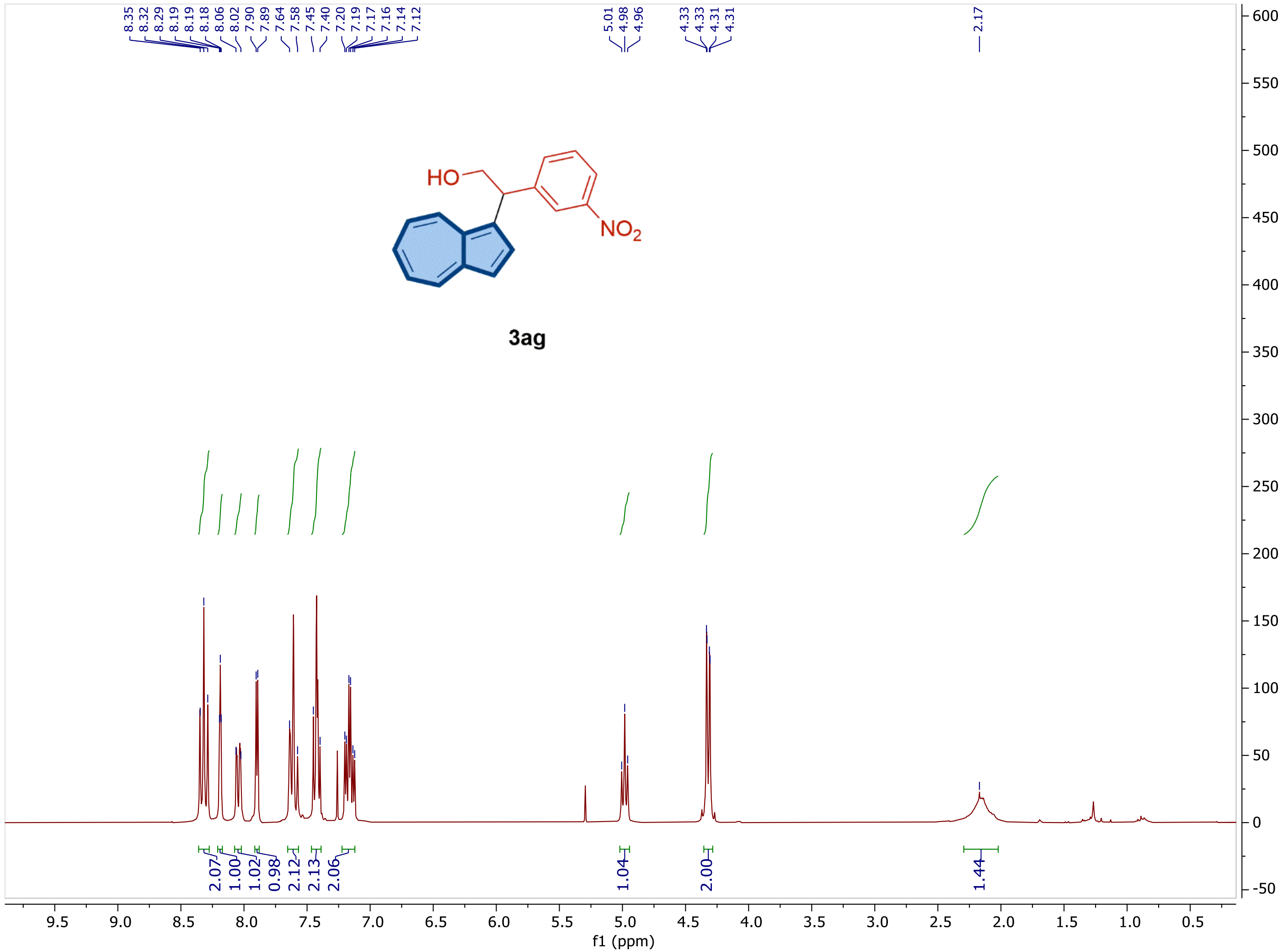

^{13}C NMR of compound 3ag (75 MHz, $CDCl_3$)

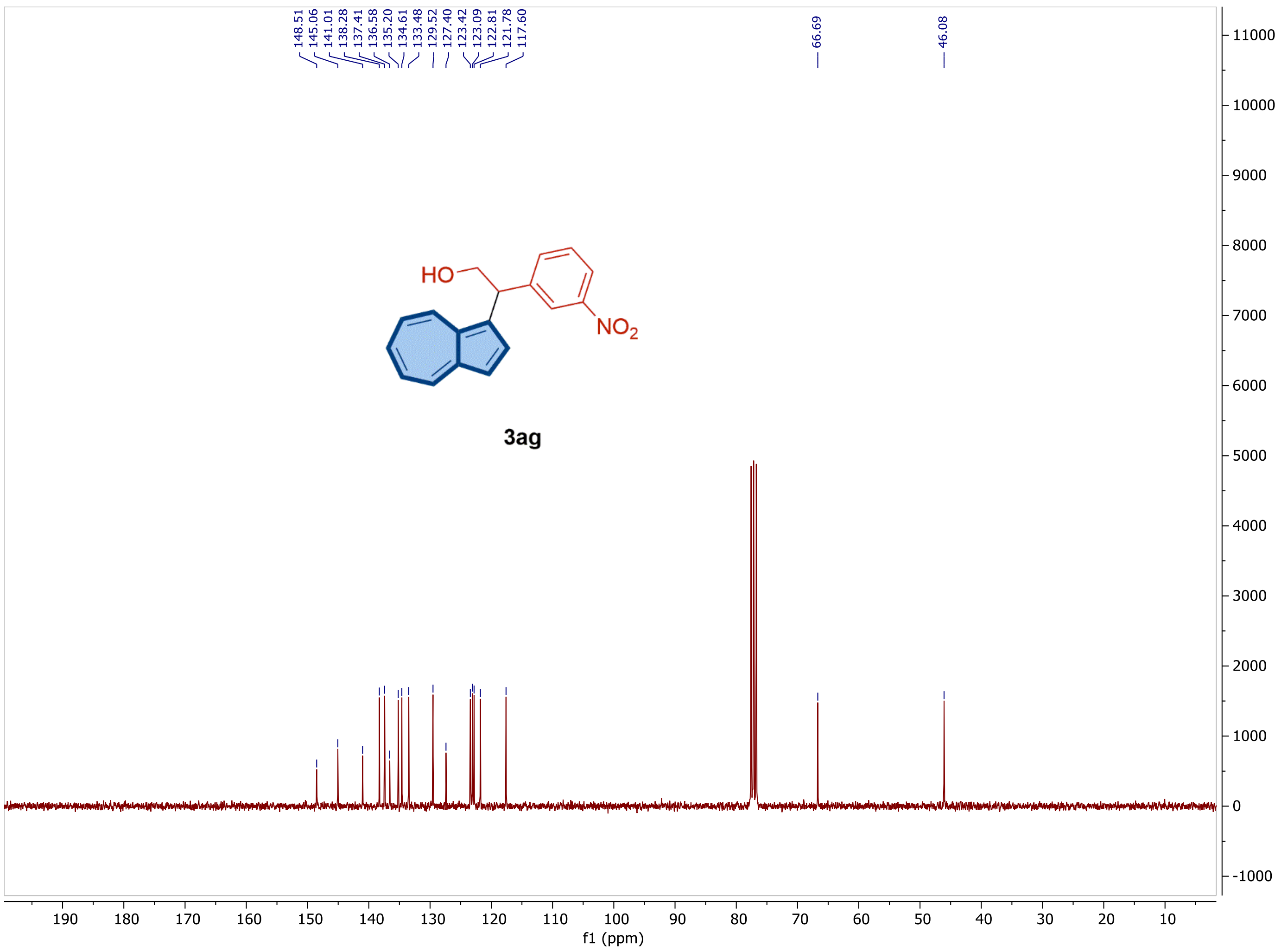

^{1}H NMR of compound 3ai (300 MHz, $CDCl_3$)

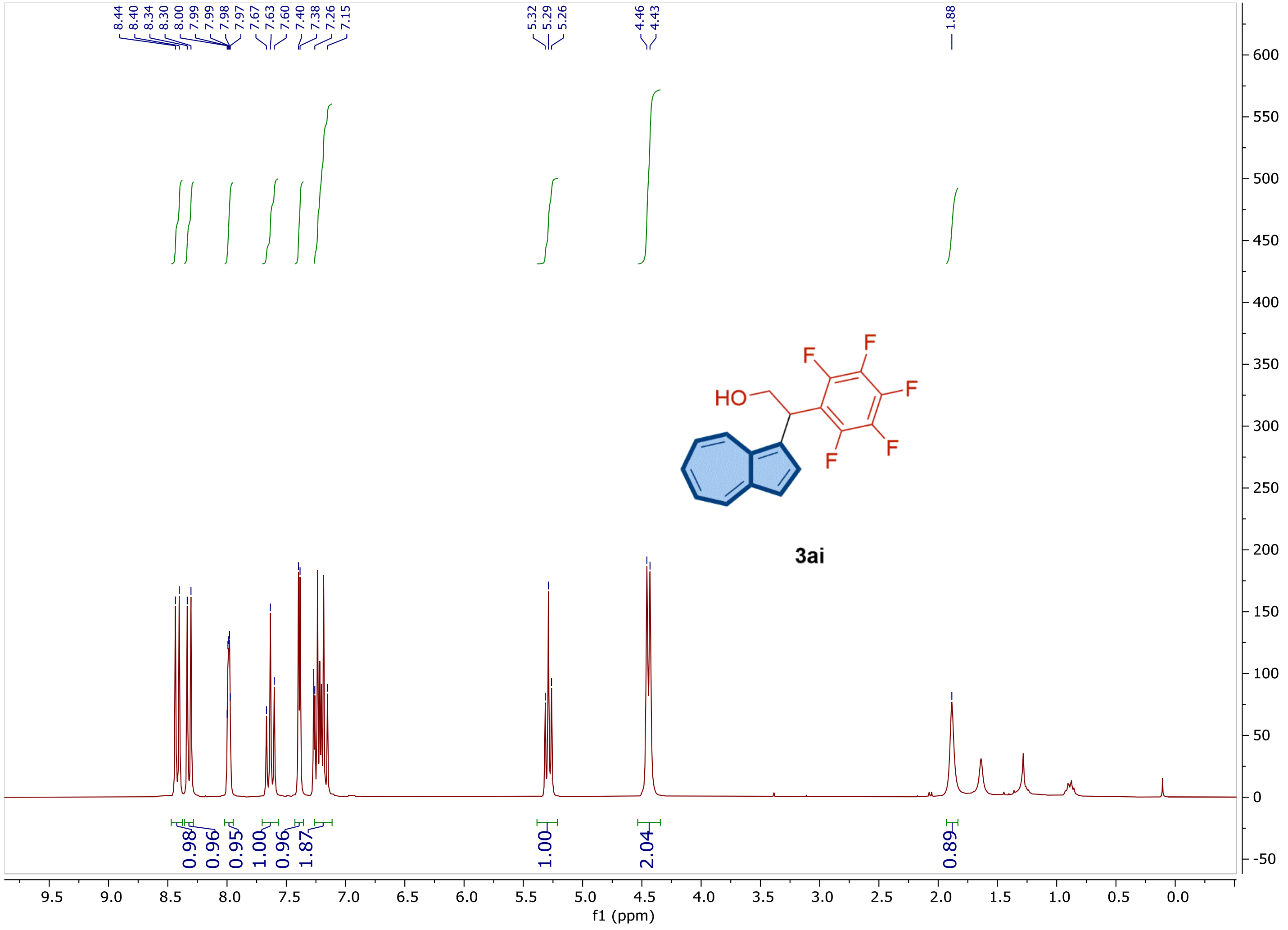

^{13}C NMR of compound 3ai (75 MHz, $CDCl_3$)

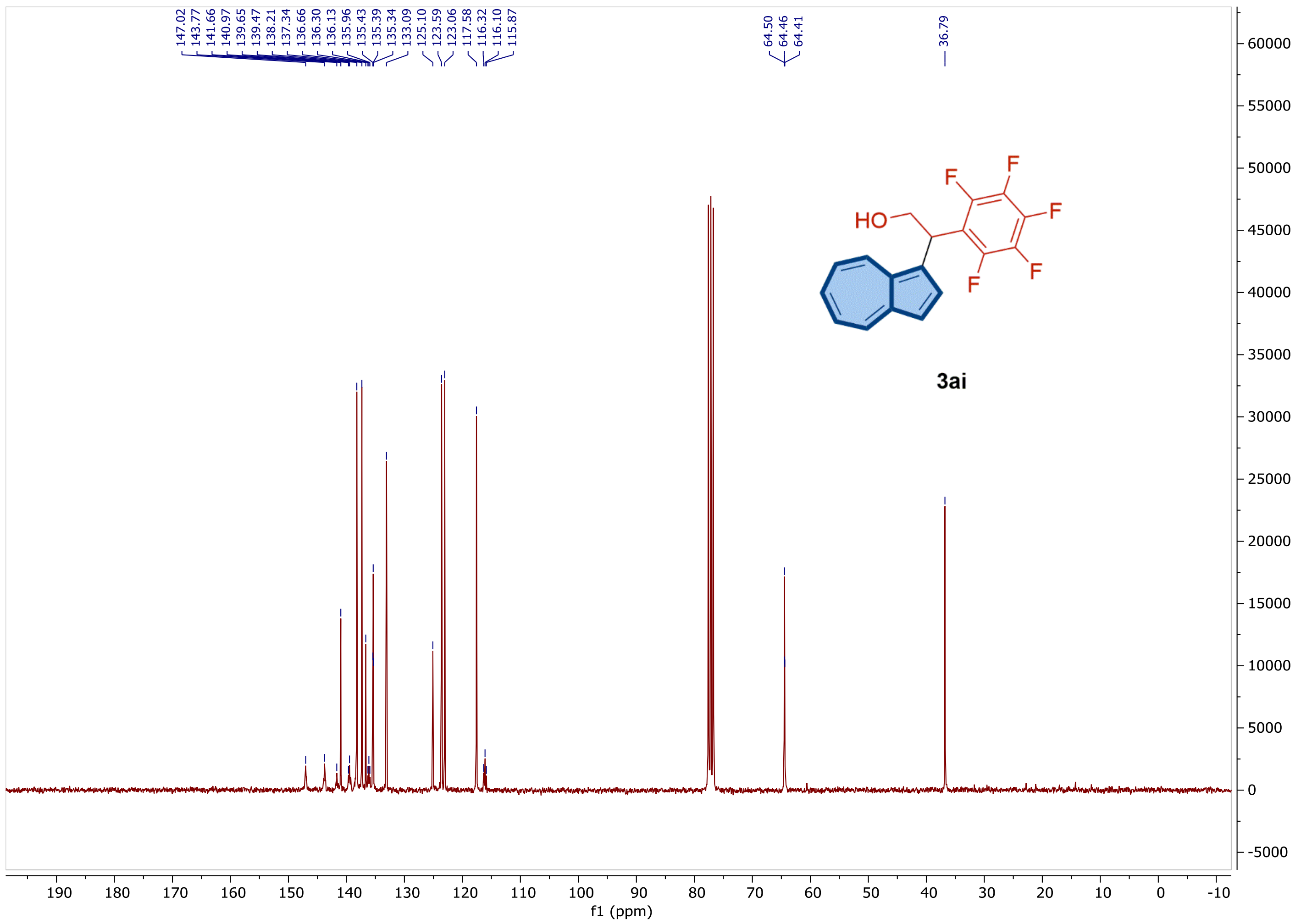

DEPT ^{13}C NMR of compound 3ai (75 MHz, $CDCl_3$)

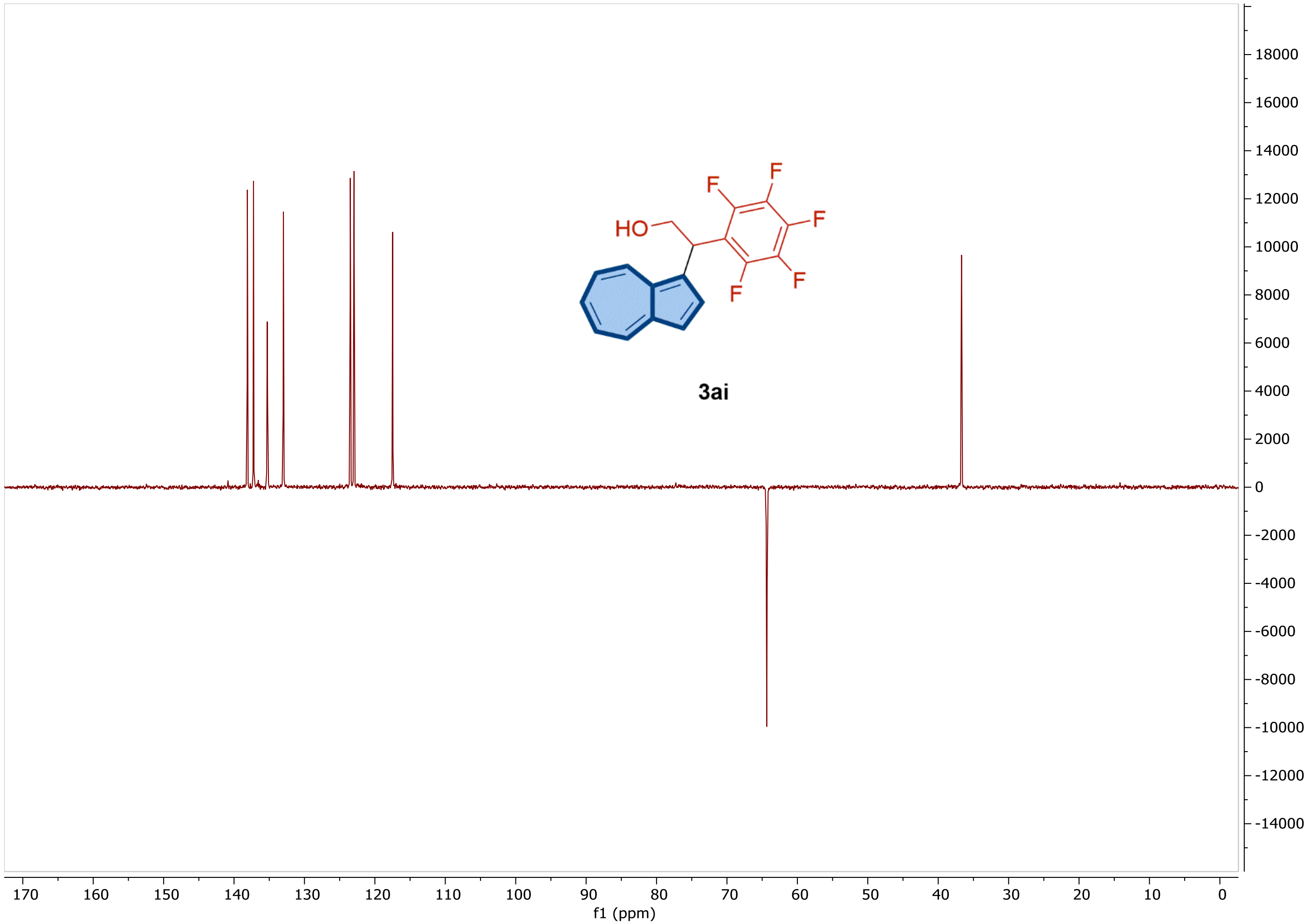

^{19}F NMR of compound 3ai (282 MHz, $CDCl_3$)

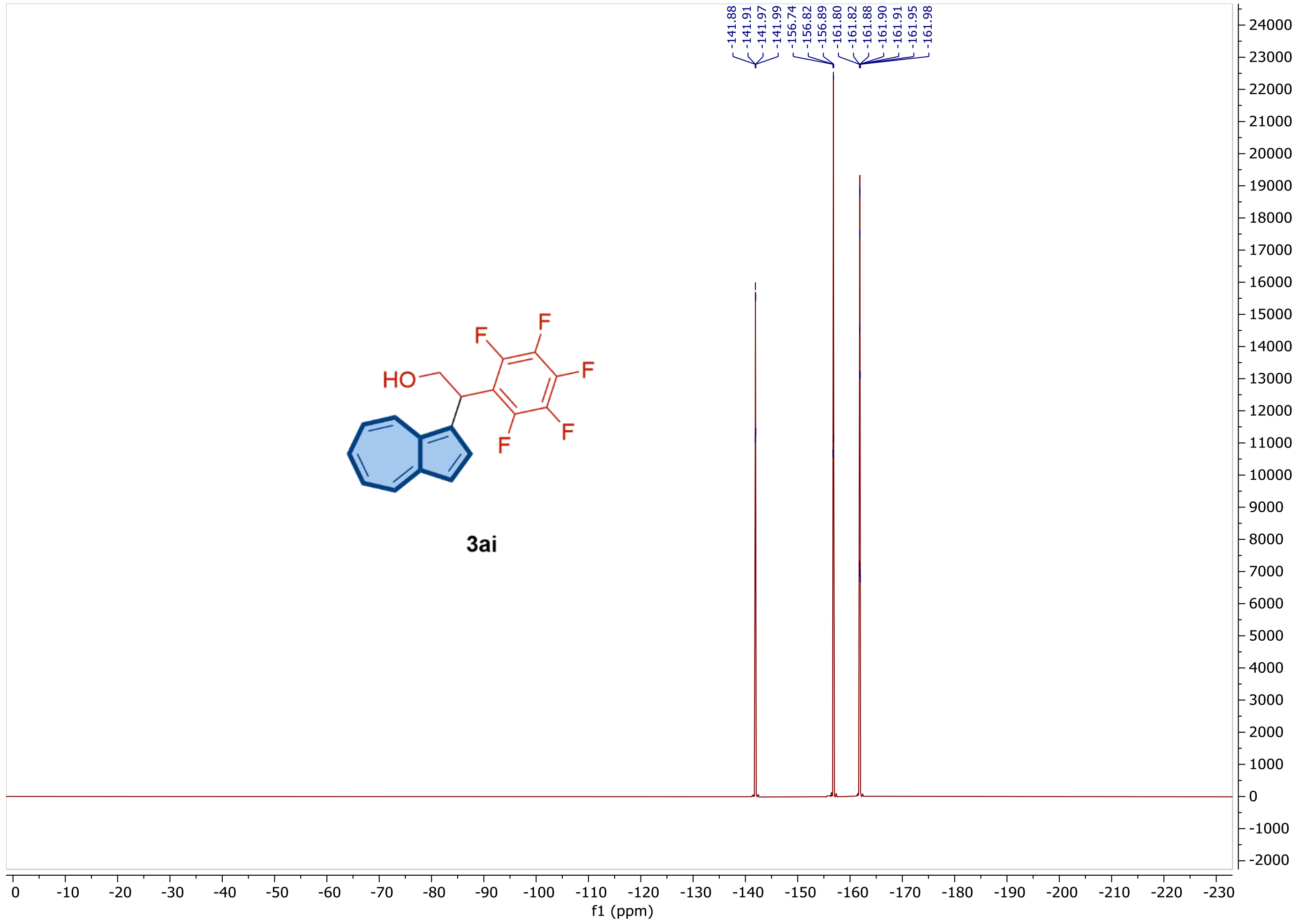

^{1}H NMR of compound 3aj (300 MHz, $CDCl_3$)

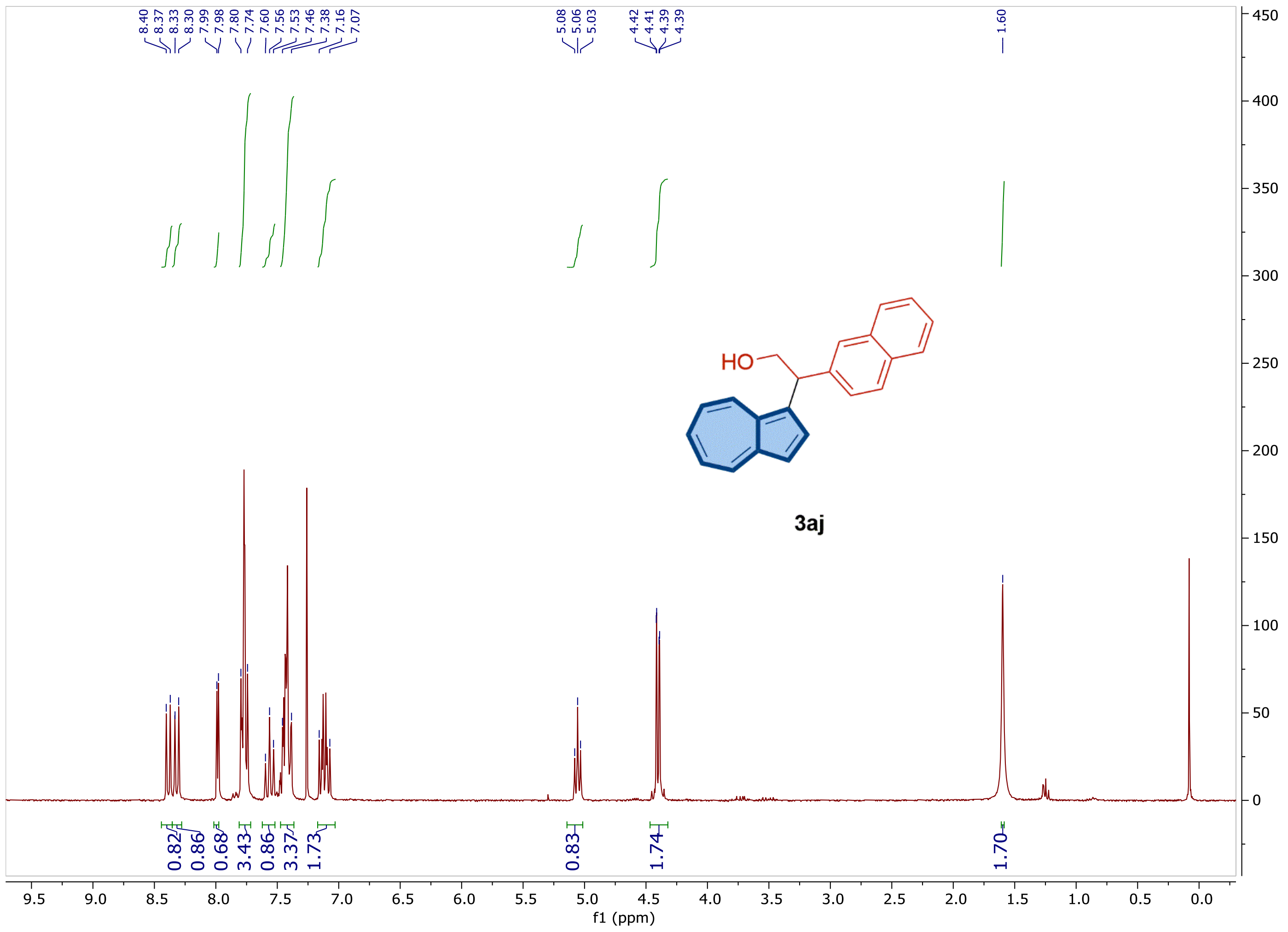

^{13}C NMR of compound 3aj (75 MHz, $CDCl_3$)

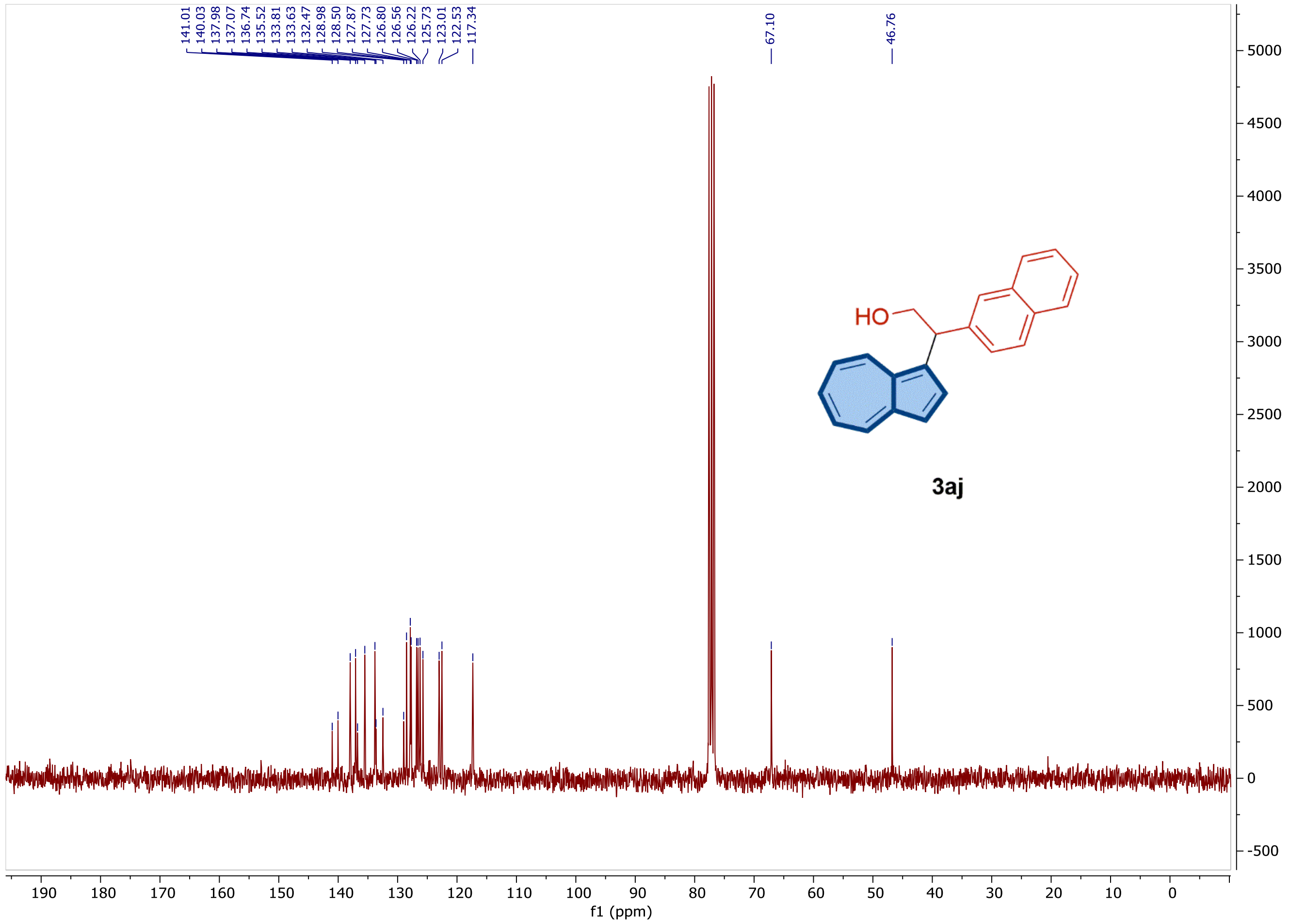

^{1}H NMR of compound 3ak (300 MHz, $CDCl_3$)

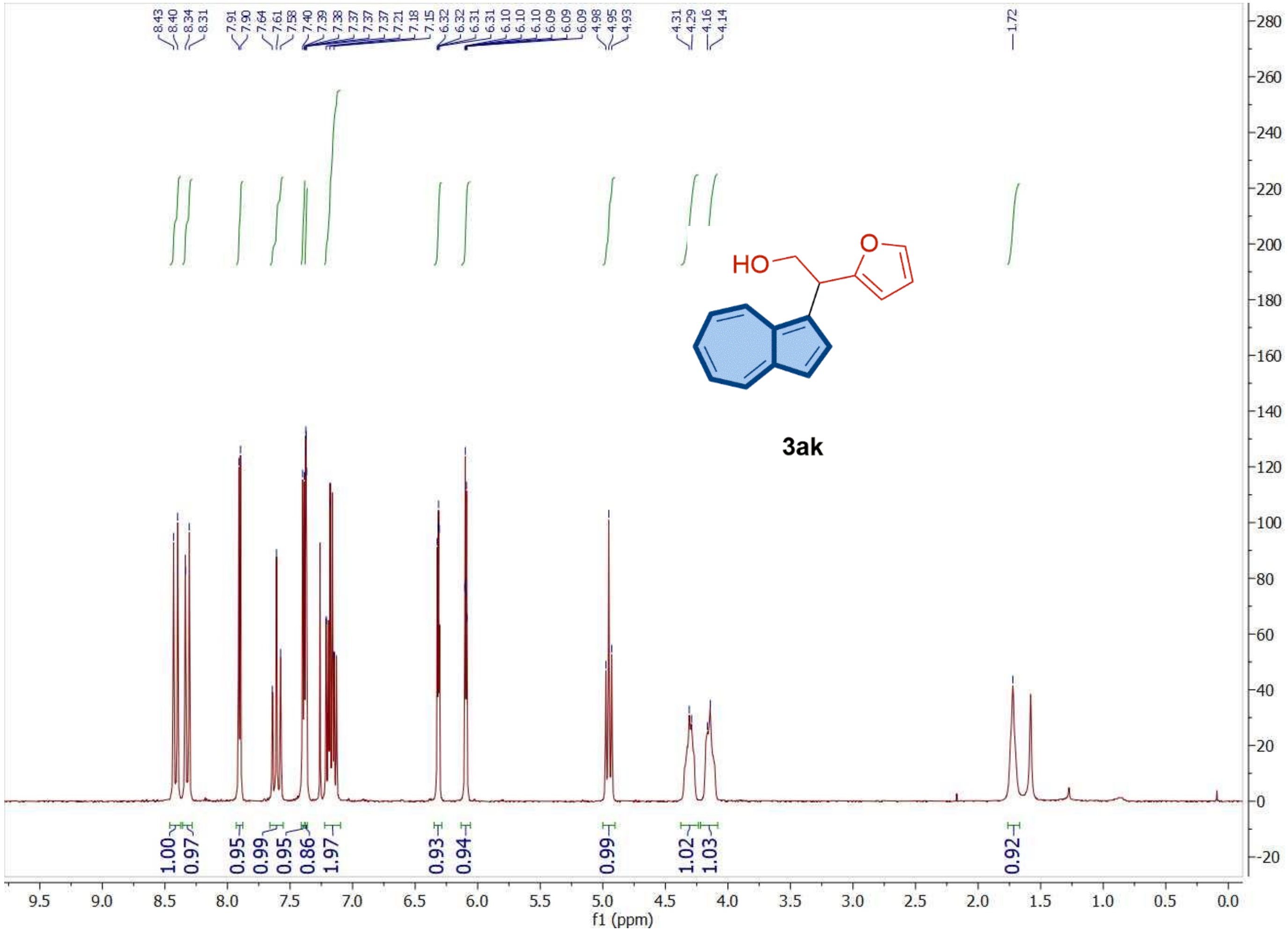

3ak

^{13}C NMR of compound 3ak (75 MHz, $CDCl_3$)

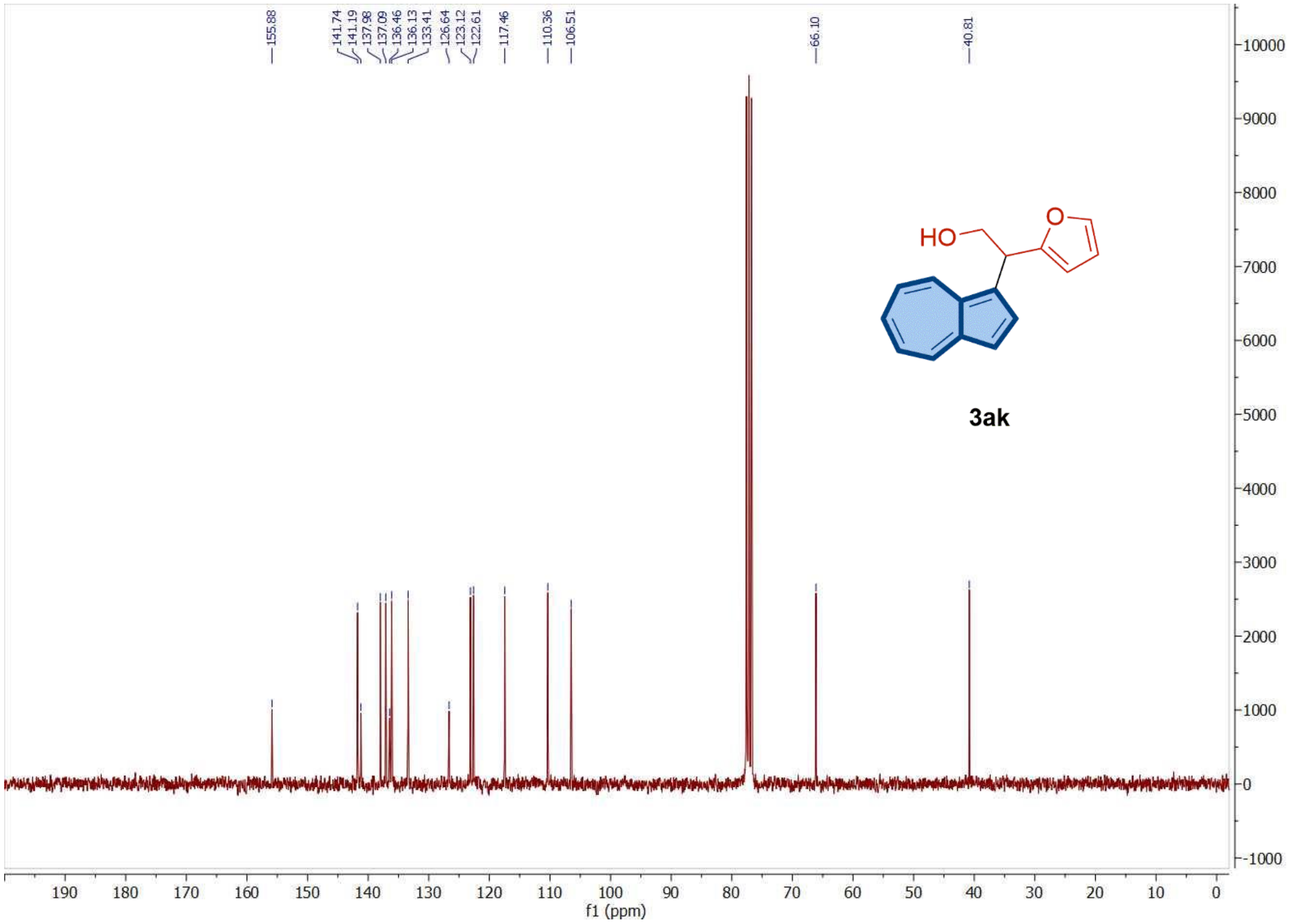

^{1}H NMR of compound 3al (300 MHz, $CDCl_3$)

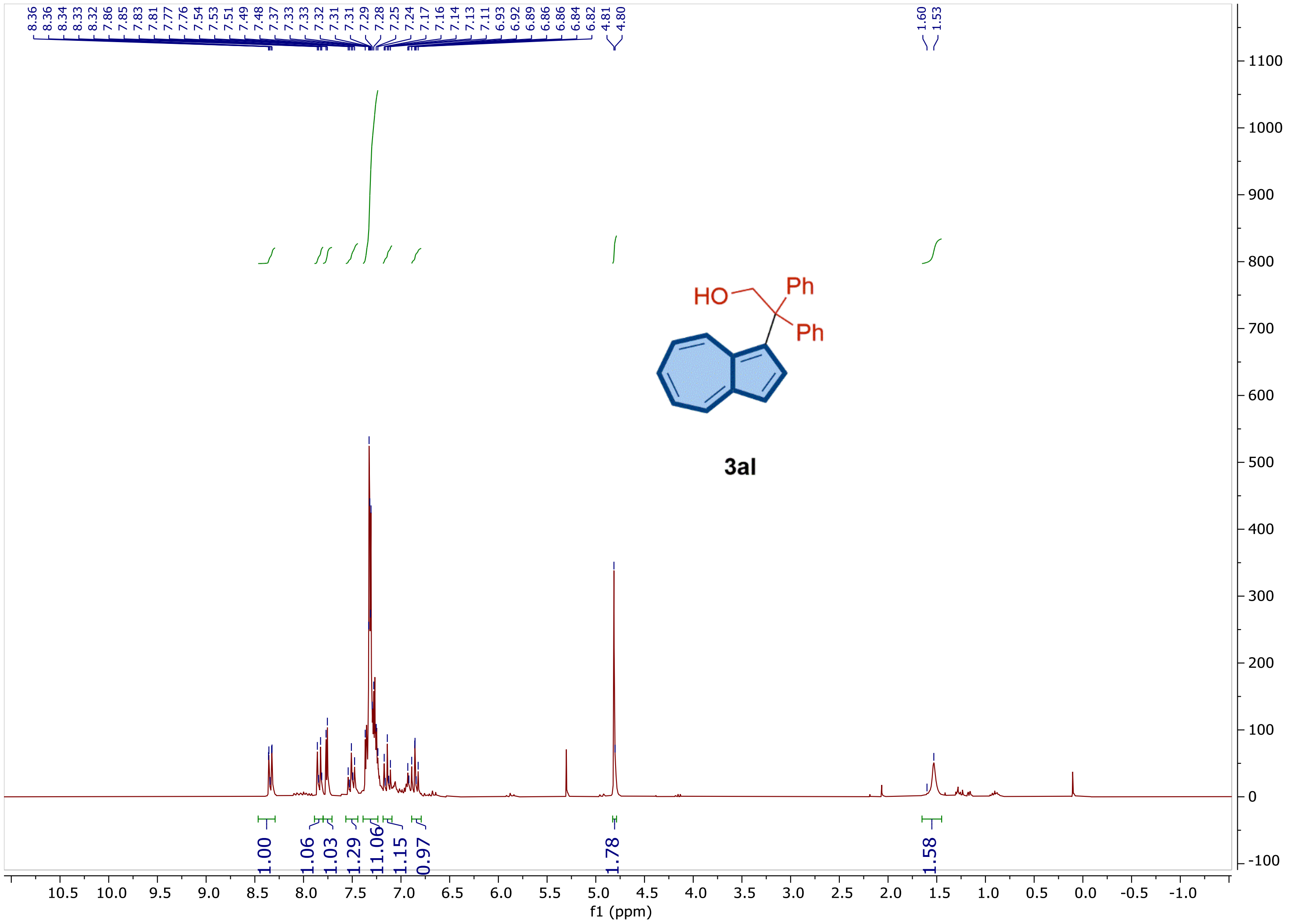

^{13}C NMR of compound 3al (75 MHz, $CDCl_3$)

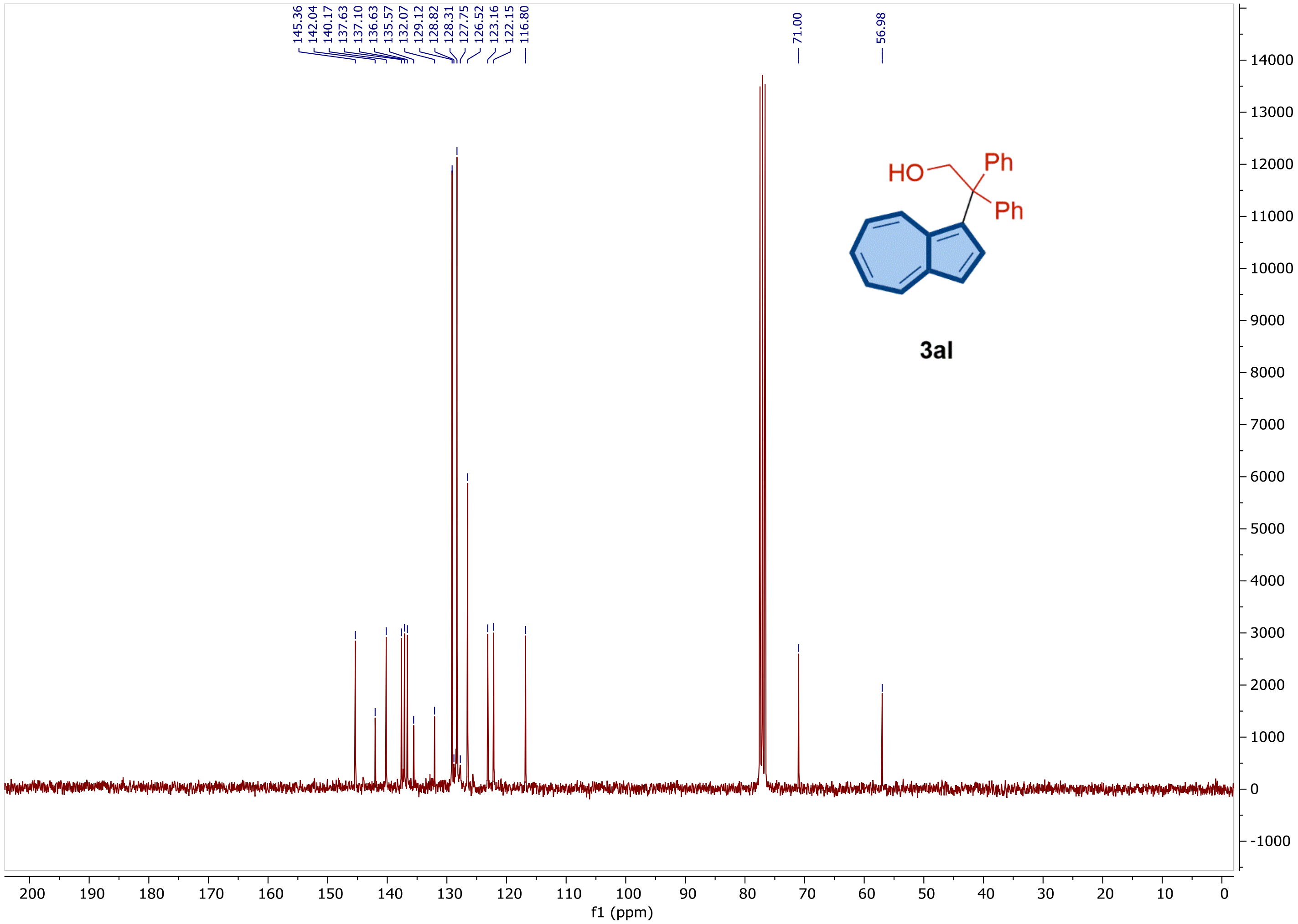

^{1}H NMR of compound 3am (300 MHz, $CDCl_3$)

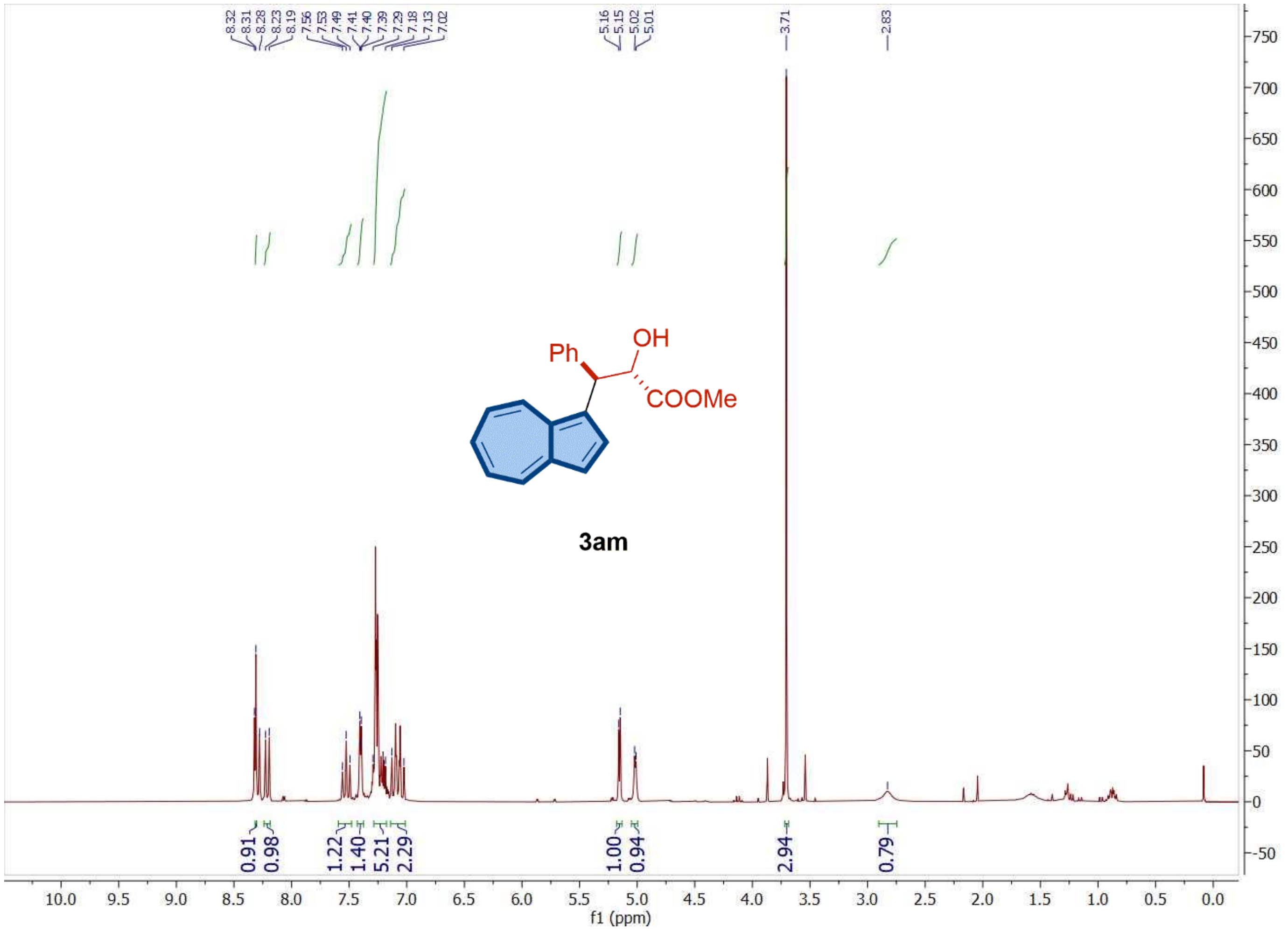

^{13}C NMR of compound 3am (75 MHz, $CDCl_3$)

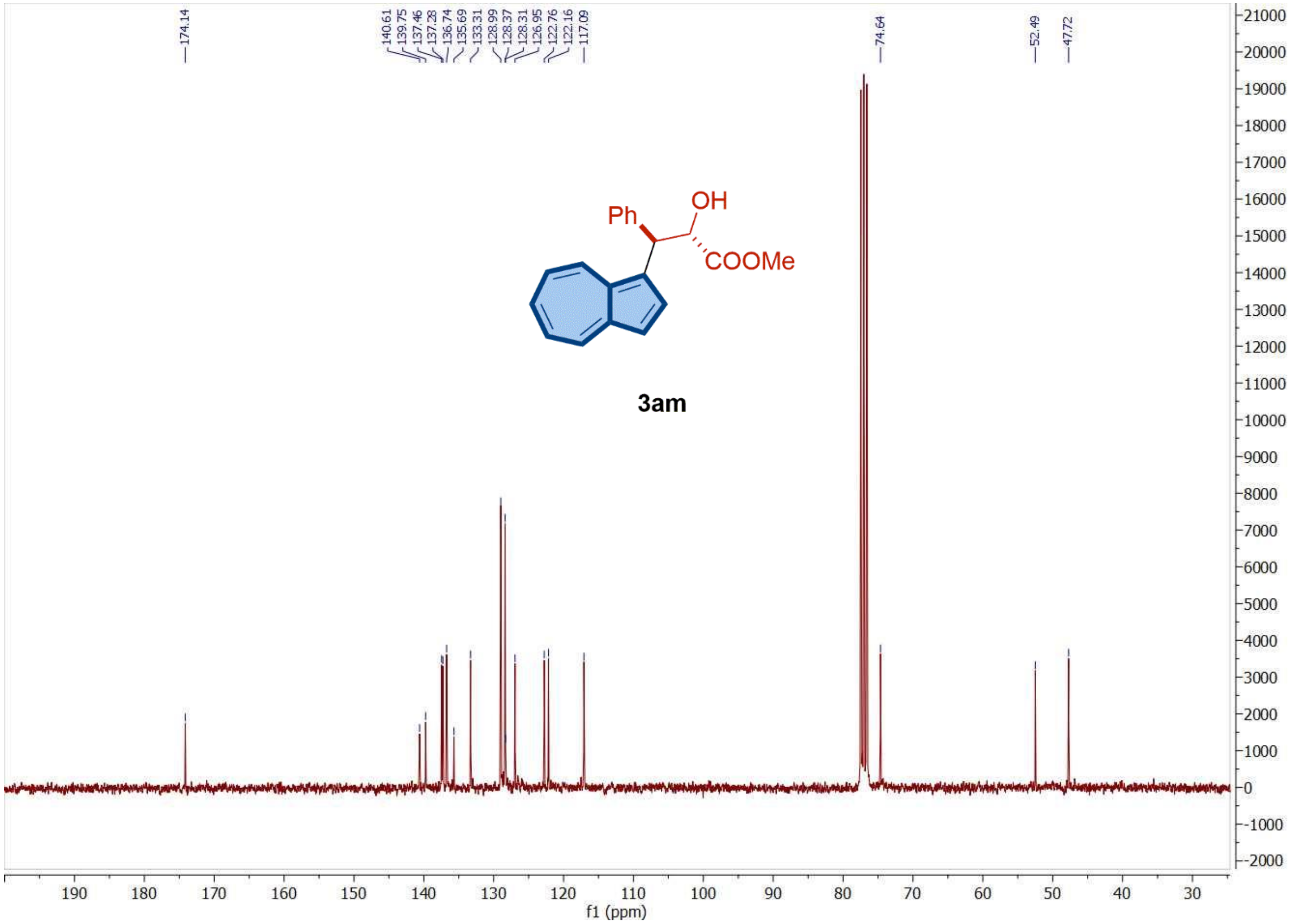

^{1}H NMR of compound 3an (300 MHz, $CDCl_3$)

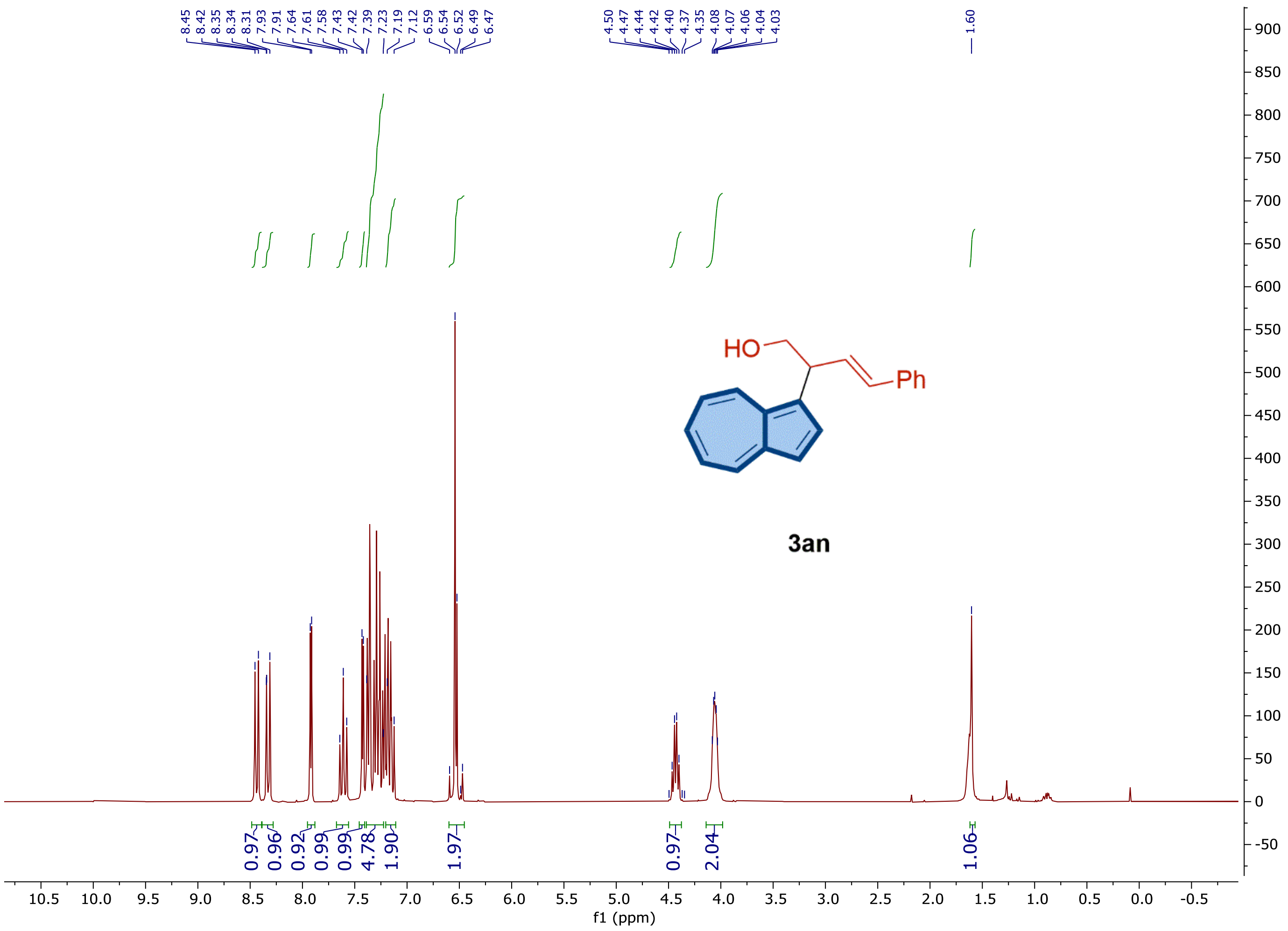

^{13}C NMR of compound 3an (75 MHz, $CDCl_3$)

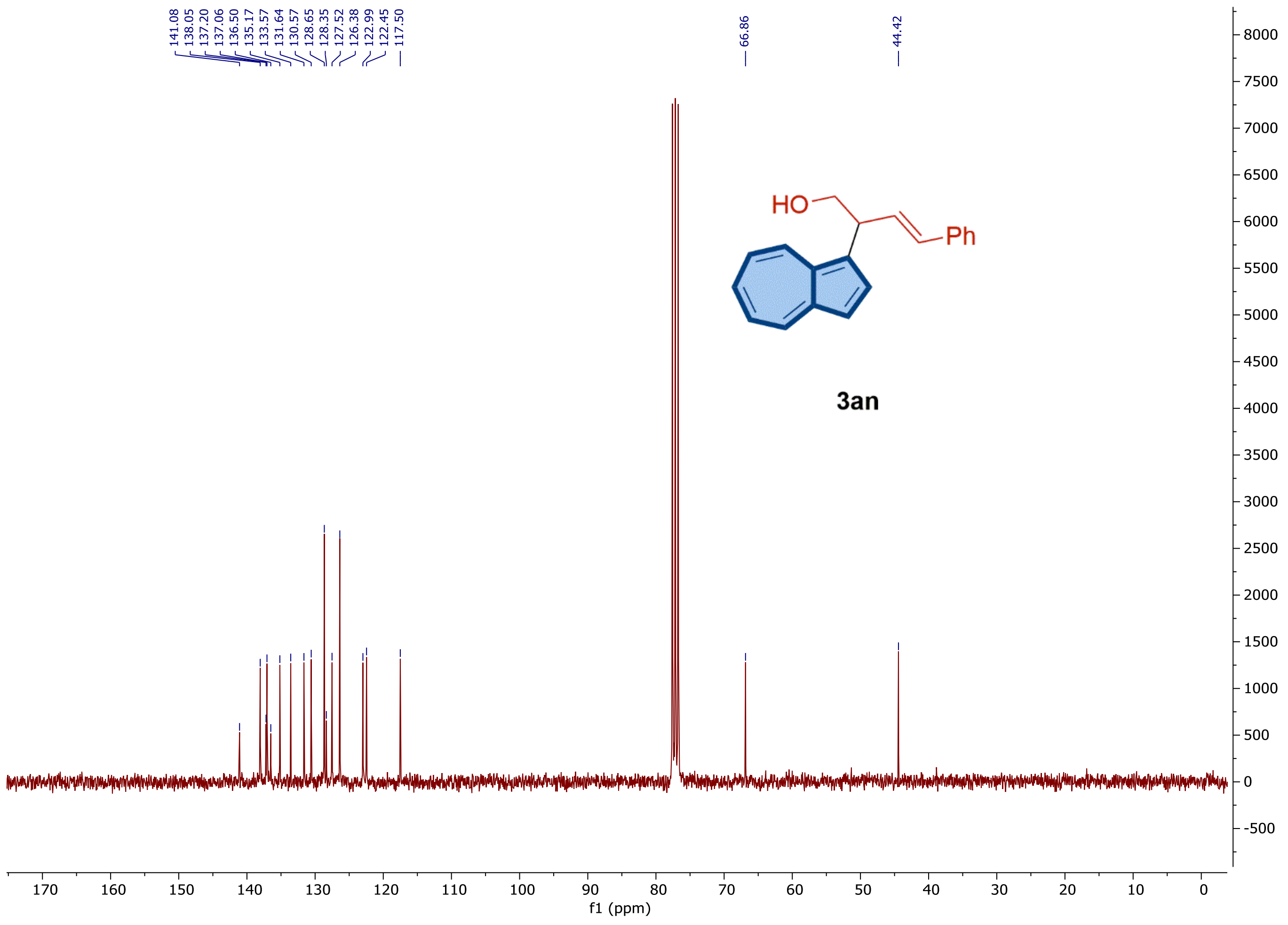

DEPT ^{13}C NMR of compound 3an (75 MHz, $CDCl_3$)

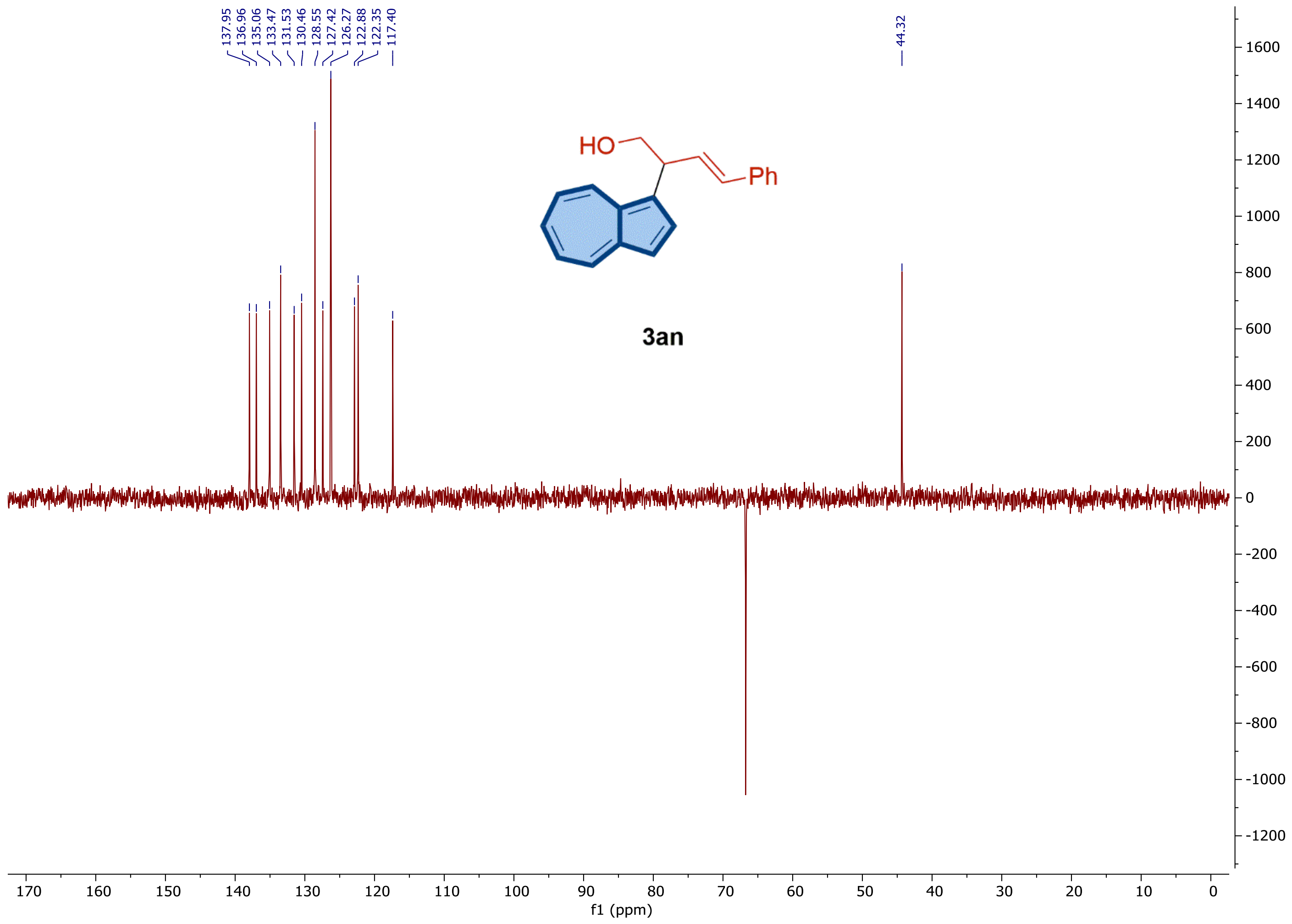

^{1}H NMR of compound 3an' (300 MHz, $CDCl_3$)

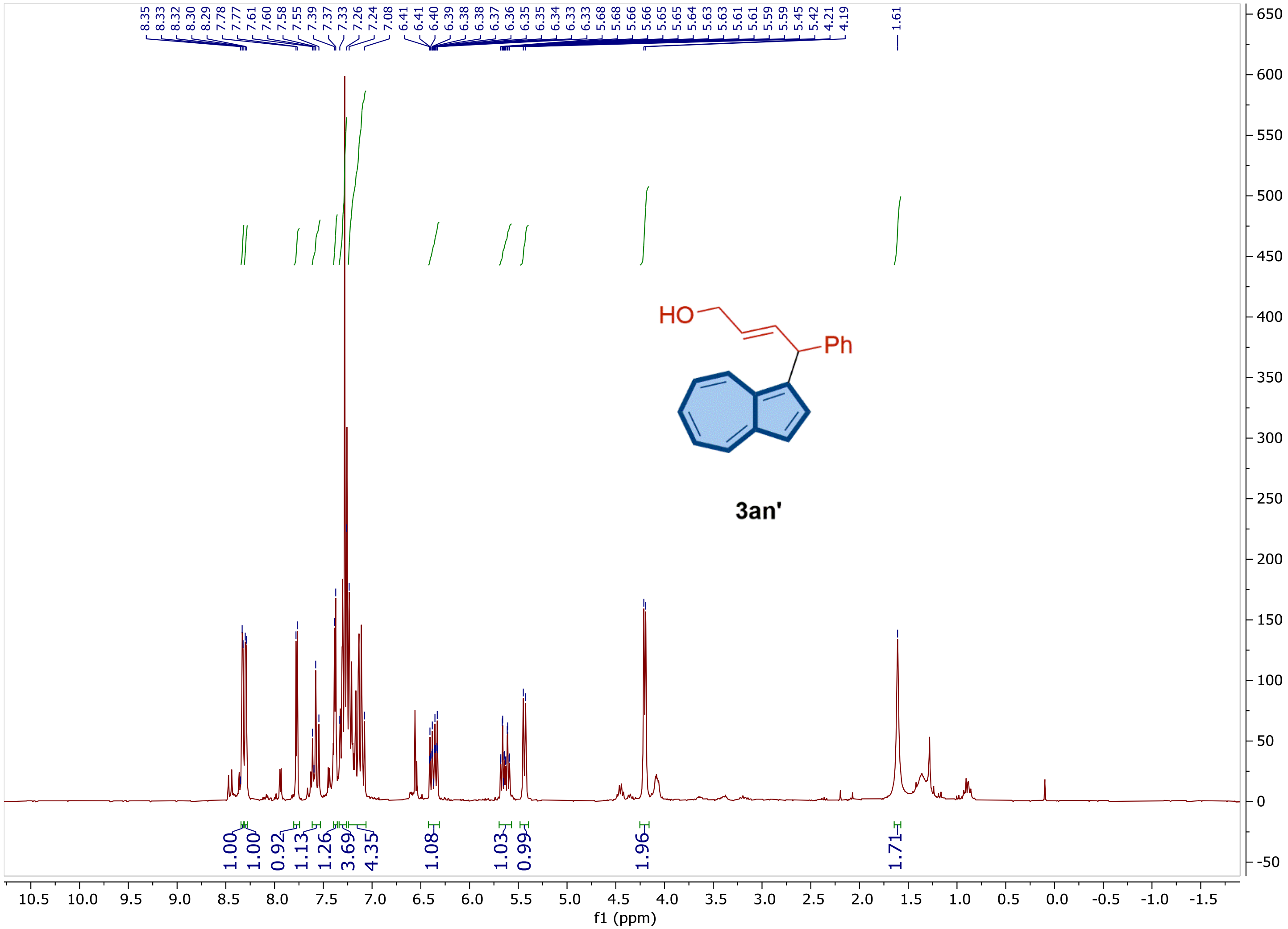

^{13}C NMR of compound 3an' (75 MHz, $CDCl_3$)

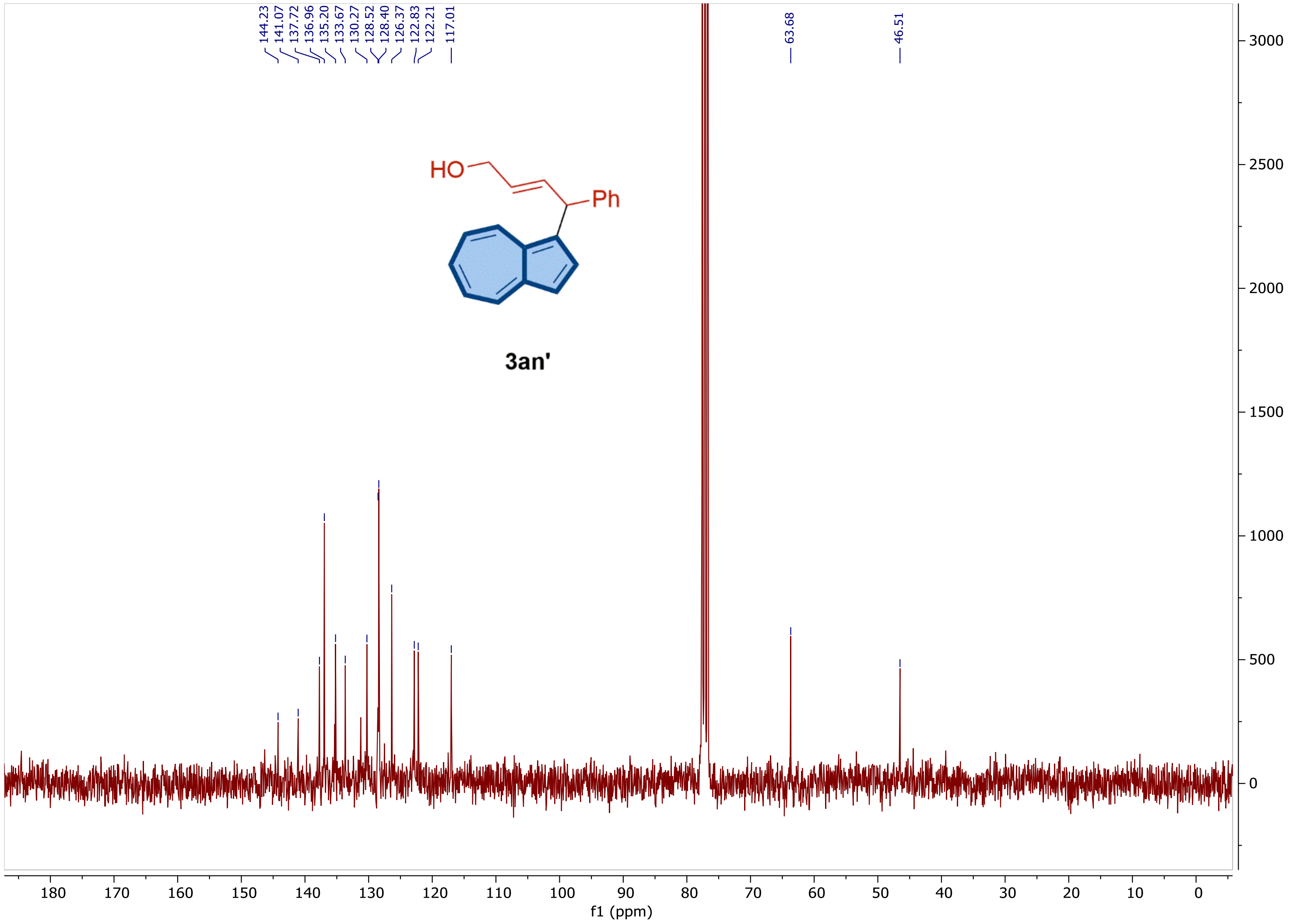

^{1}H NMR of compound 3ao (300 MHz, $CDCl_3$)

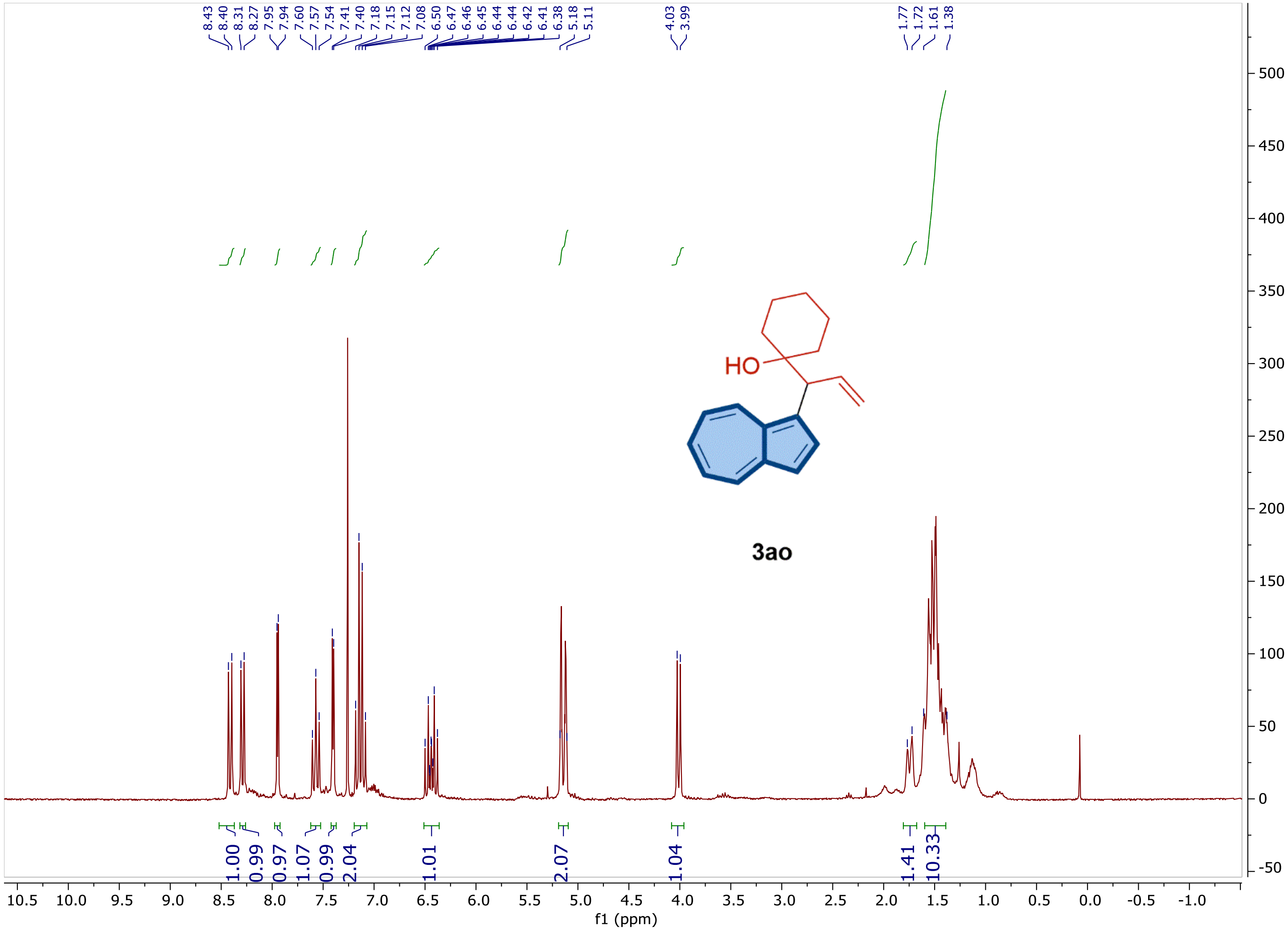

^{13}C NMR of compound 3ao (75 MHz, $CDCl_3$)

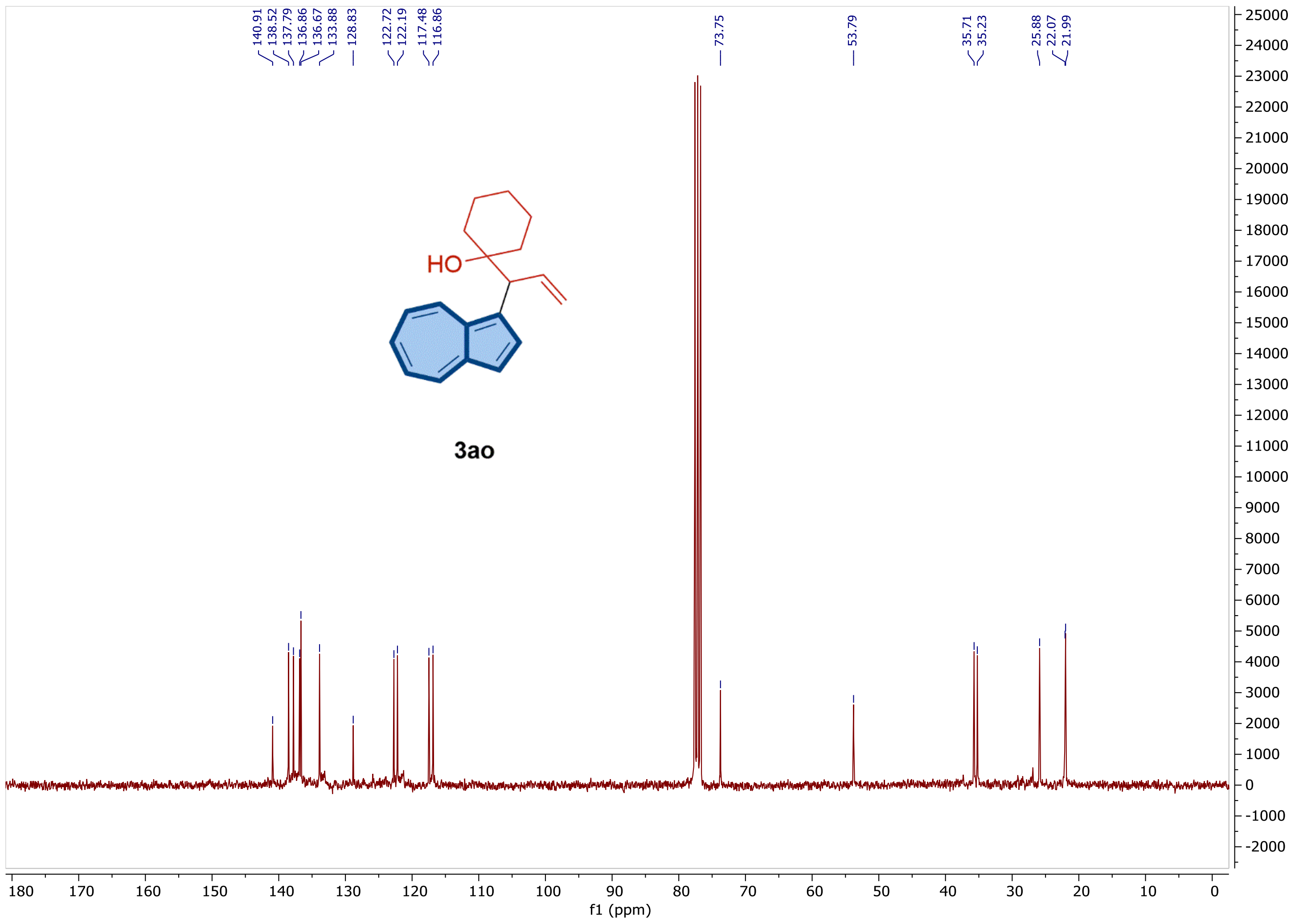

DEPT ^{13}C NMR of compound 3ao (75 MHz, $CDCl_3$)

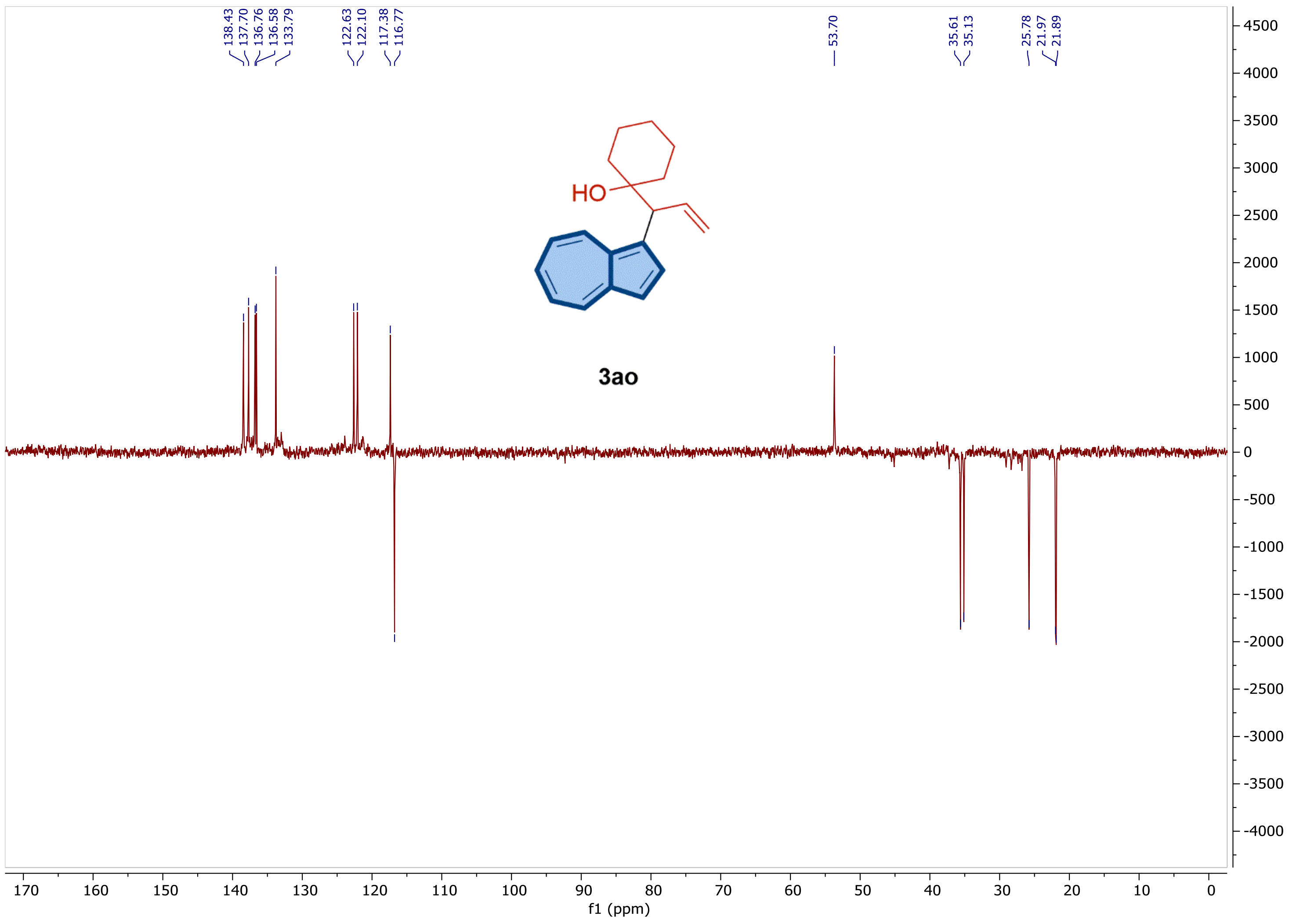

^{1}H NMR of compound 3ap (300 MHz, CDCl3)

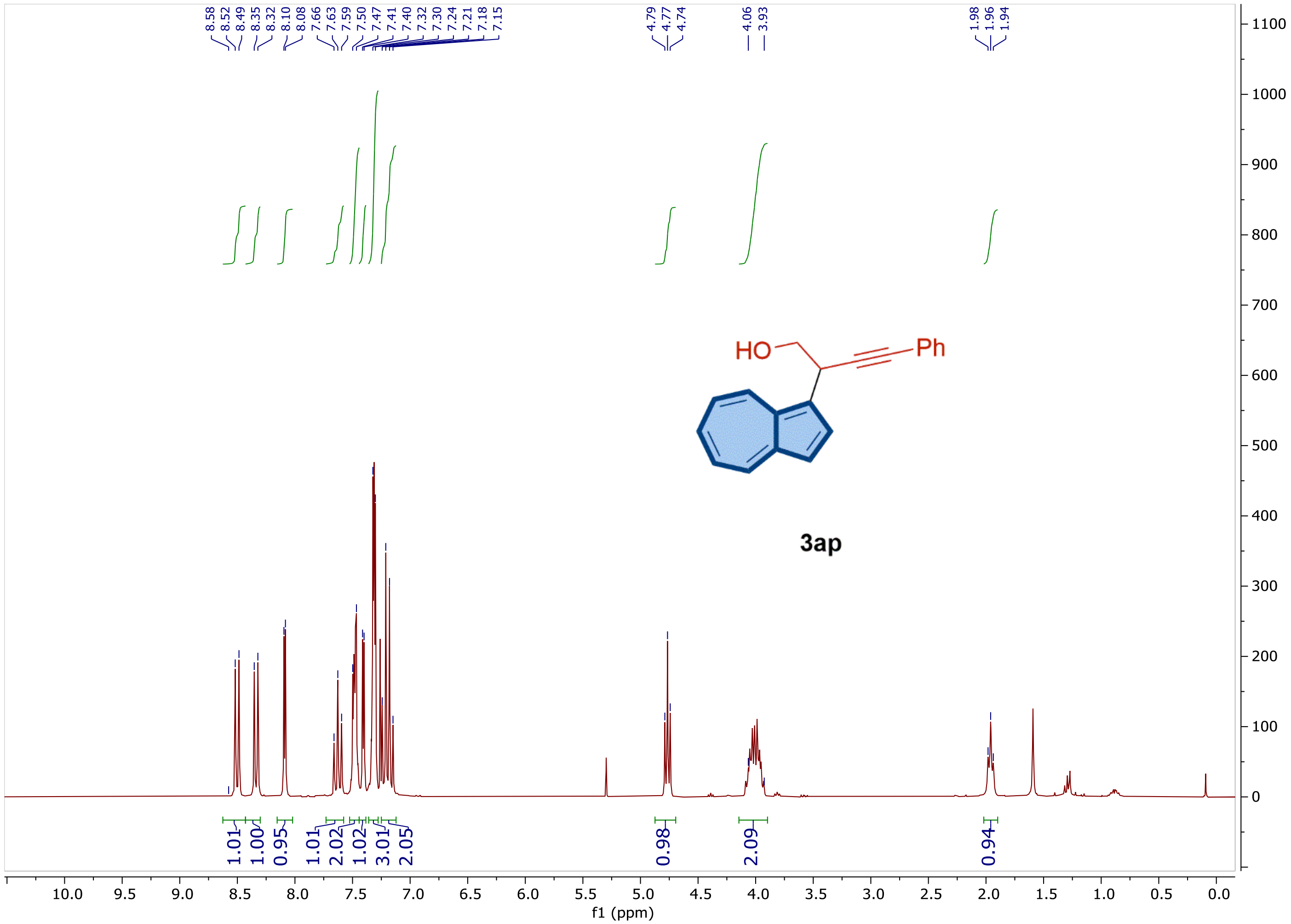

^{13}C NMR of compound 3ap (75 MHz, $CDCl_3$)

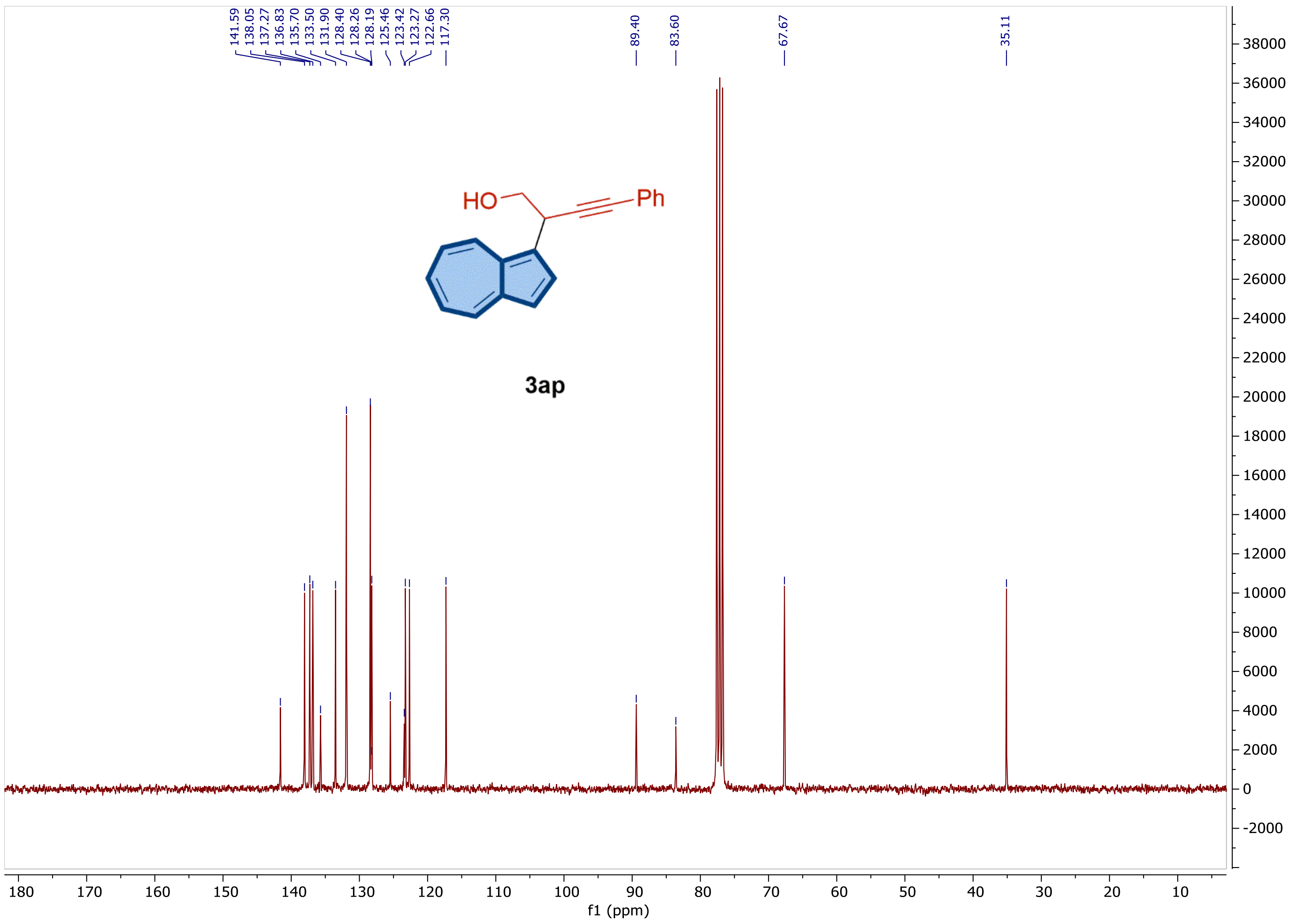

DEPT ^{13}C NMR of compound 3ap (75 MHz, $CDCl_3$)

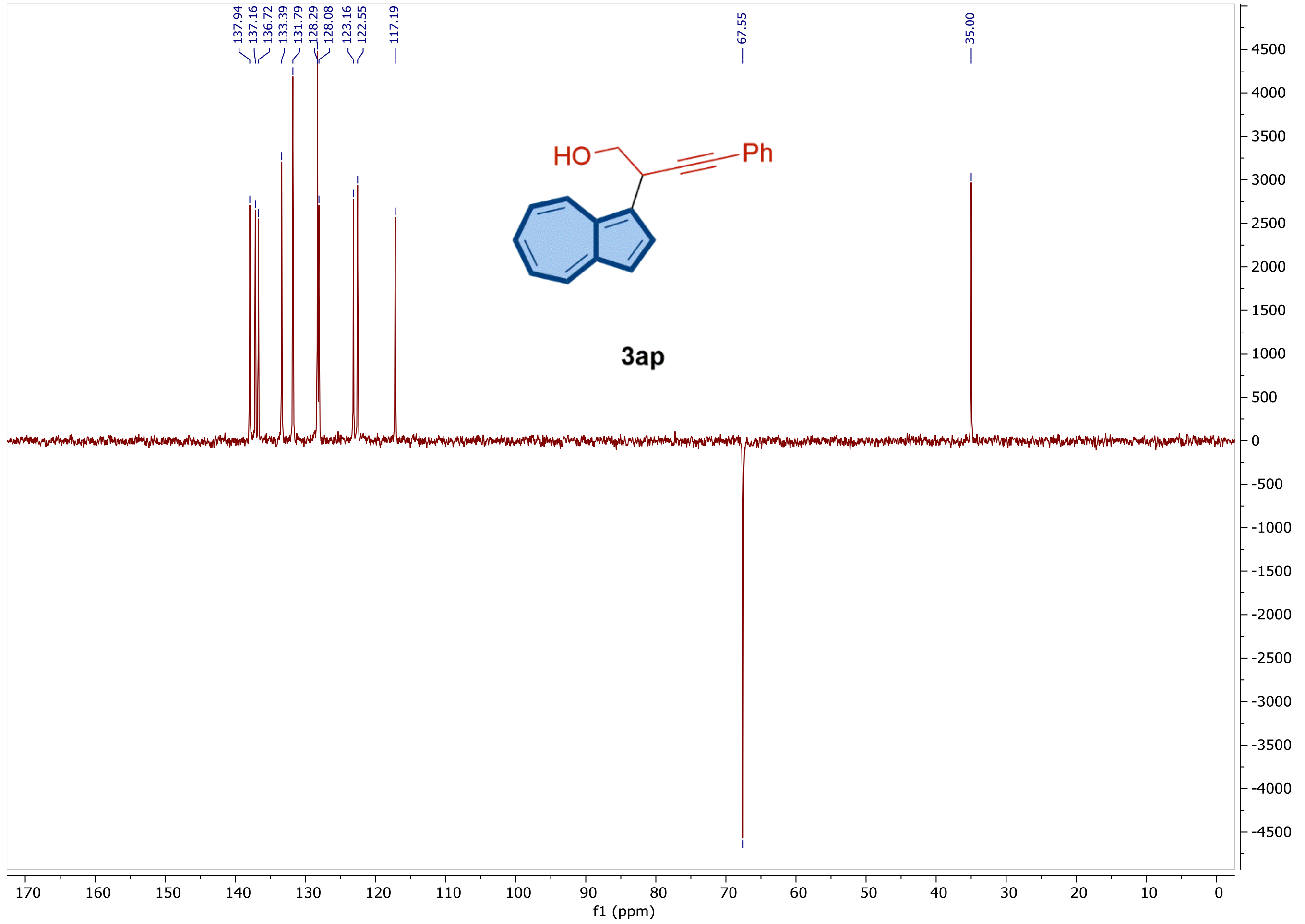

^{1}H NMR of compound 3aq (300 MHz, CDCl$_3$)

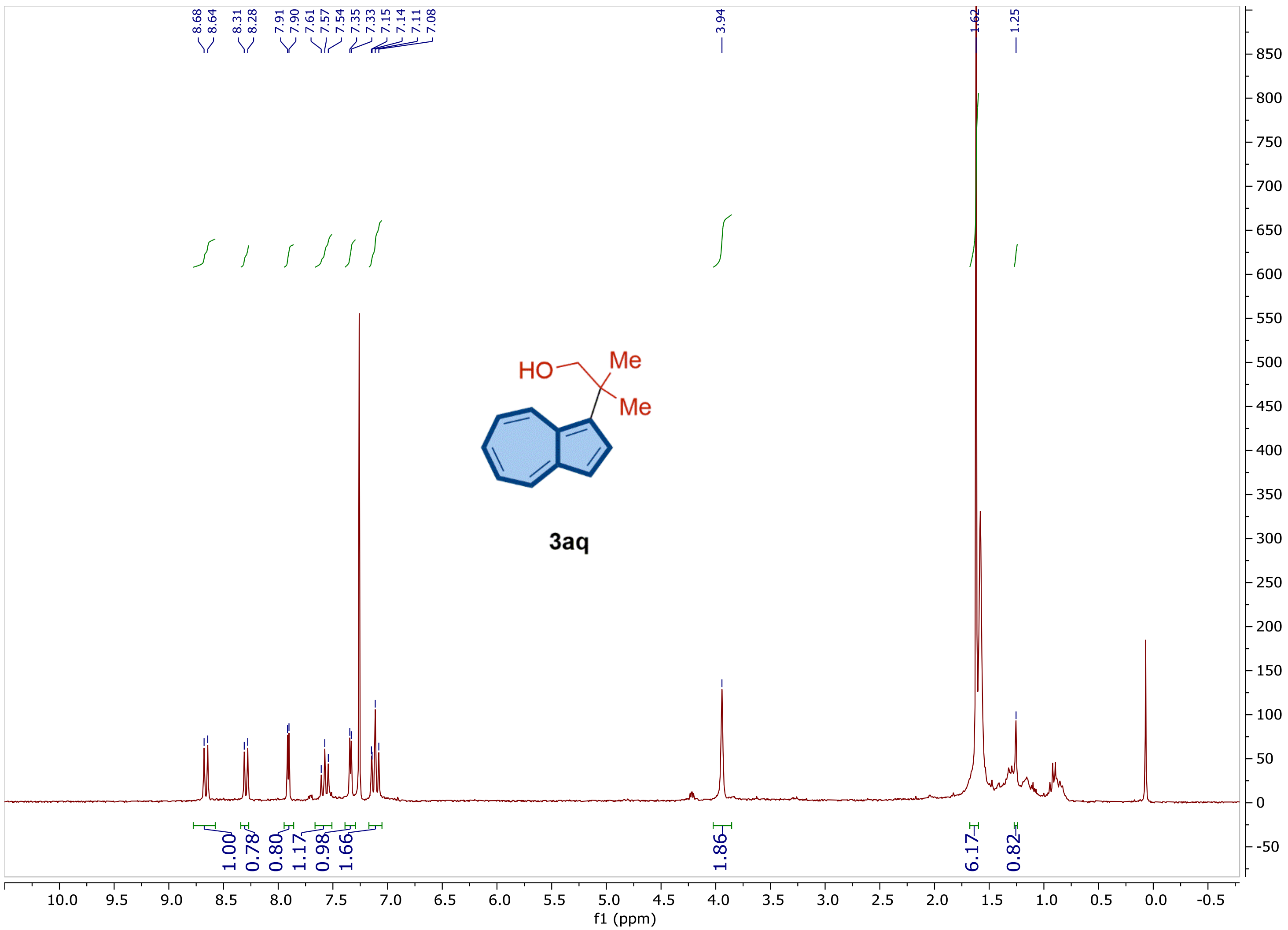

3aq

^{13}C NMR of compound 3aq (75 MHz, $CDCl_3$)

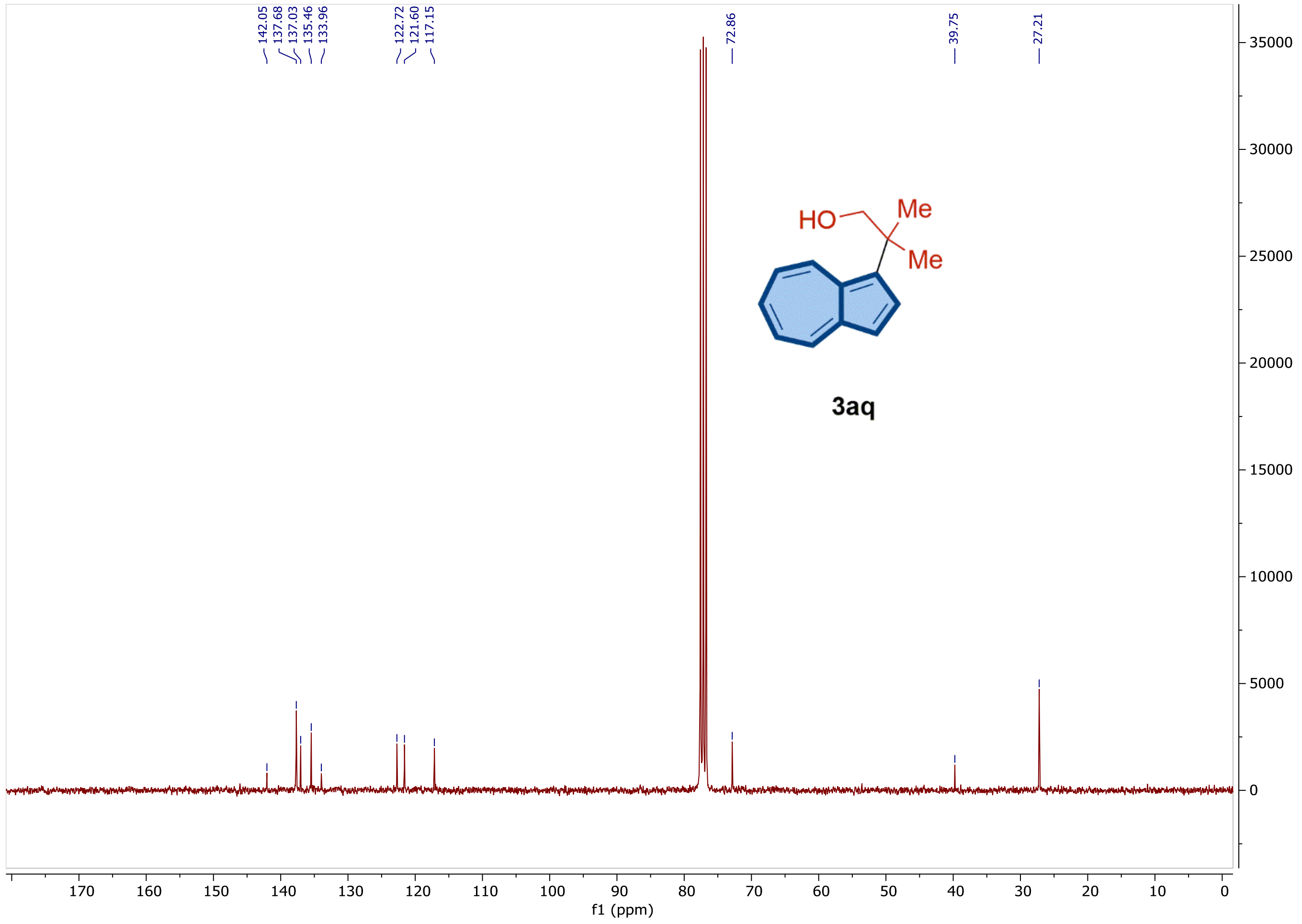

DEPT ^{13}C NMR of compound 3aq (75 MHz, $CDCl_3$)

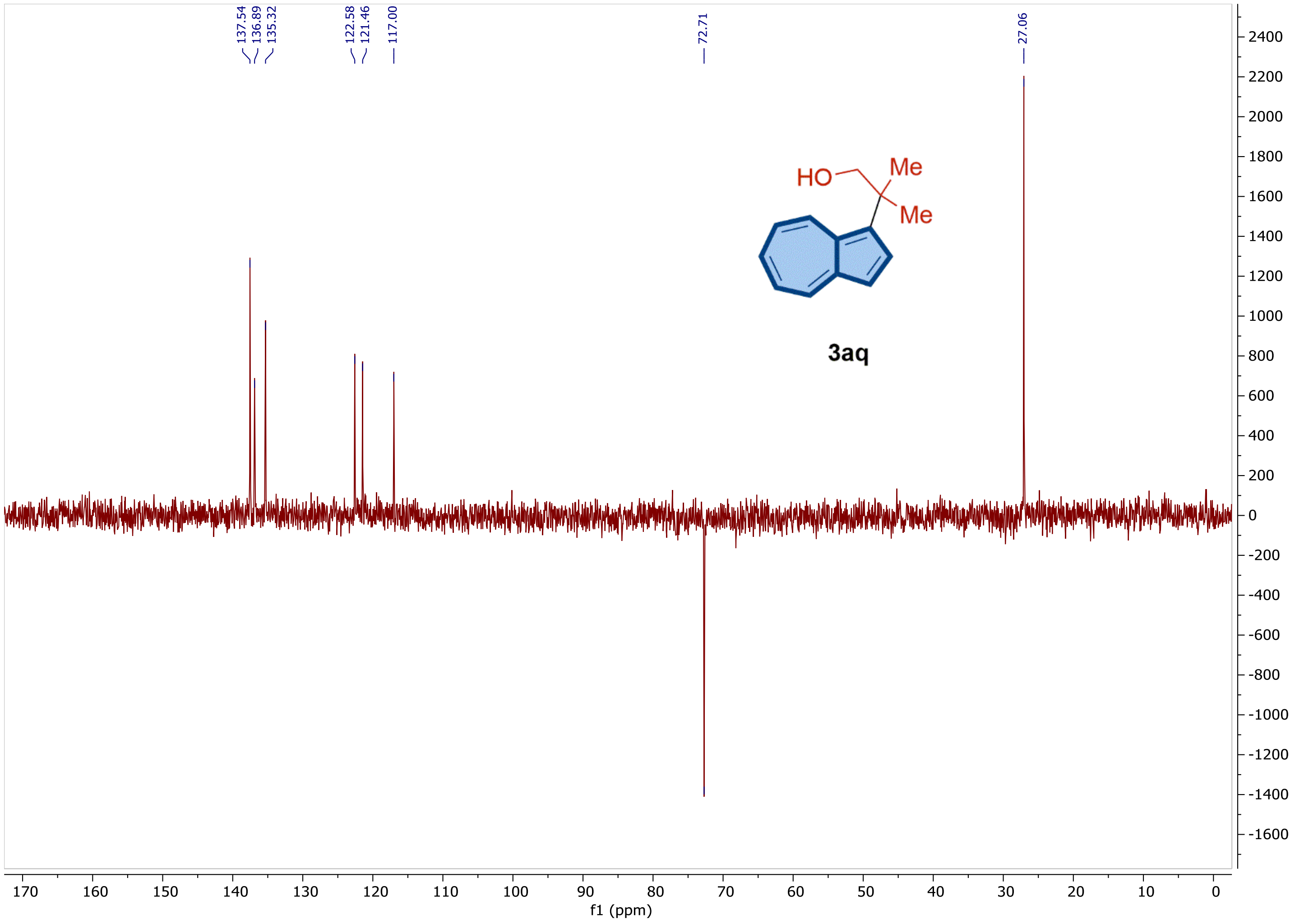

^{1}H NMR of an inseparable 3:1 mixture of compounds 3ar + 3ar' (300 MHz, $CDCl_3$)

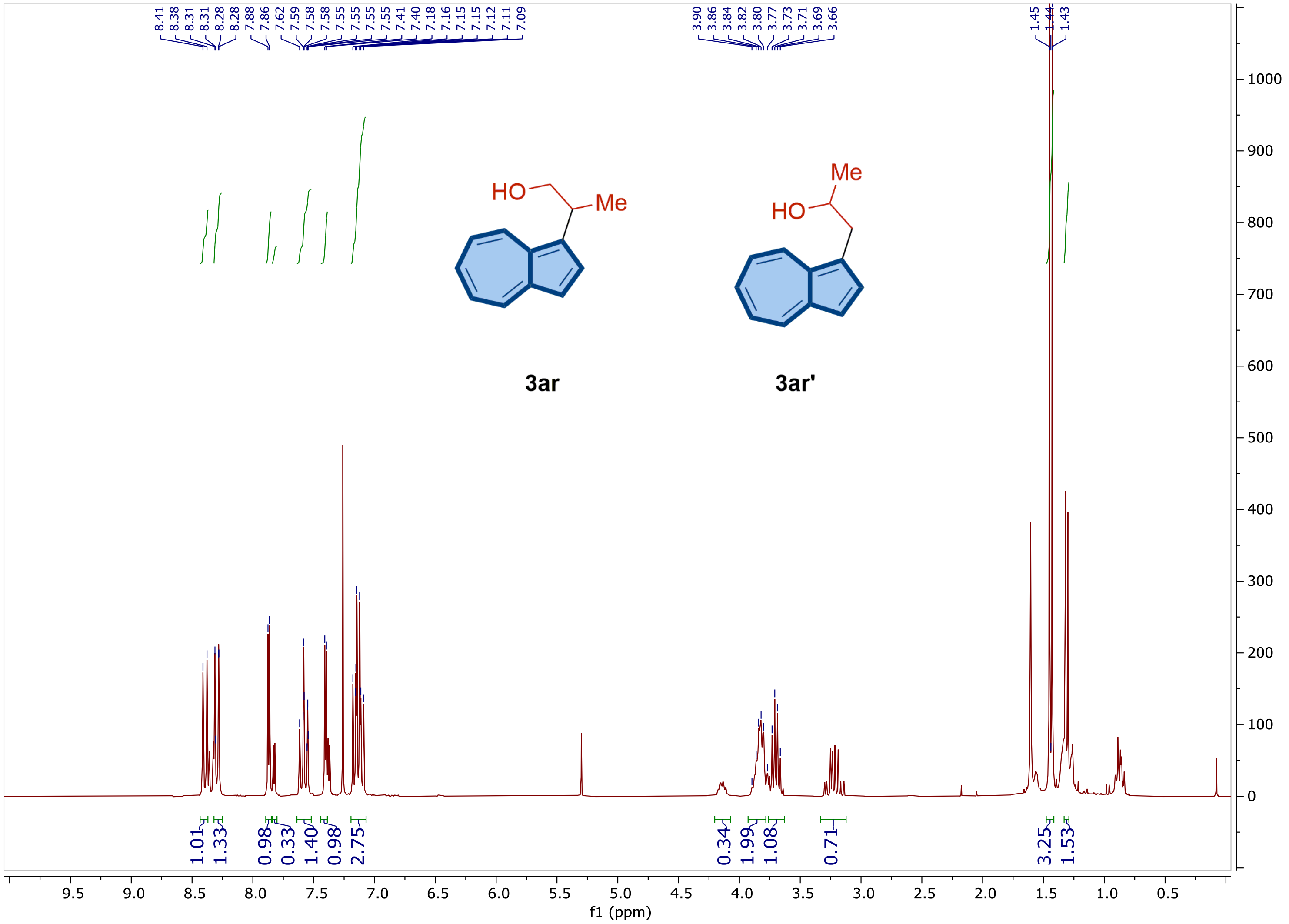

^{13}C NMR of an inseparable 3:1 mixture of compounds 3ar + 3ar' (75 MHz, $CDCl_3$)

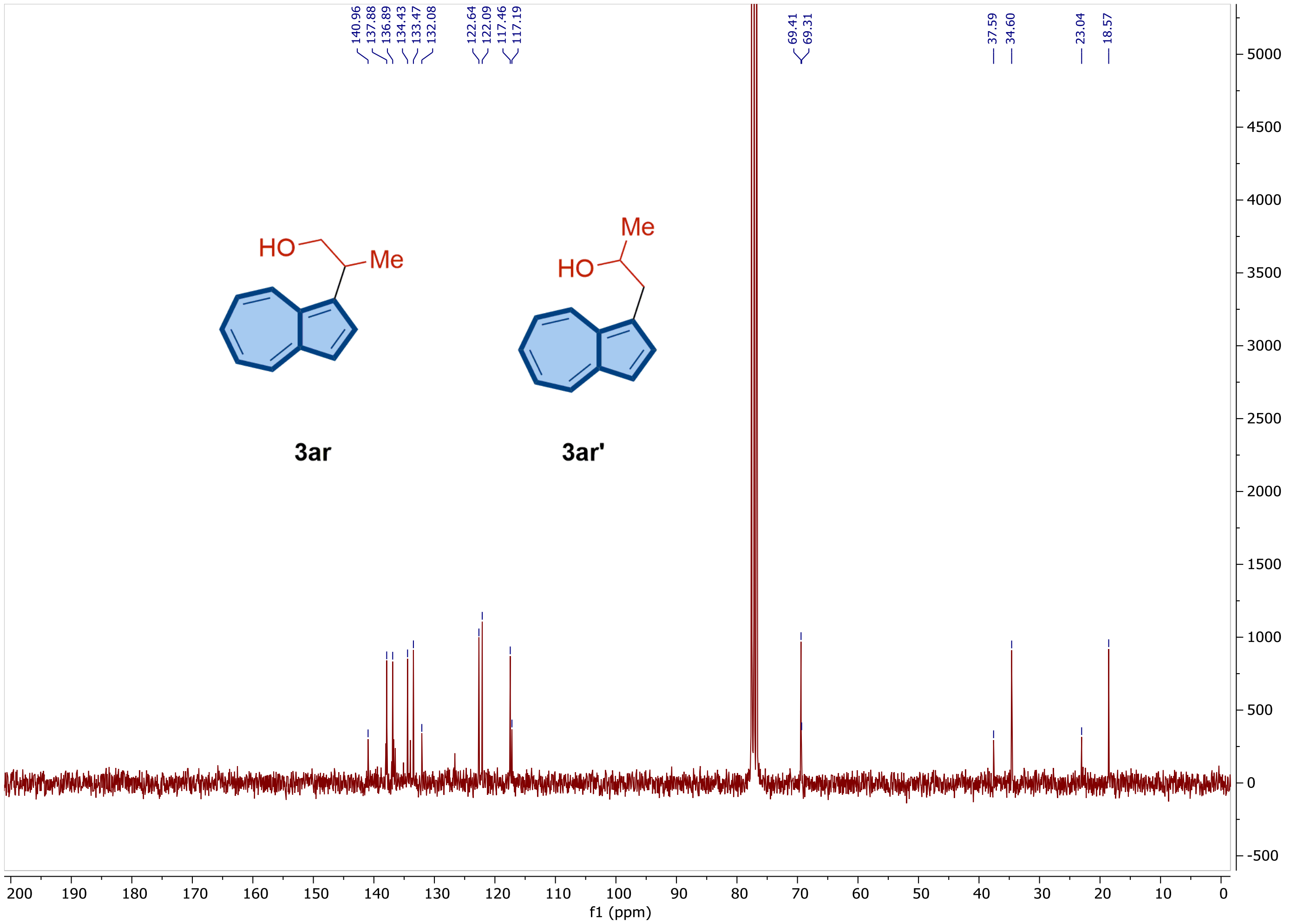

DEPT ^{13}C NMR of an inseparable 3:1 mixture of compounds 3ar + 3ar’ (75 MHz, $CDCl_3$)

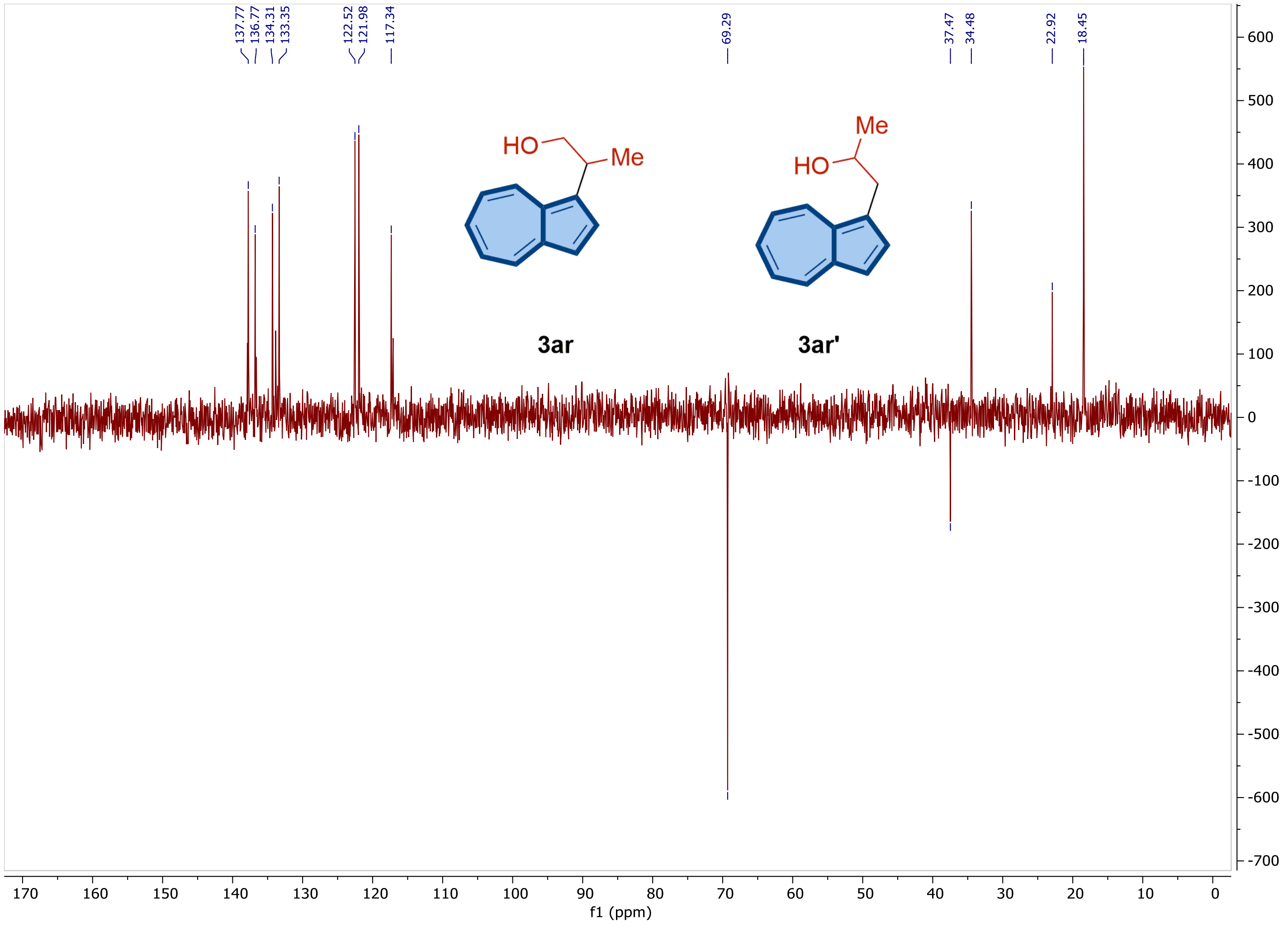

^{1}H NMR of compound 3as' (300 MHz, $CDCl_3$)

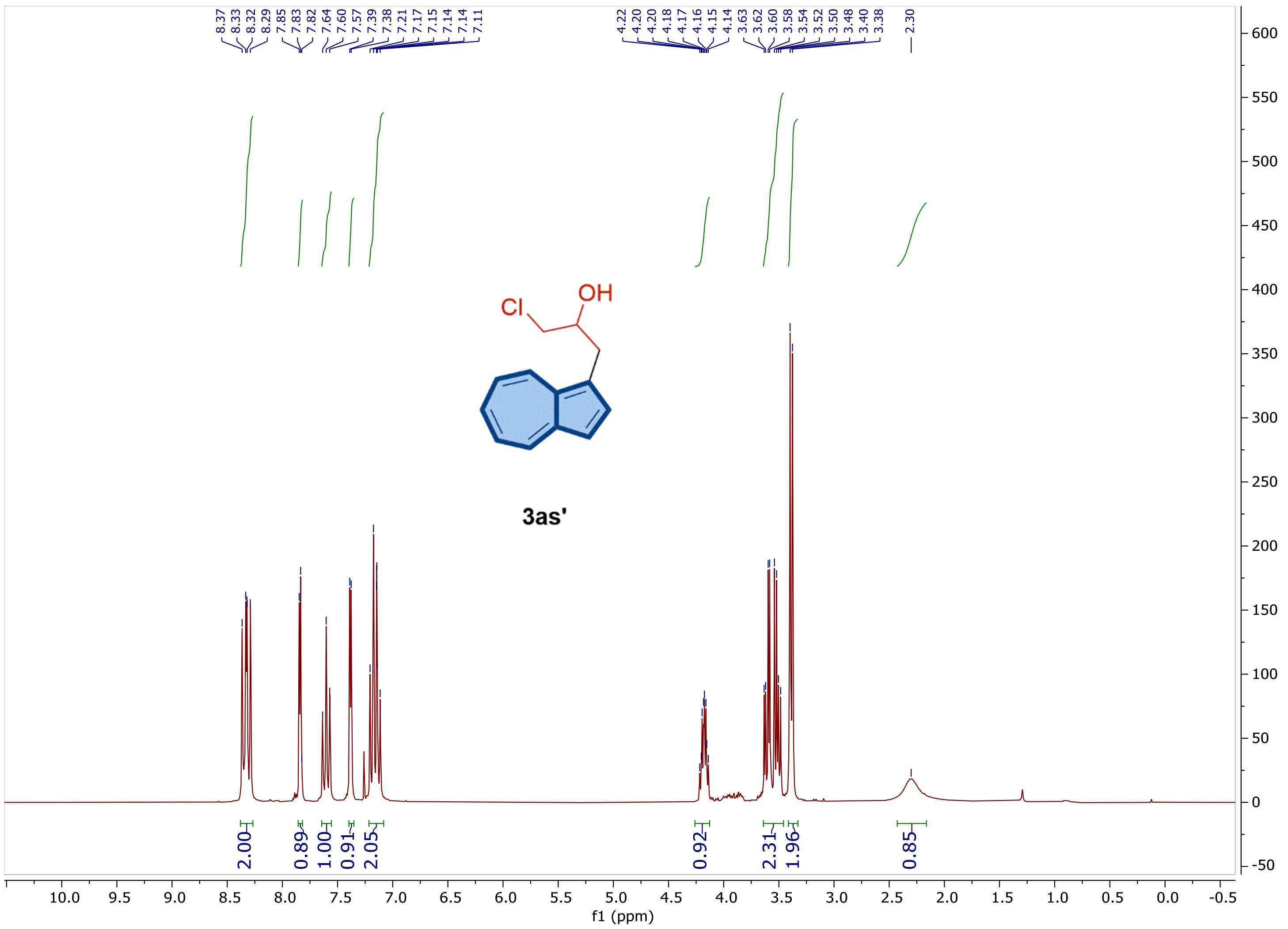

^{13}C NMR of compound 3as' (75 MHz, $CDCl_3$)

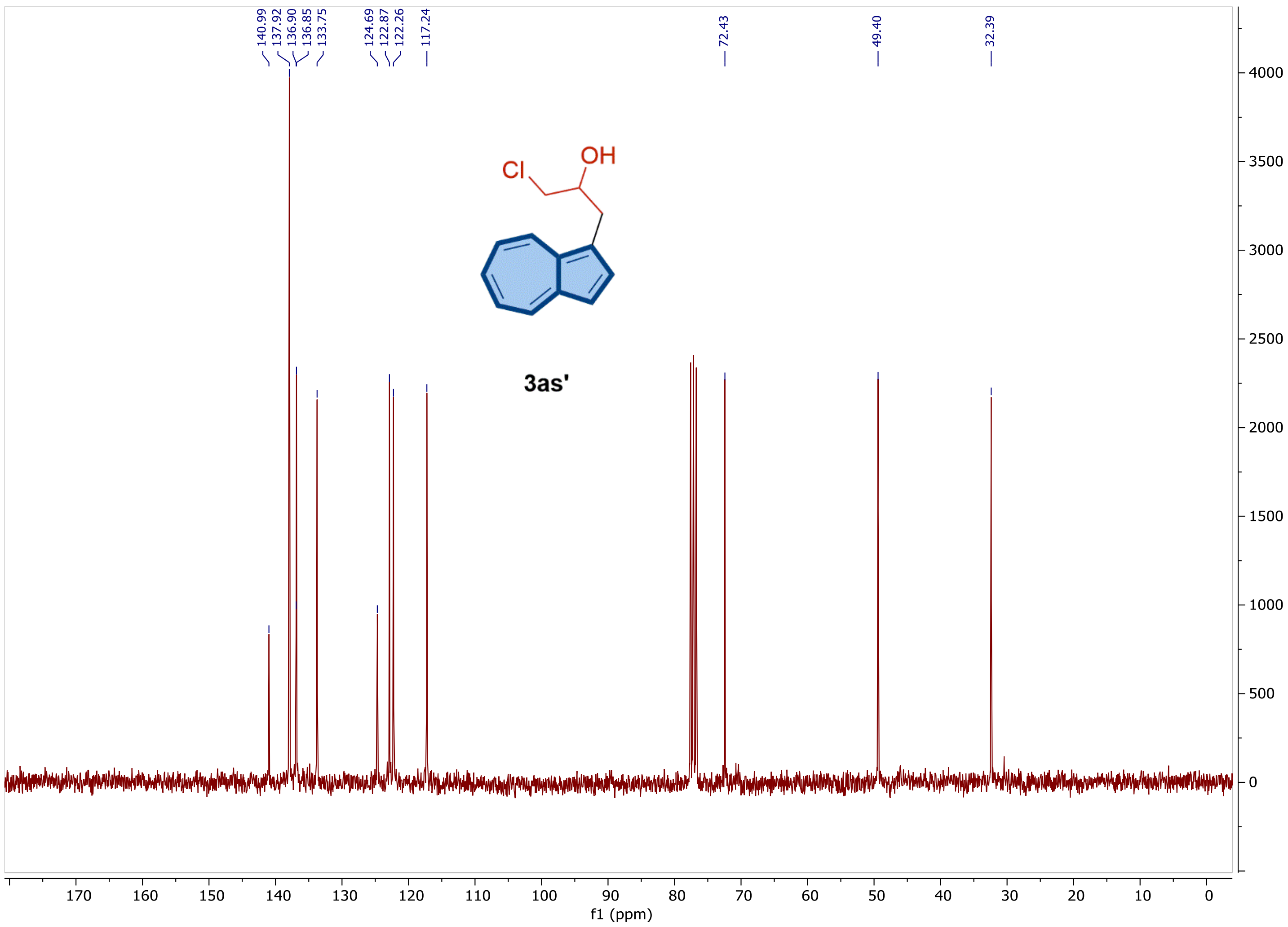

DEPT ^{13}C NMR of compound 3as’ (75 MHz, $CDCl_3$)

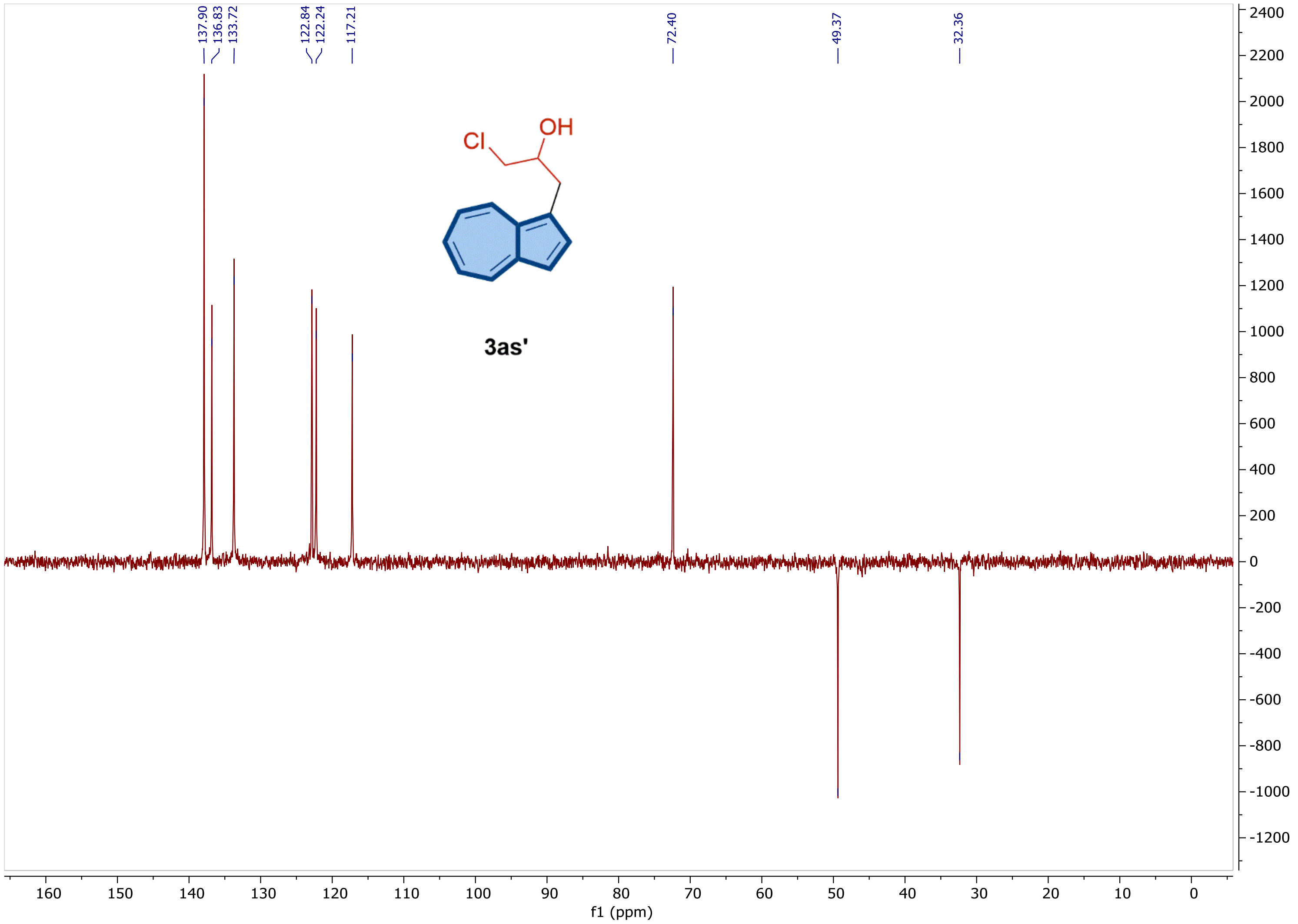

^{1}H NMR of compound 3at' (300 MHz, $CDCl_3$)

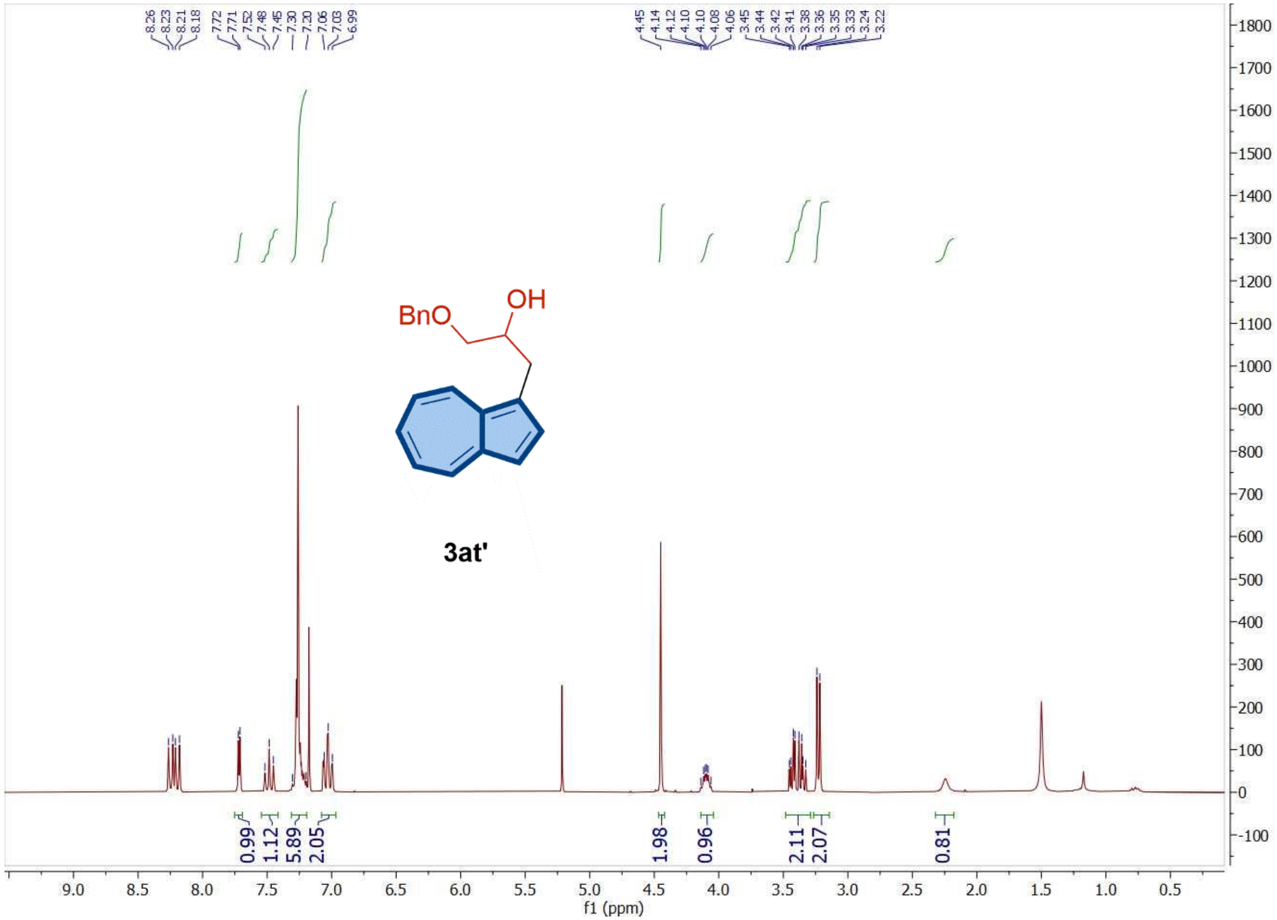

^{13}C NMR of compound 3at' (75 MHz, $CDCl_3$)

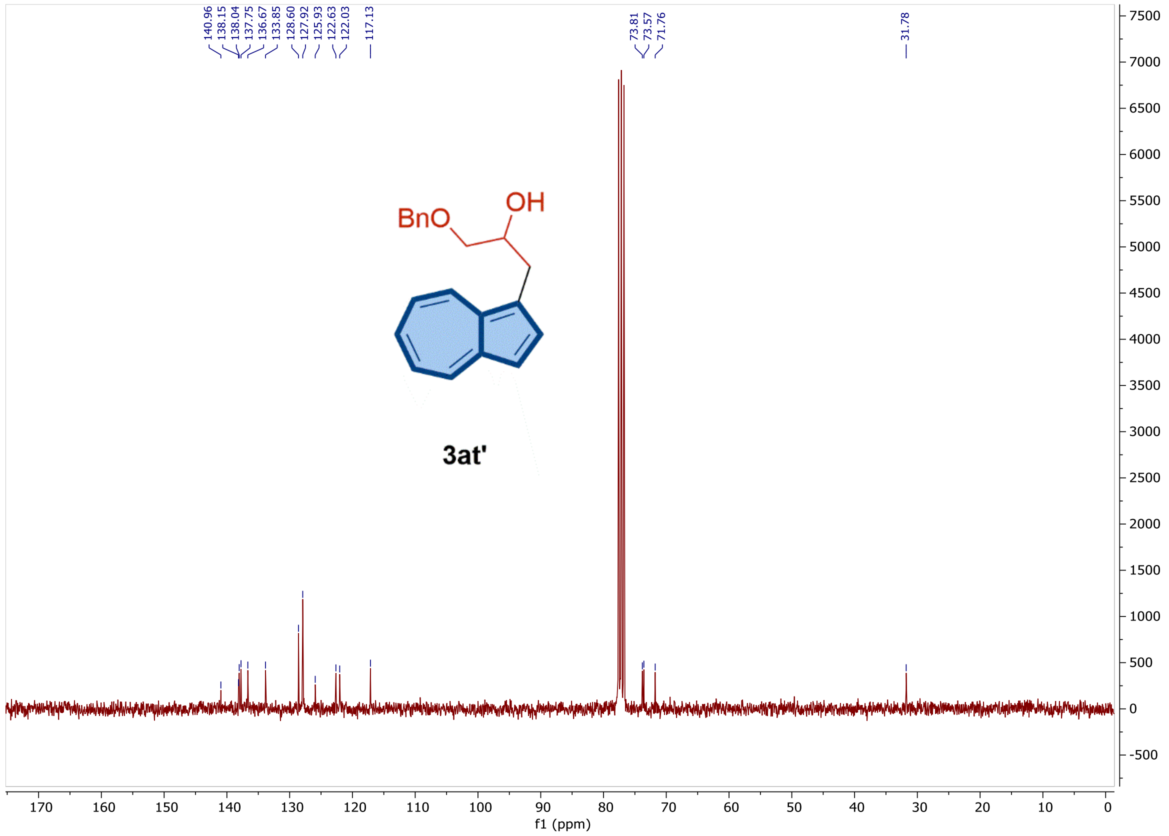

^{1}H NMR of compound 3ba (300 MHz, $CDCl_3$)

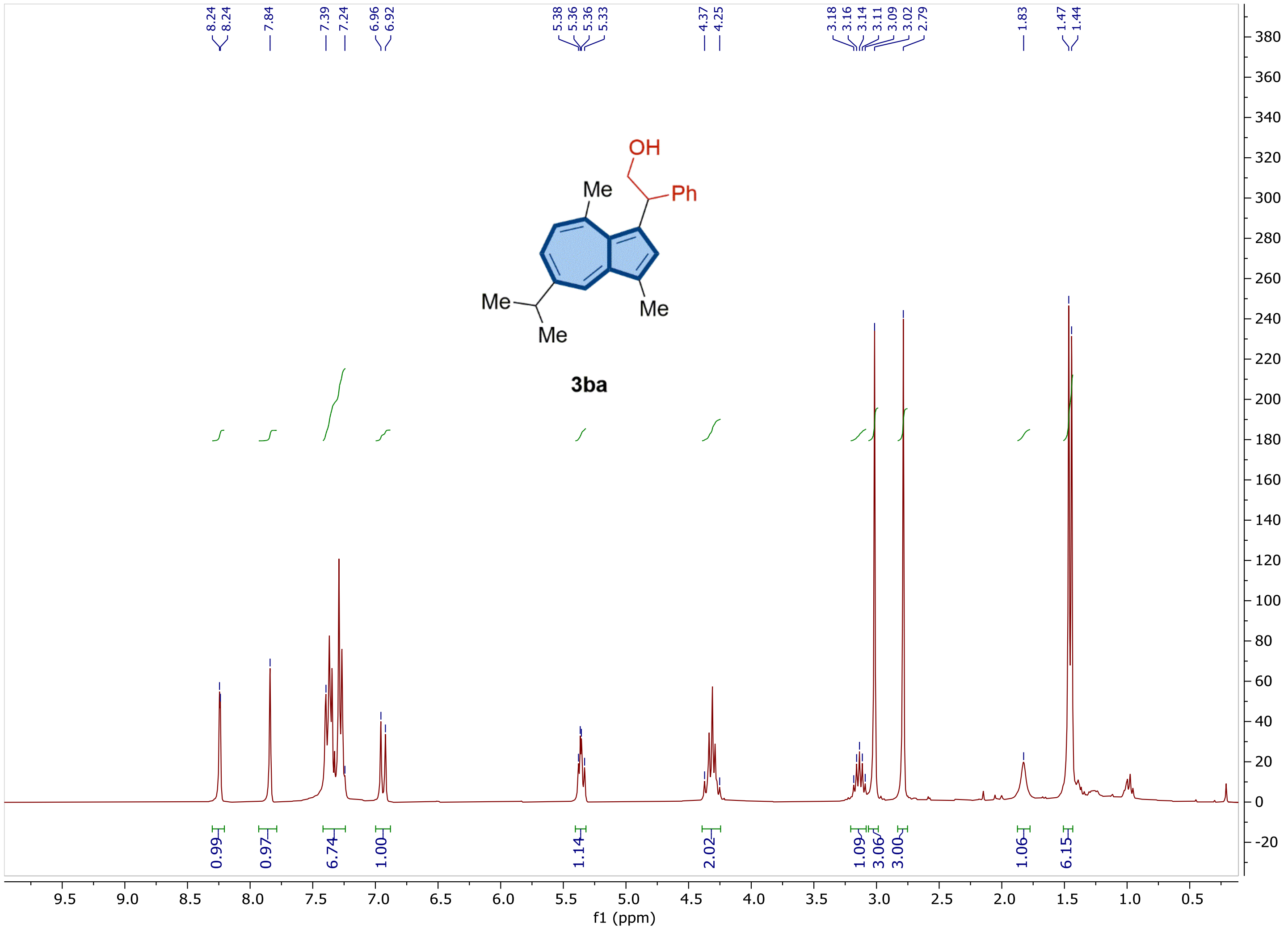

^{13}C NMR of compound 3ba (75 MHz, $CDCl_3$)

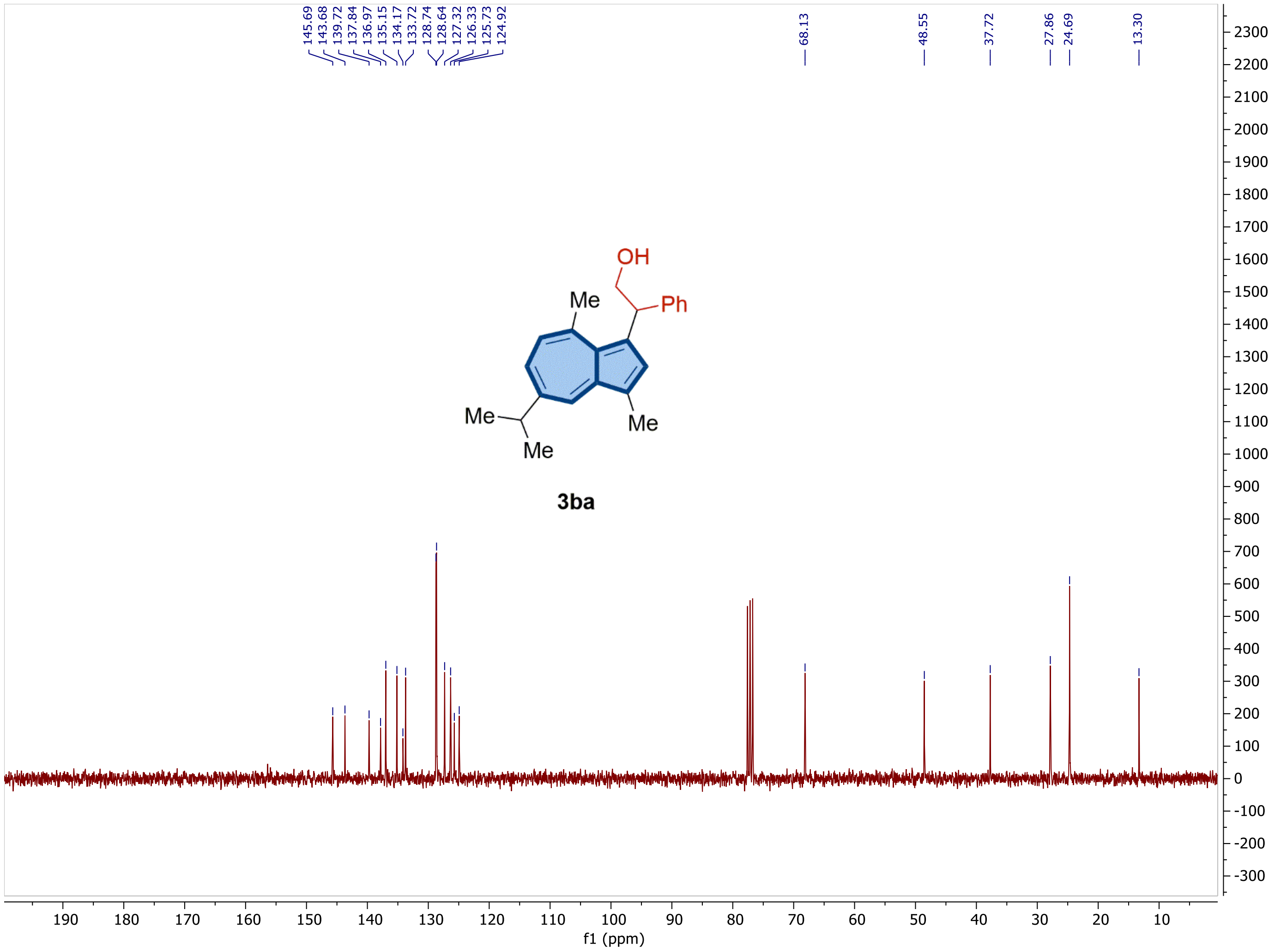

DEPT ^{13}C NMR of compound 3ba (75 MHz, $CDCl_3$)

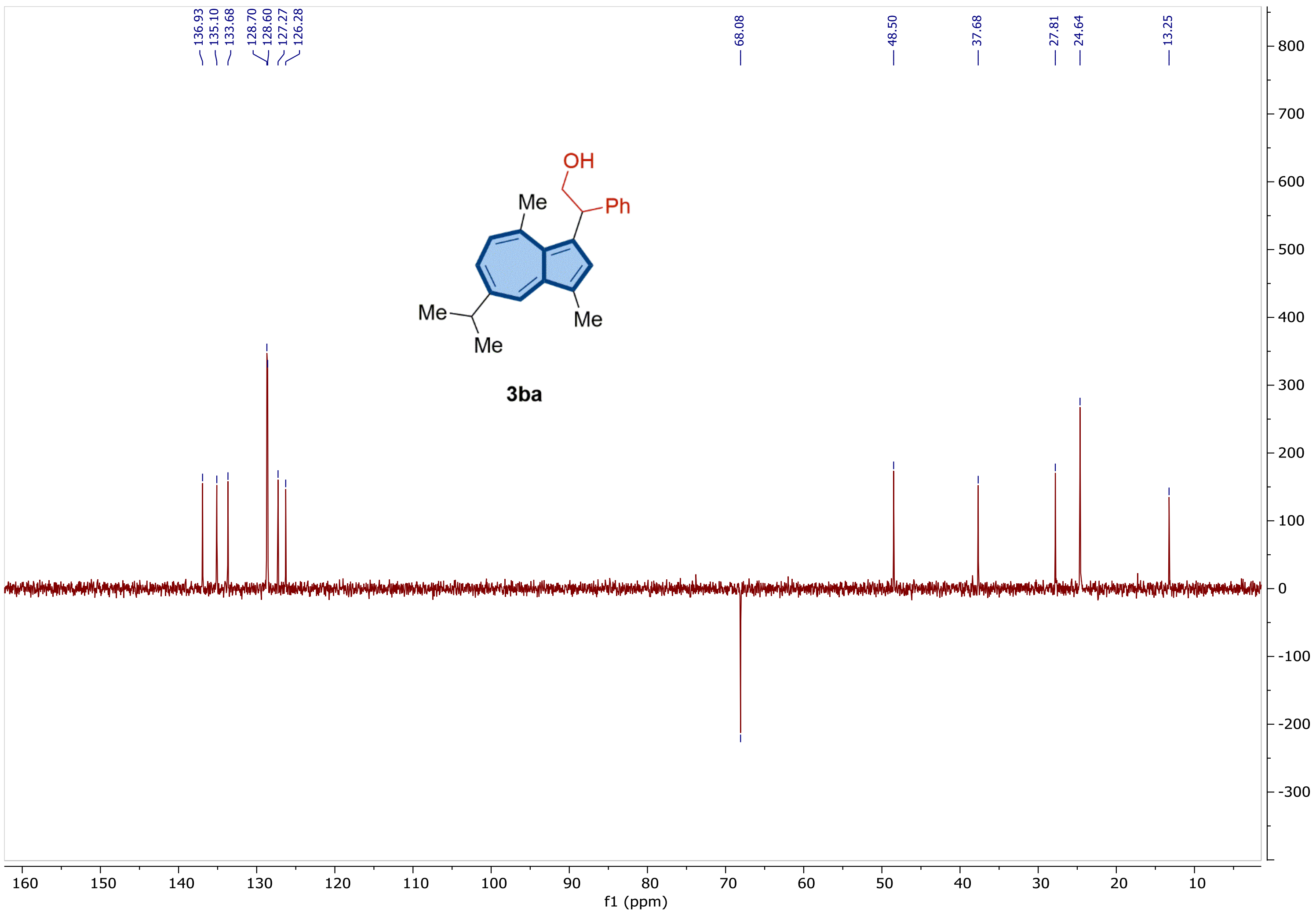

^{1}H NMR of compound 3bb (300 MHz, $CDCl_3$)

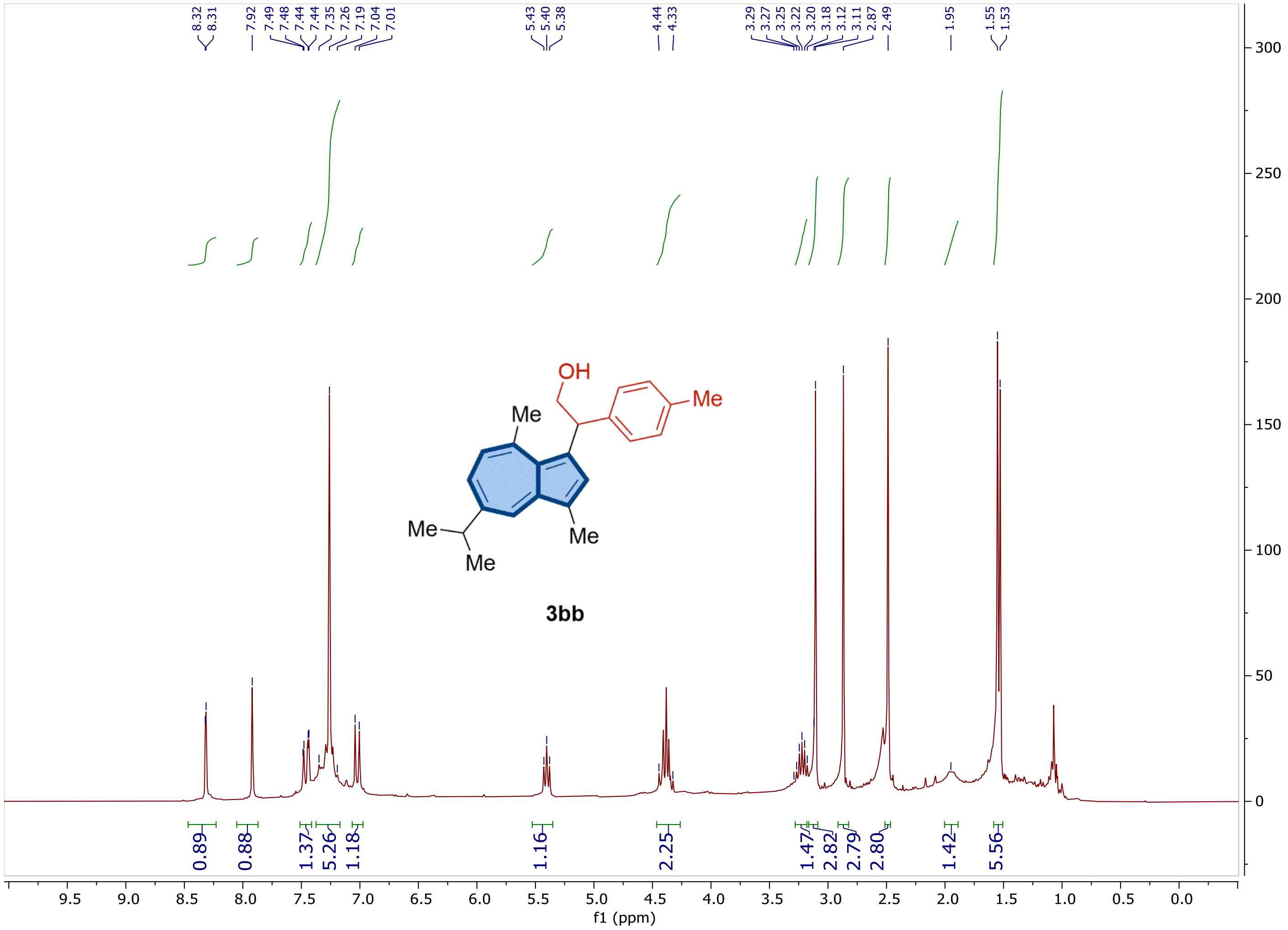

^{13}C NMR of compound 3bb (75 MHz, $CDCl_3$)

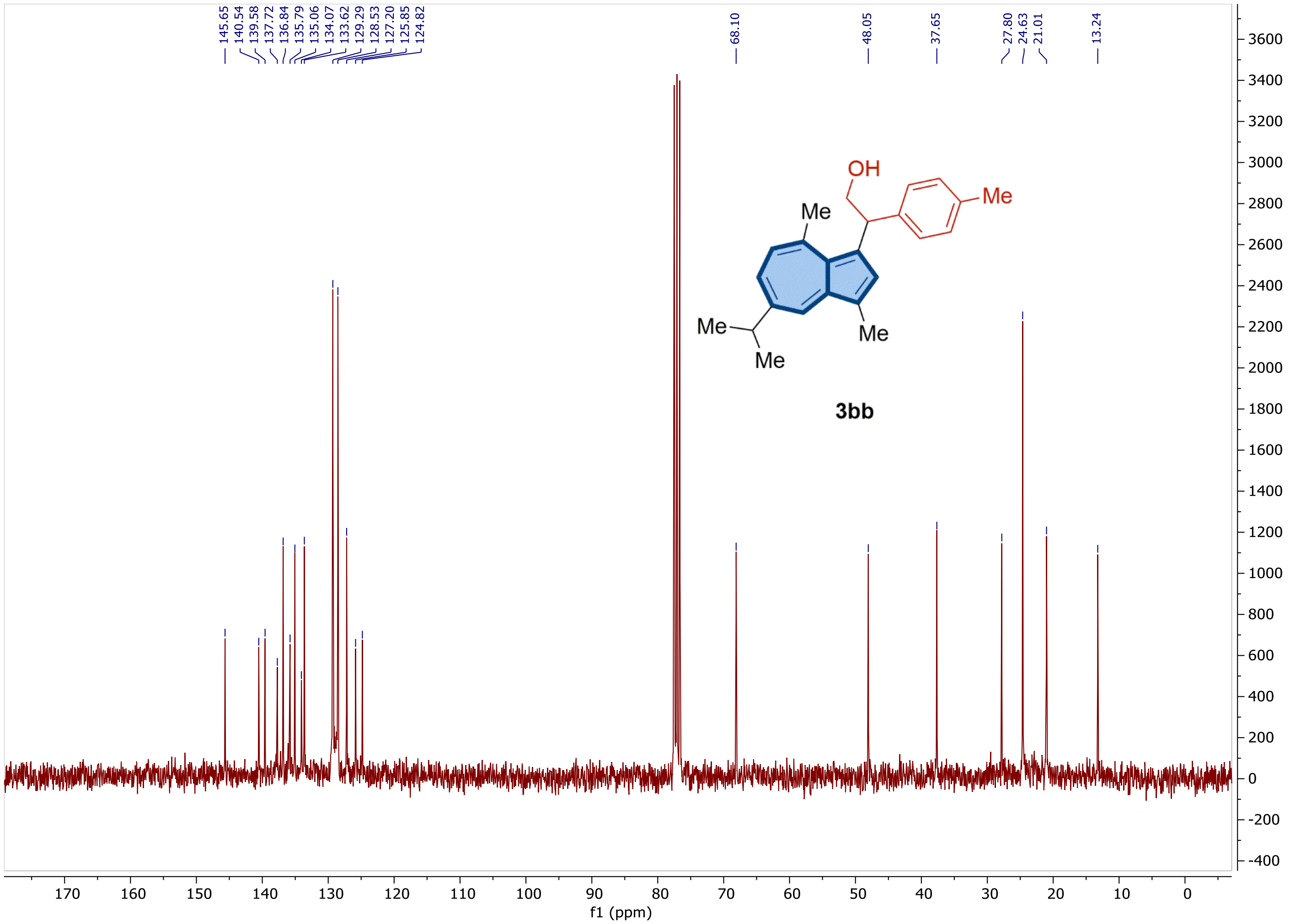

DEPT ^{13}C NMR of compound 3bb (75 MHz, $CDCl_3$)

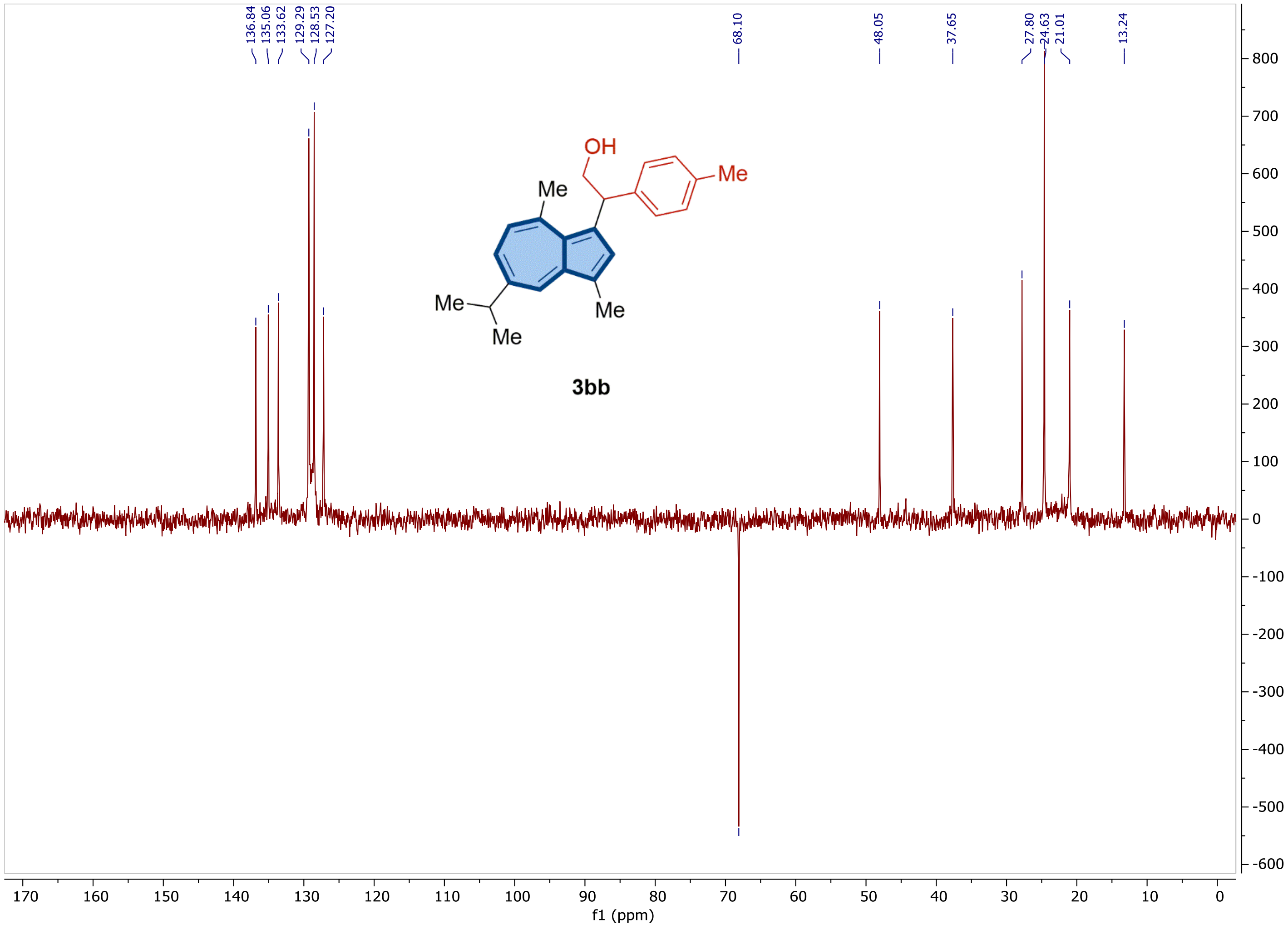

^{1}H NMR of compound 3bd (300 MHz, $CDCl_3$)

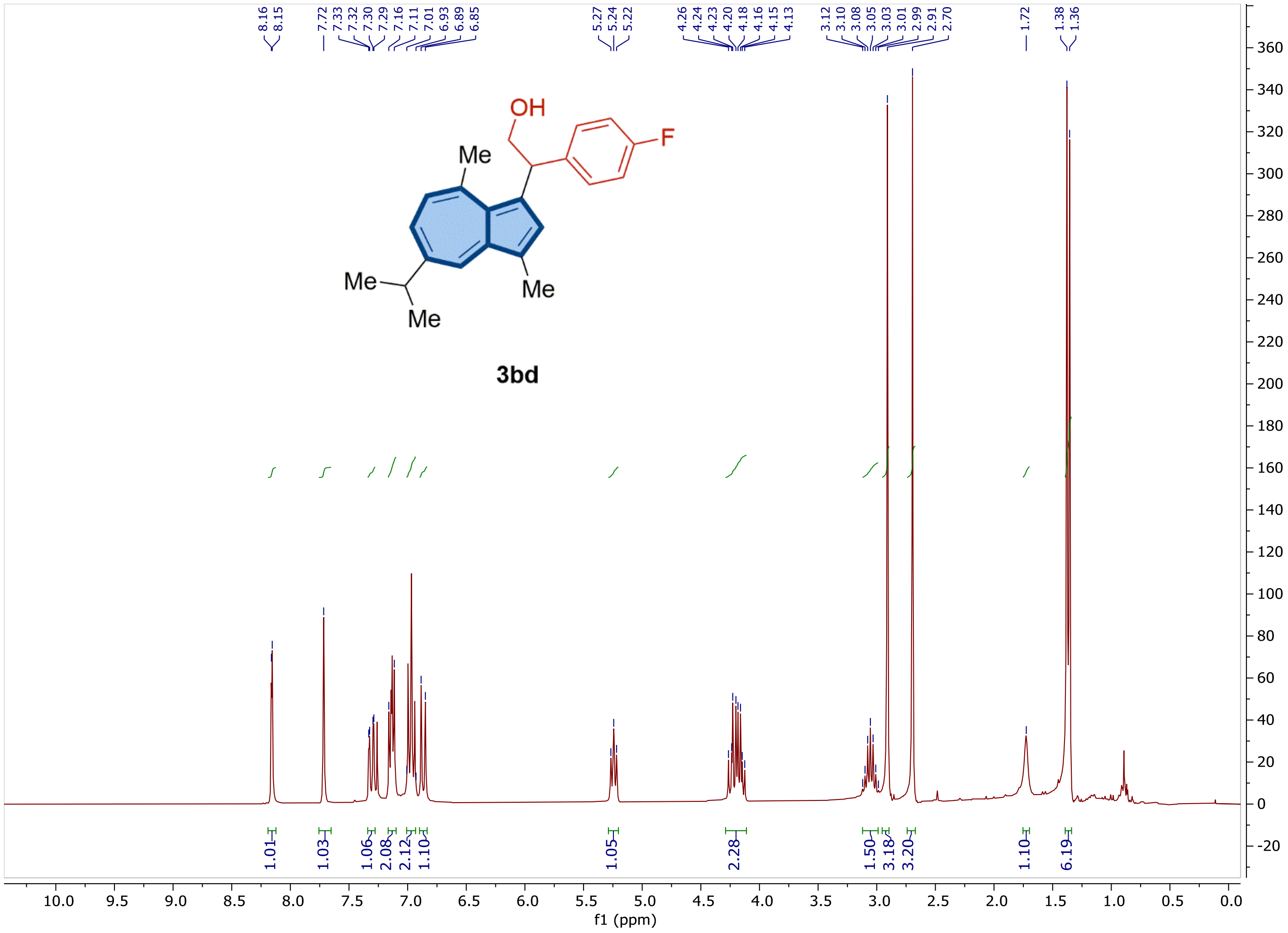

^{13}C NMR of compound 3bd (75 MHz, $CDCl_3$)

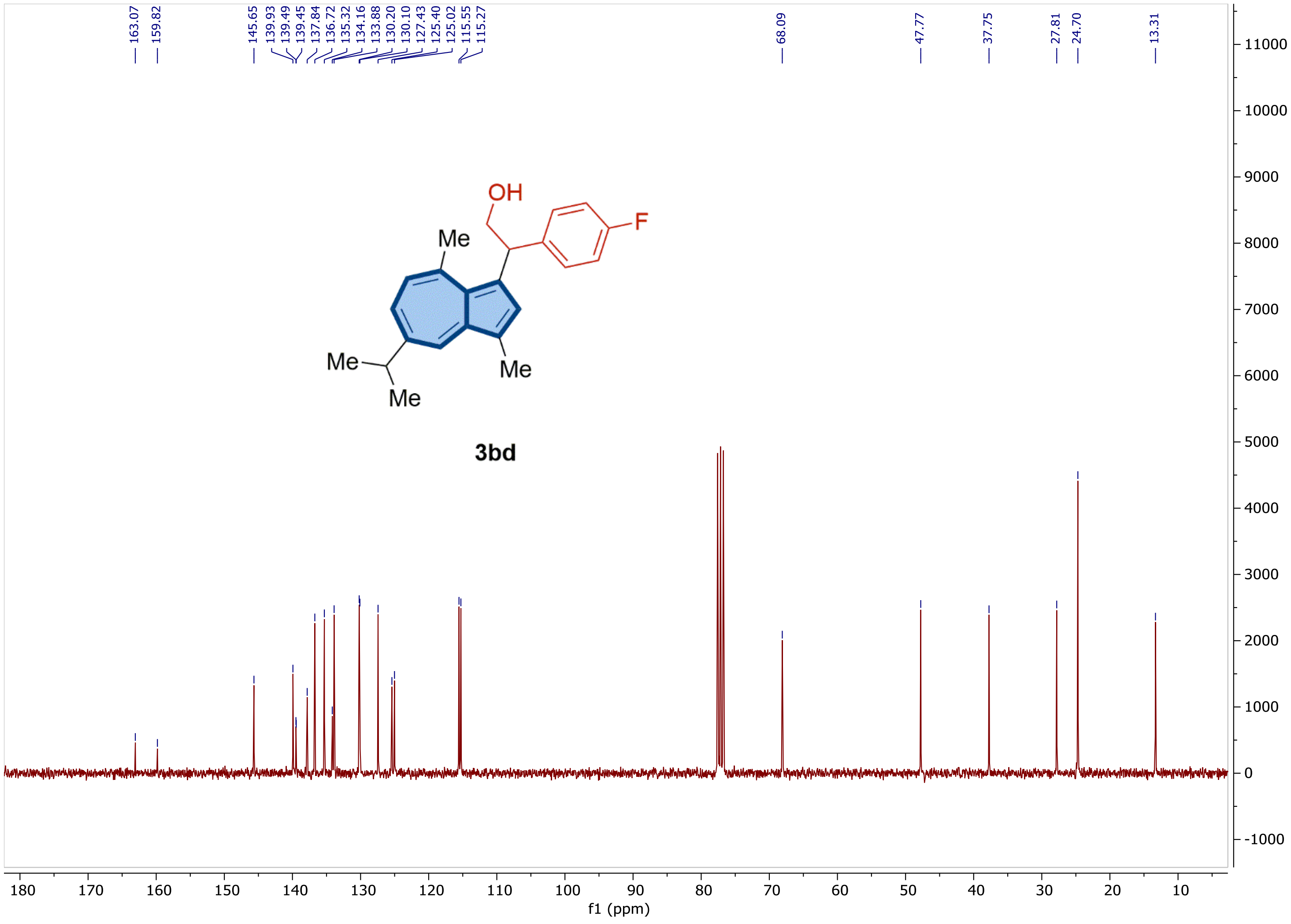

DEPT ^{13}C NMR of compound 3bd (75 MHz, $CDCl_3$)

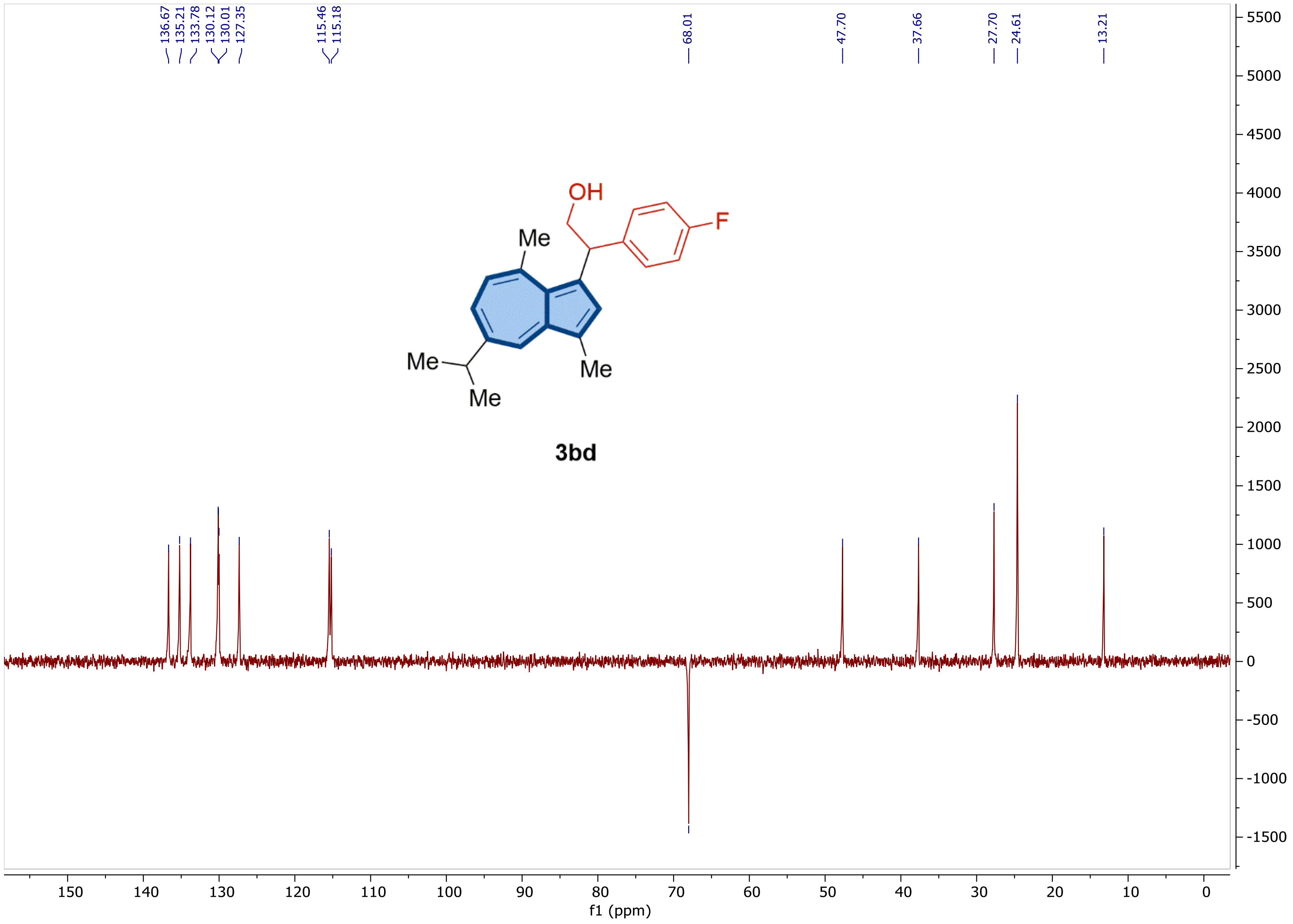

^{19}F NMR of compound 3bd (282 MHz, $CDCl_3$)

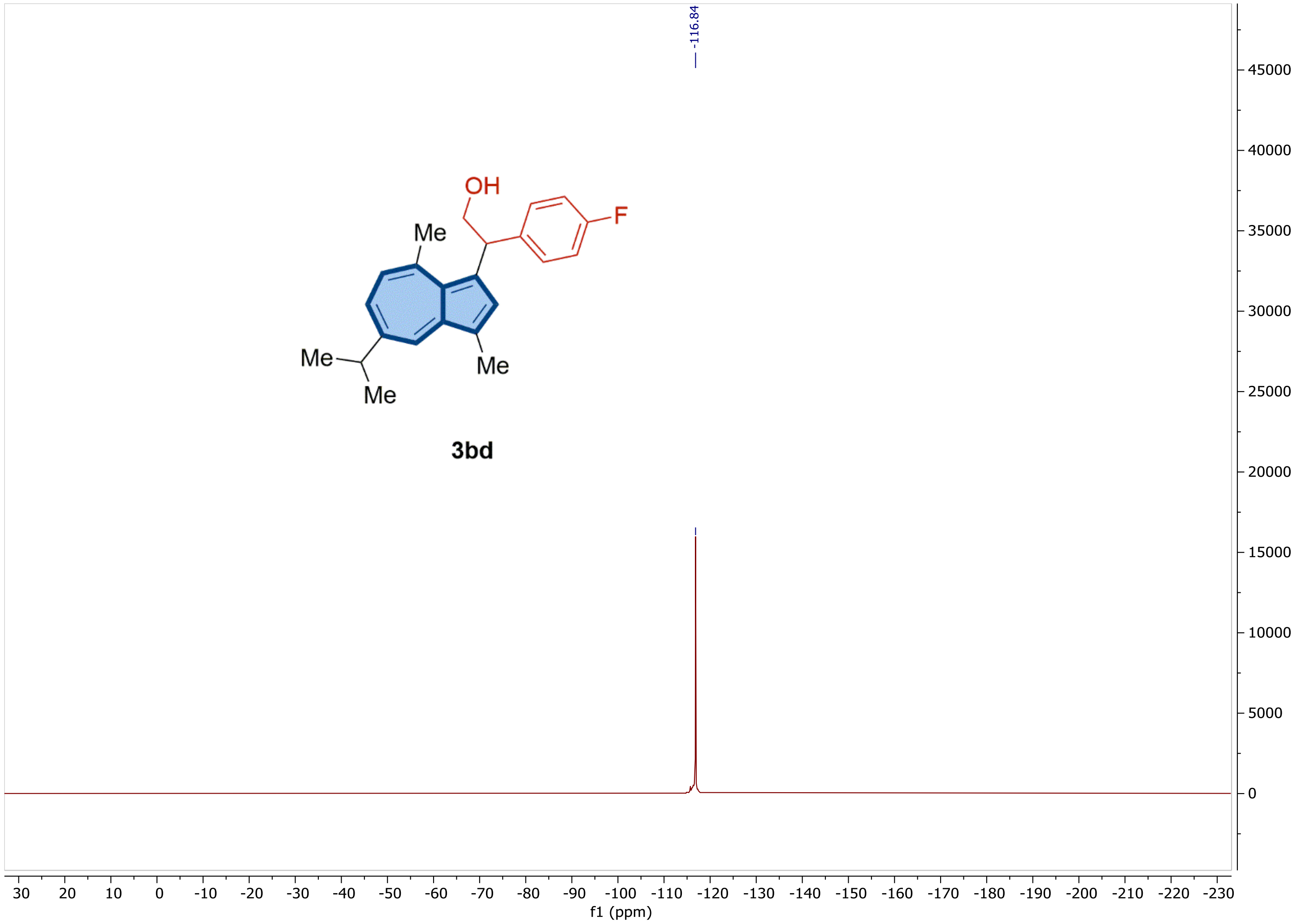

^{1}H NMR of compound 3be (300 MHz, $CDCl_3$)

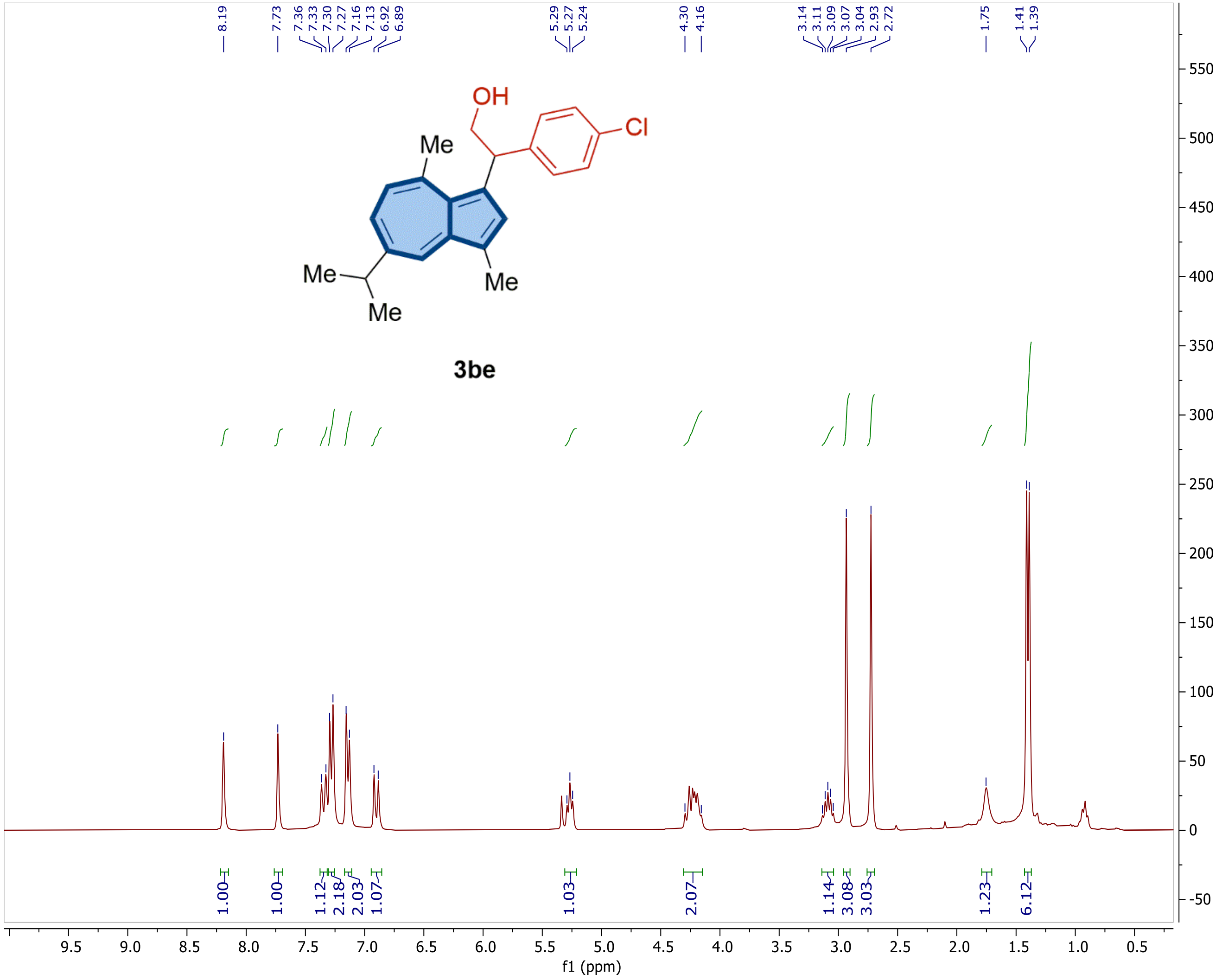

^{13}C NMR of compound 3be (75 MHz, $CDCl_3$)

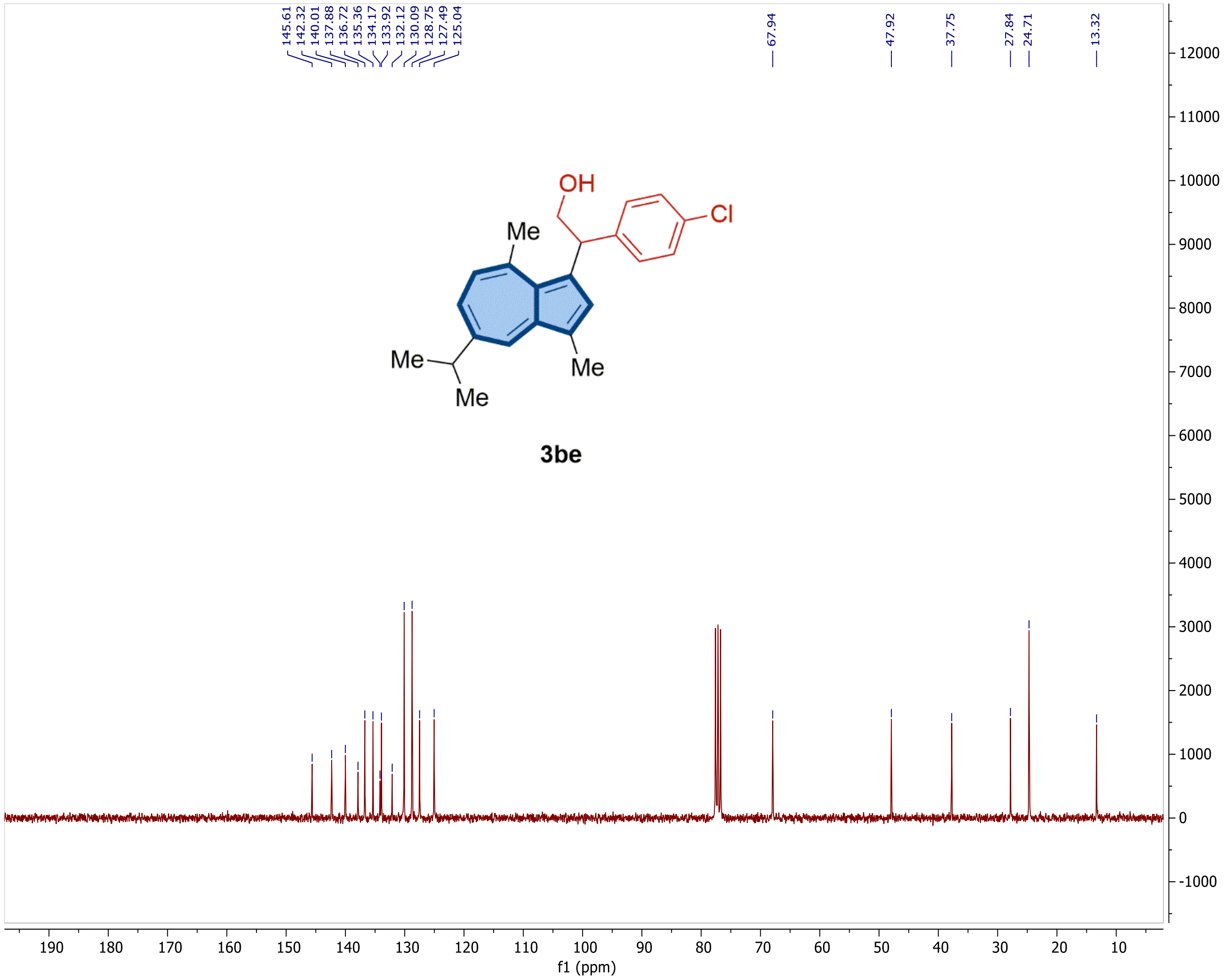

DEPT ^{13}C NMR of compound 3be (75 MHz, $CDCl_3$)

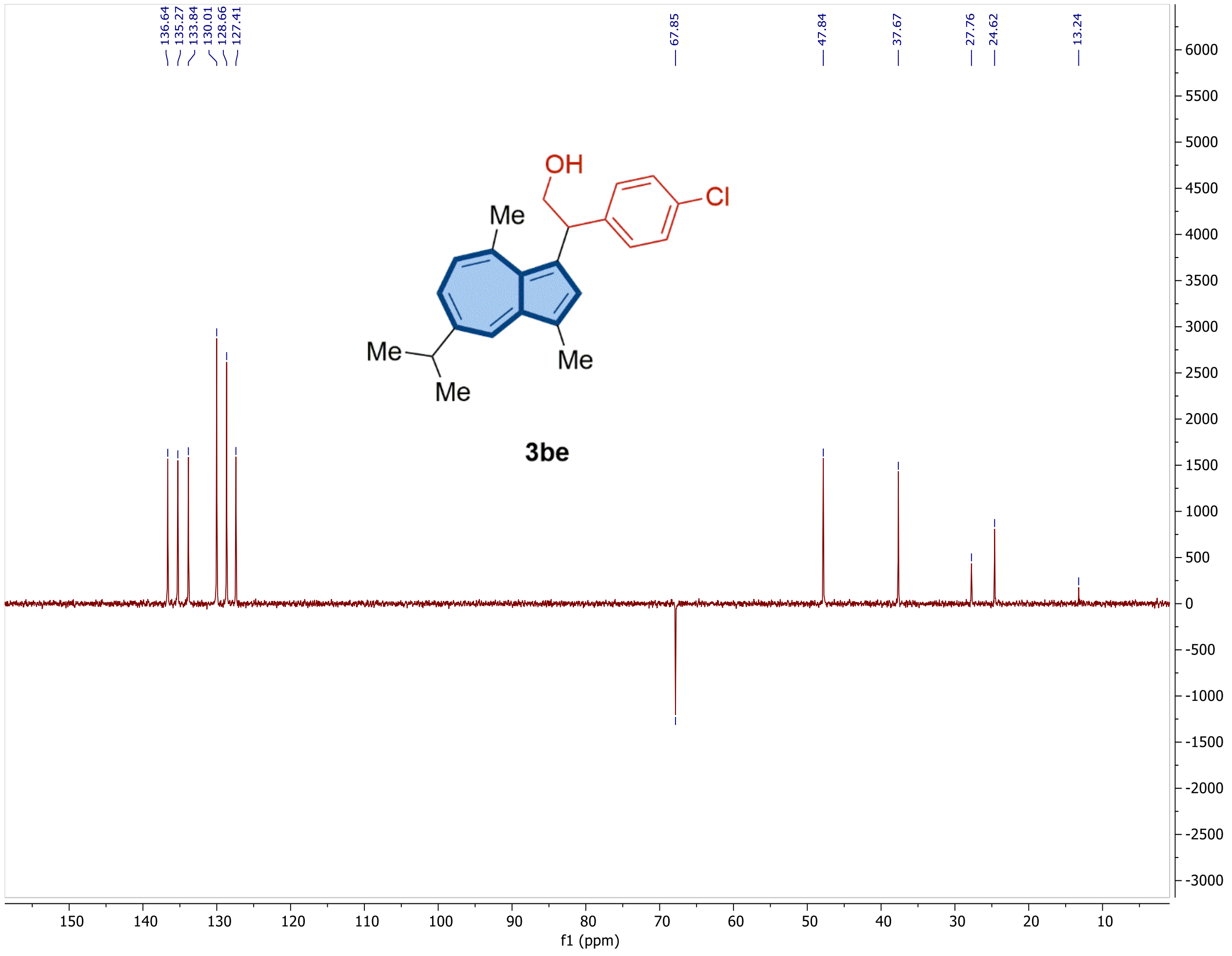

^{1}H NMR of compound 3bf (300 MHz, $CDCl_3$)

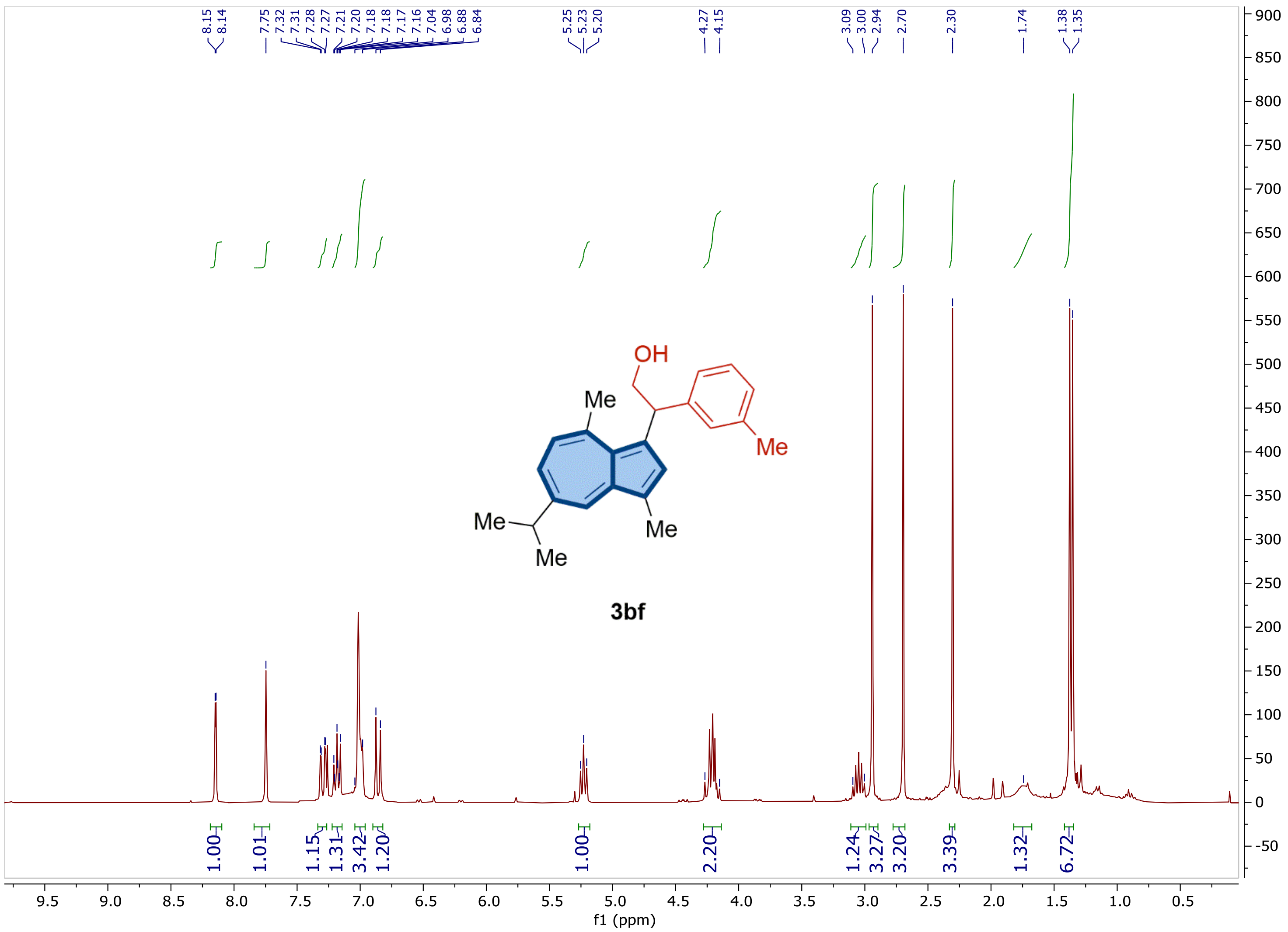

^{13}C NMR of compound 3bf (75 MHz, $CDCl_3$)

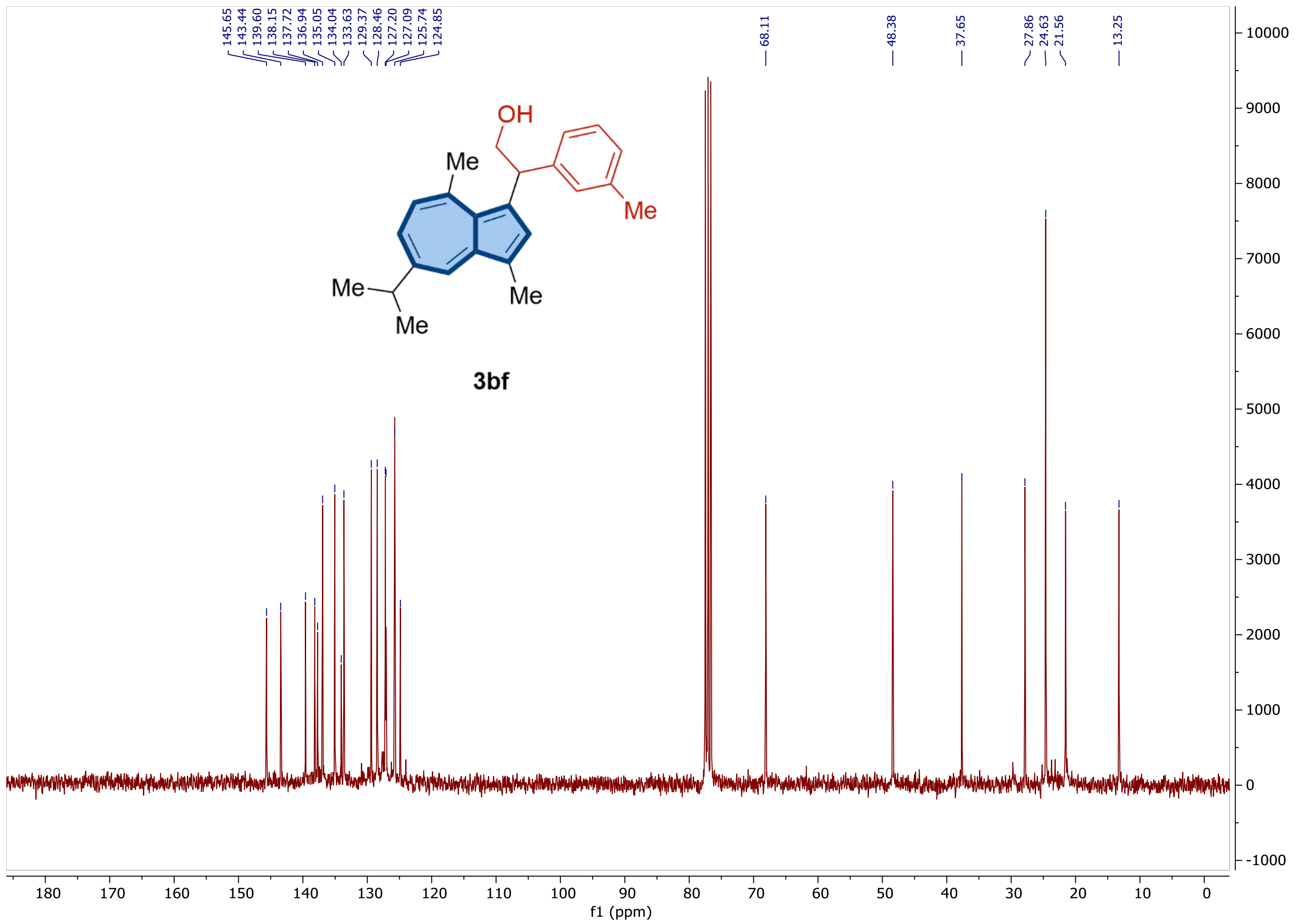

DEPT ^{13}C NMR of compound 3bf (75 MHz, $CDCl_3$)

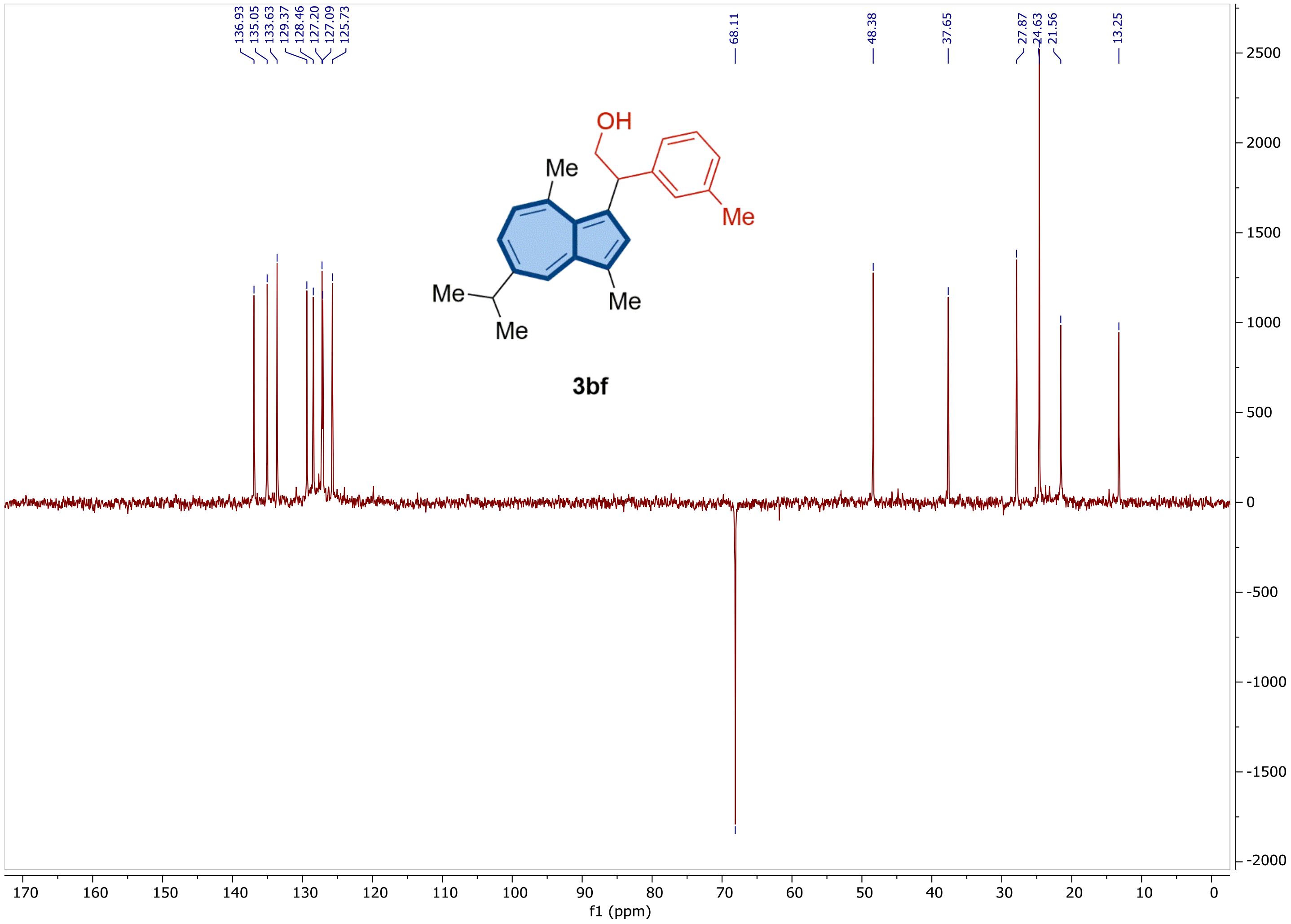

^{1}H NMR of compound 3bg (300 MHz, $CDCl_3$)

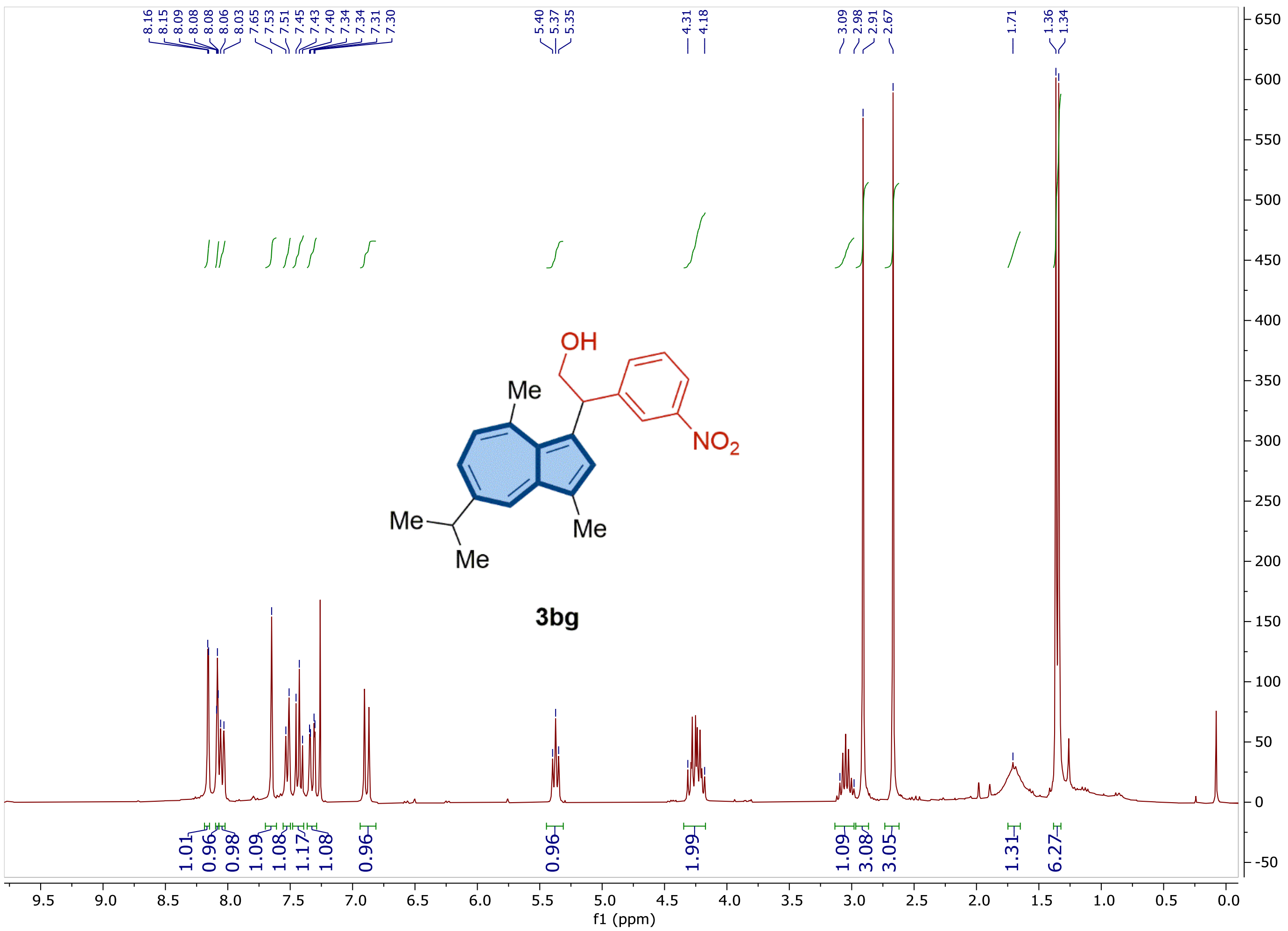

^{13}C NMR of compound 3bg (75 MHz, $CDCl_3$)

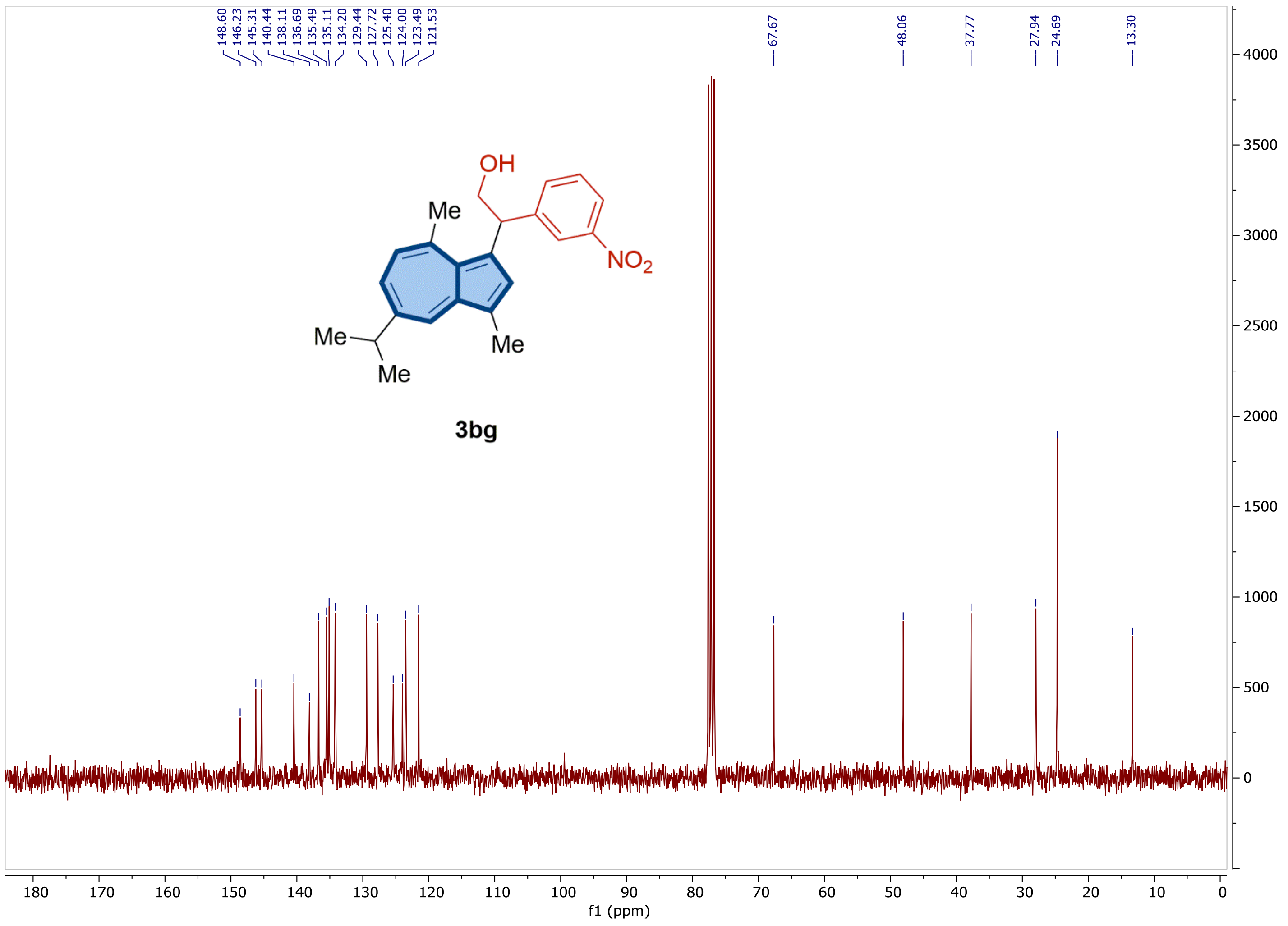

DEPT ^{13}C NMR of compound 3bg (75 MHz, $CDCl_3$)

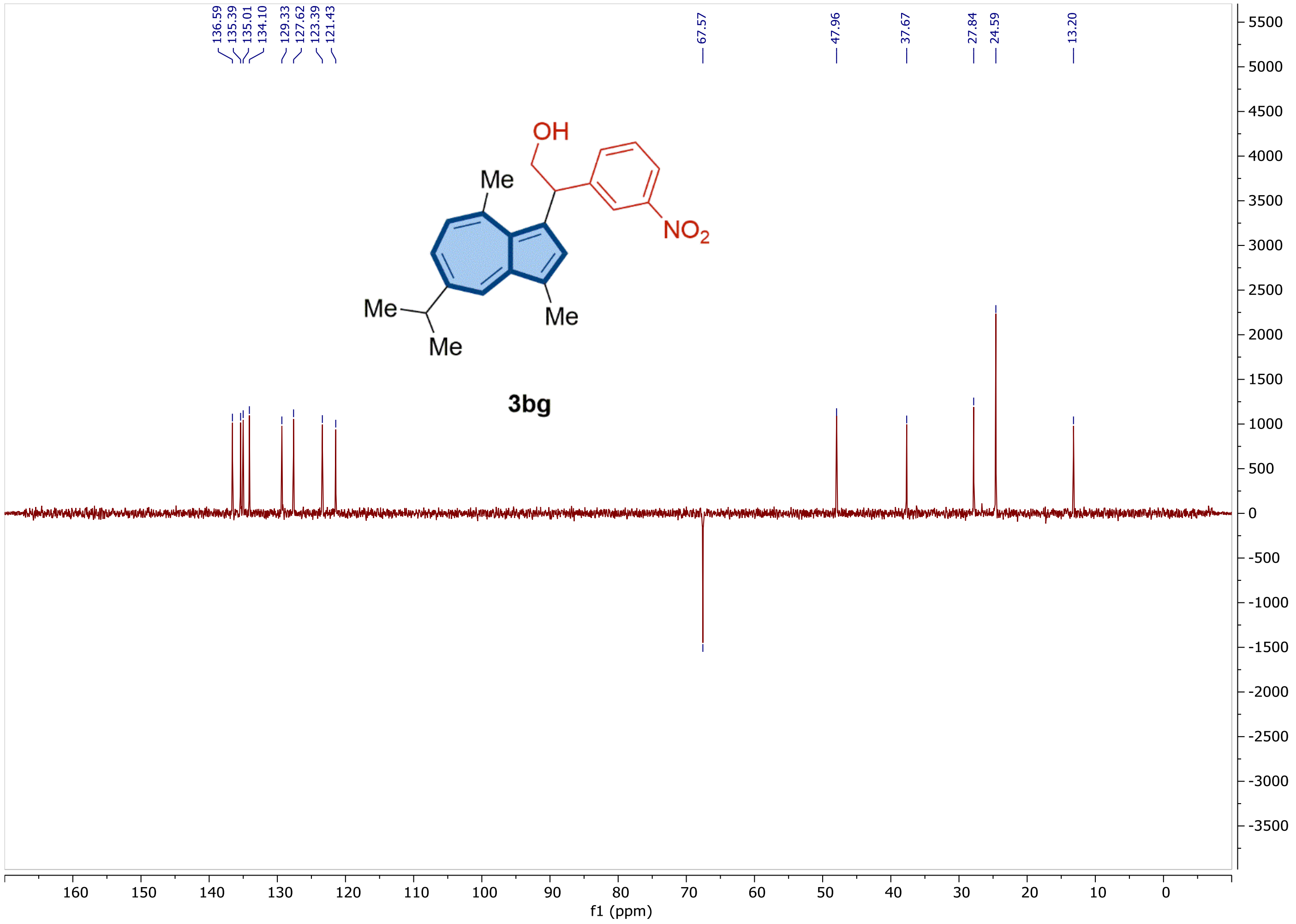

^{1}H NMR of compound 3bj (300 MHz, $CDCl_3$)

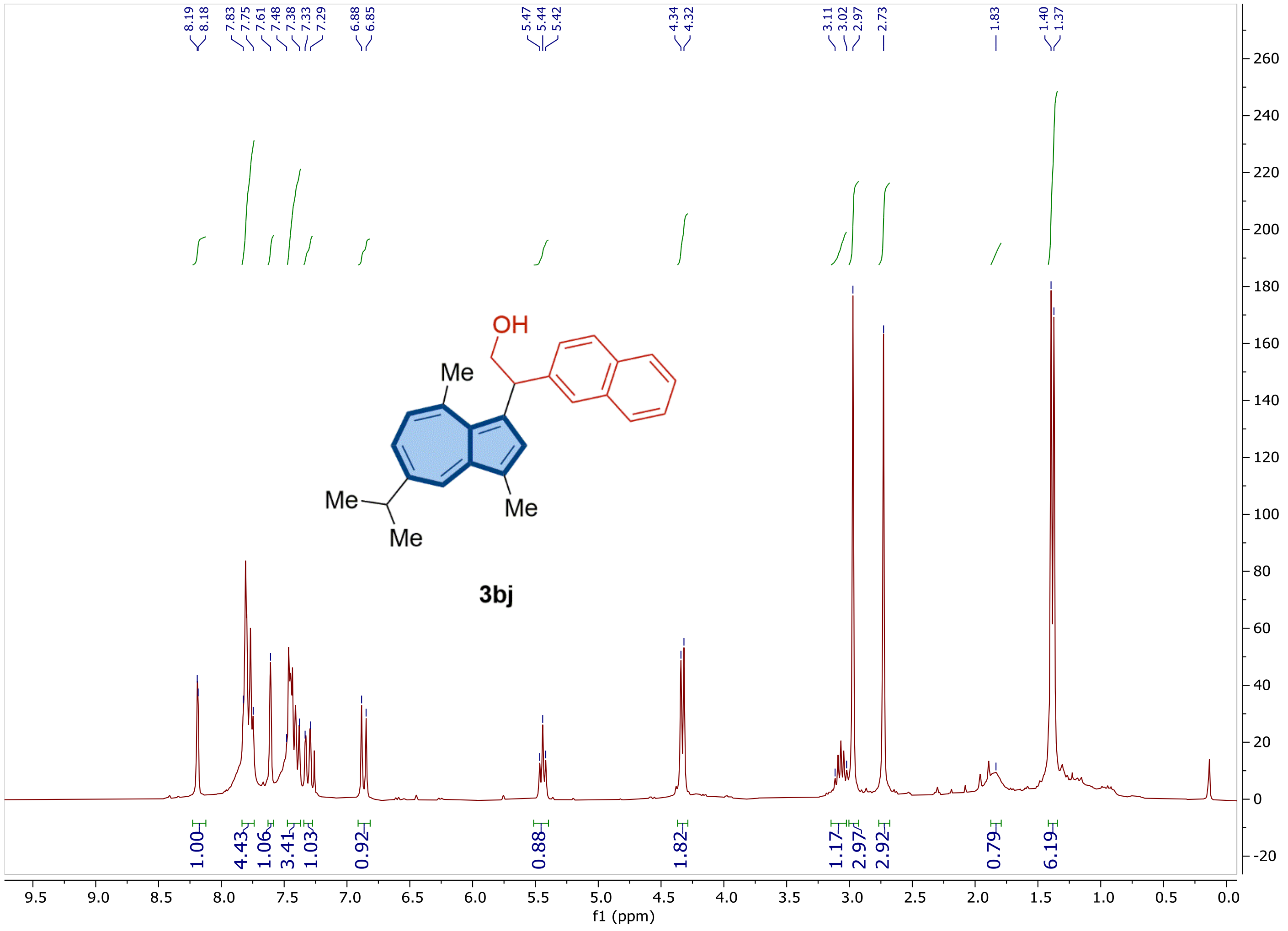

^{13}C NMR of compound 3bj (75 MHz, $CDCl_3$)

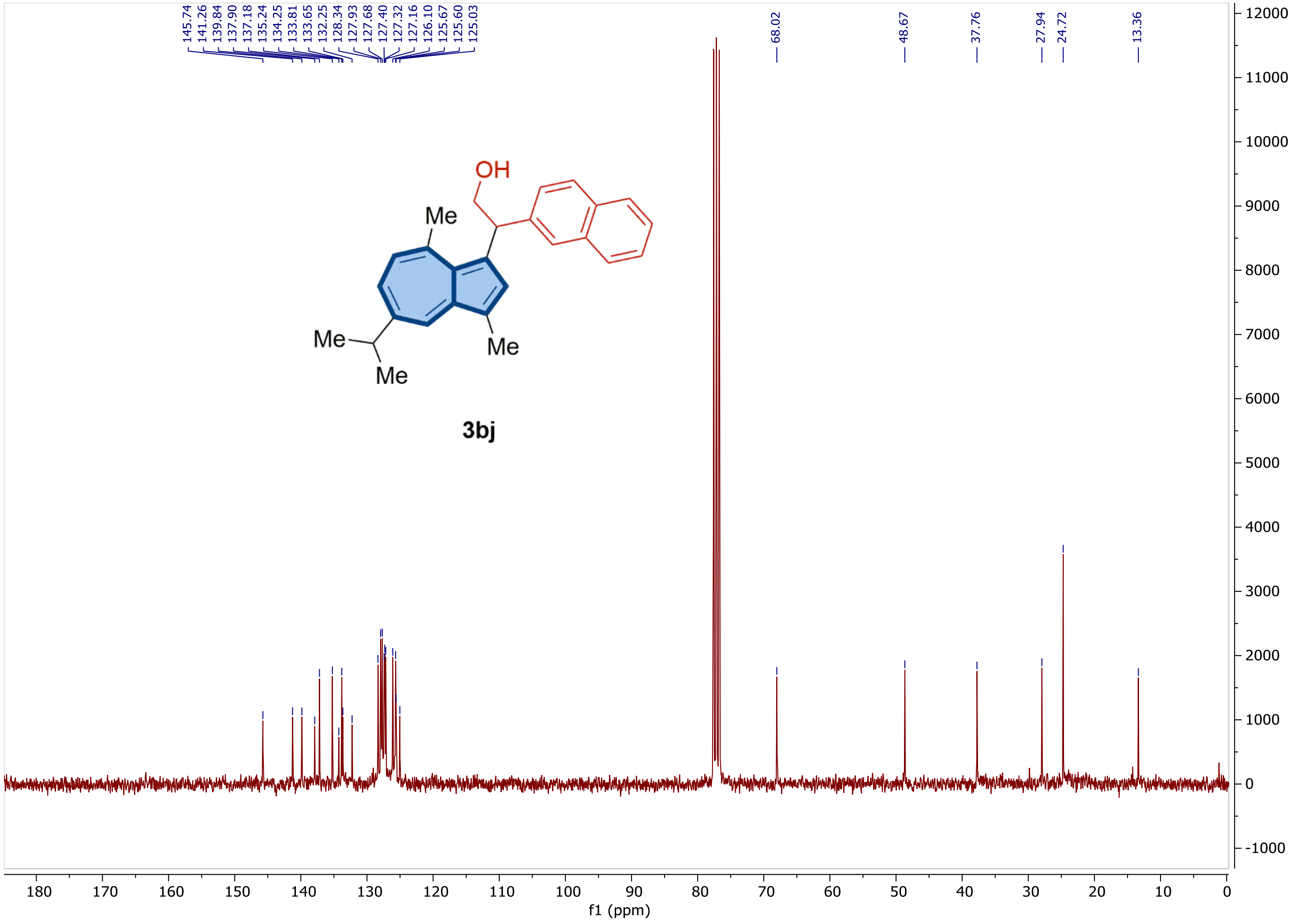

DEPT ^{13}C NMR of compound 3bj (75 MHz, $CDCl_3$)

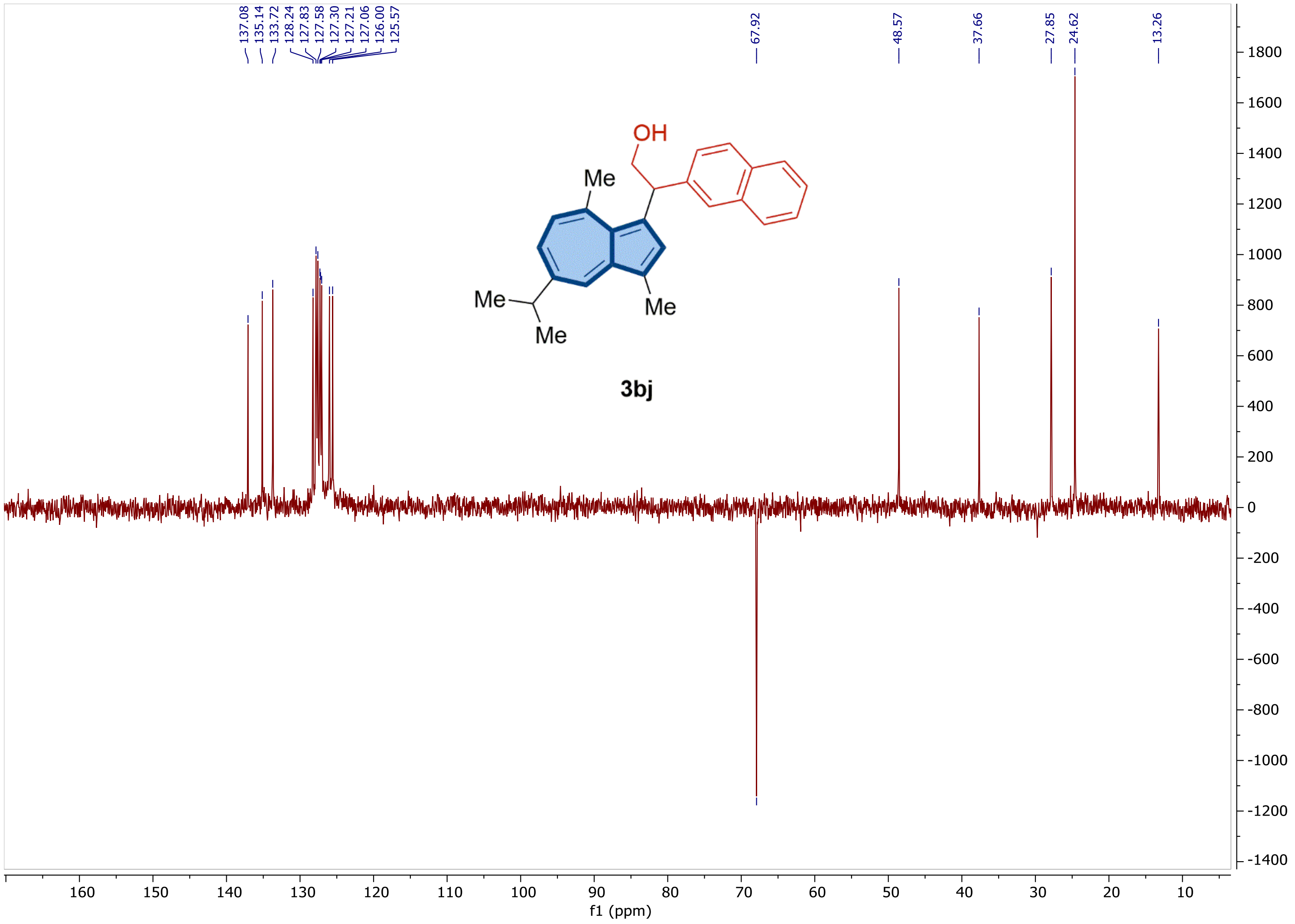

^{1}H NMR of compound 3ca (300 MHz, $CDCl_3$)

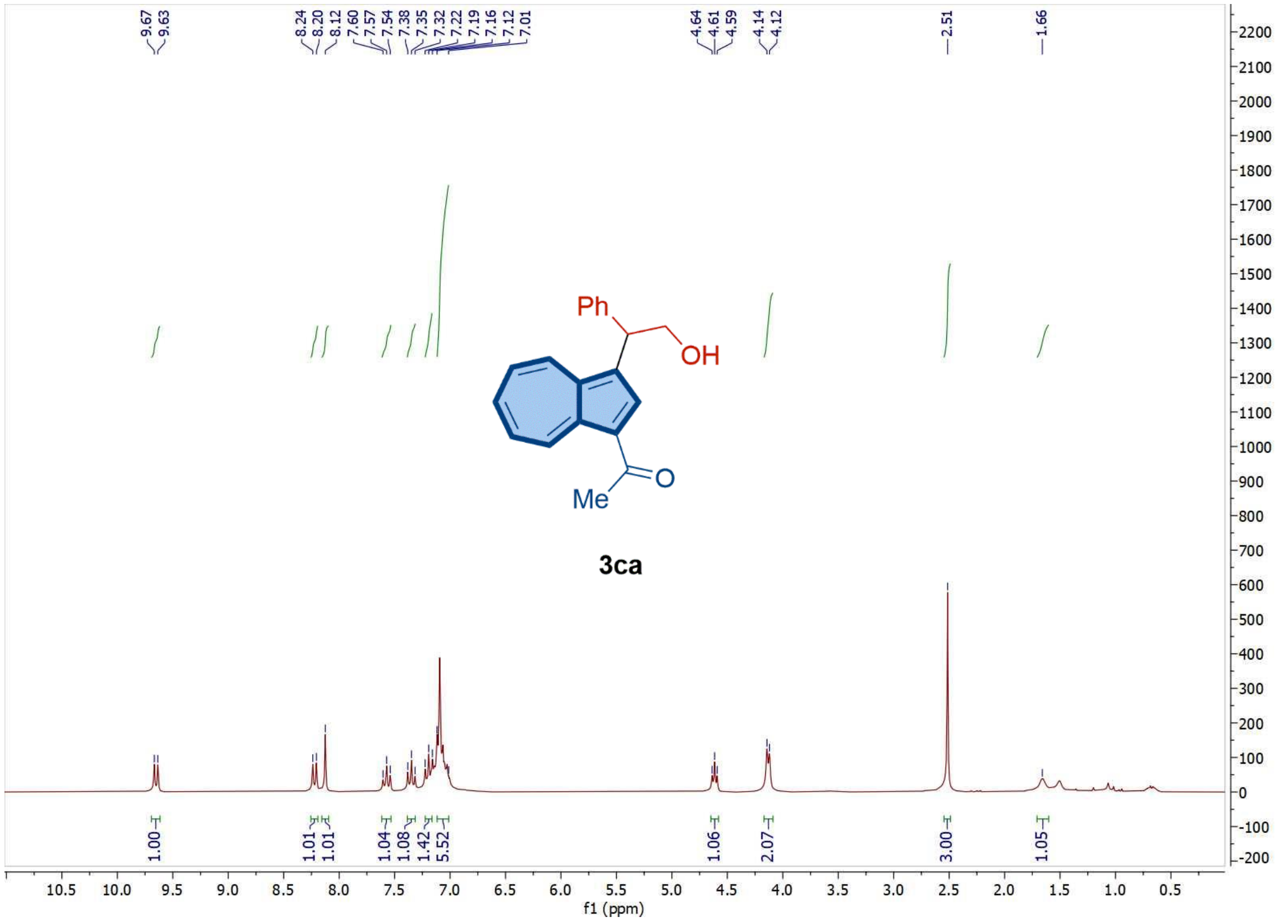

^{13}C NMR of compound 3ca (75 MHz, $CDCl_3$)

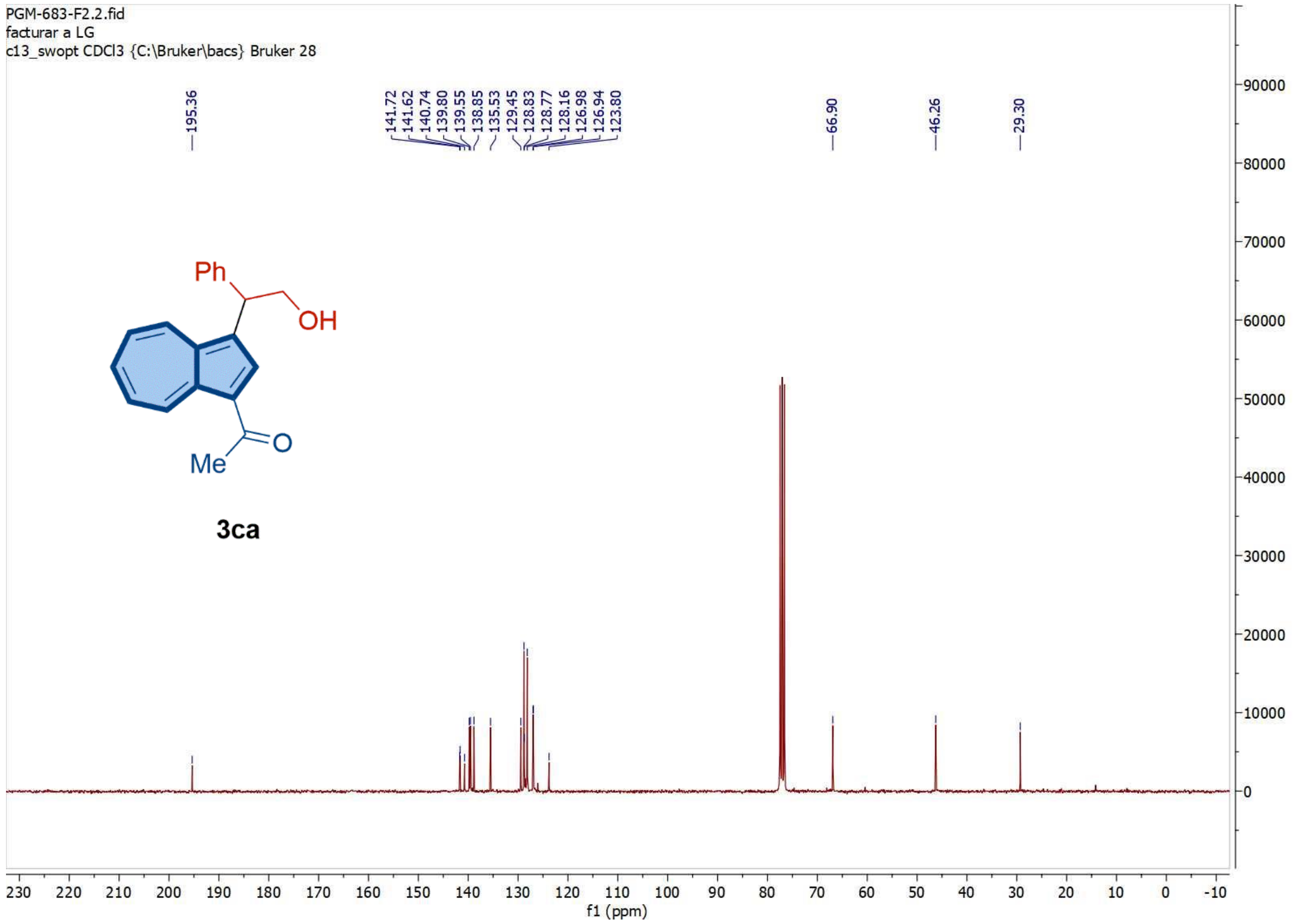

DEPT ^{13}C NMR of compound 3ca (75 MHz, $CDCl_3$)

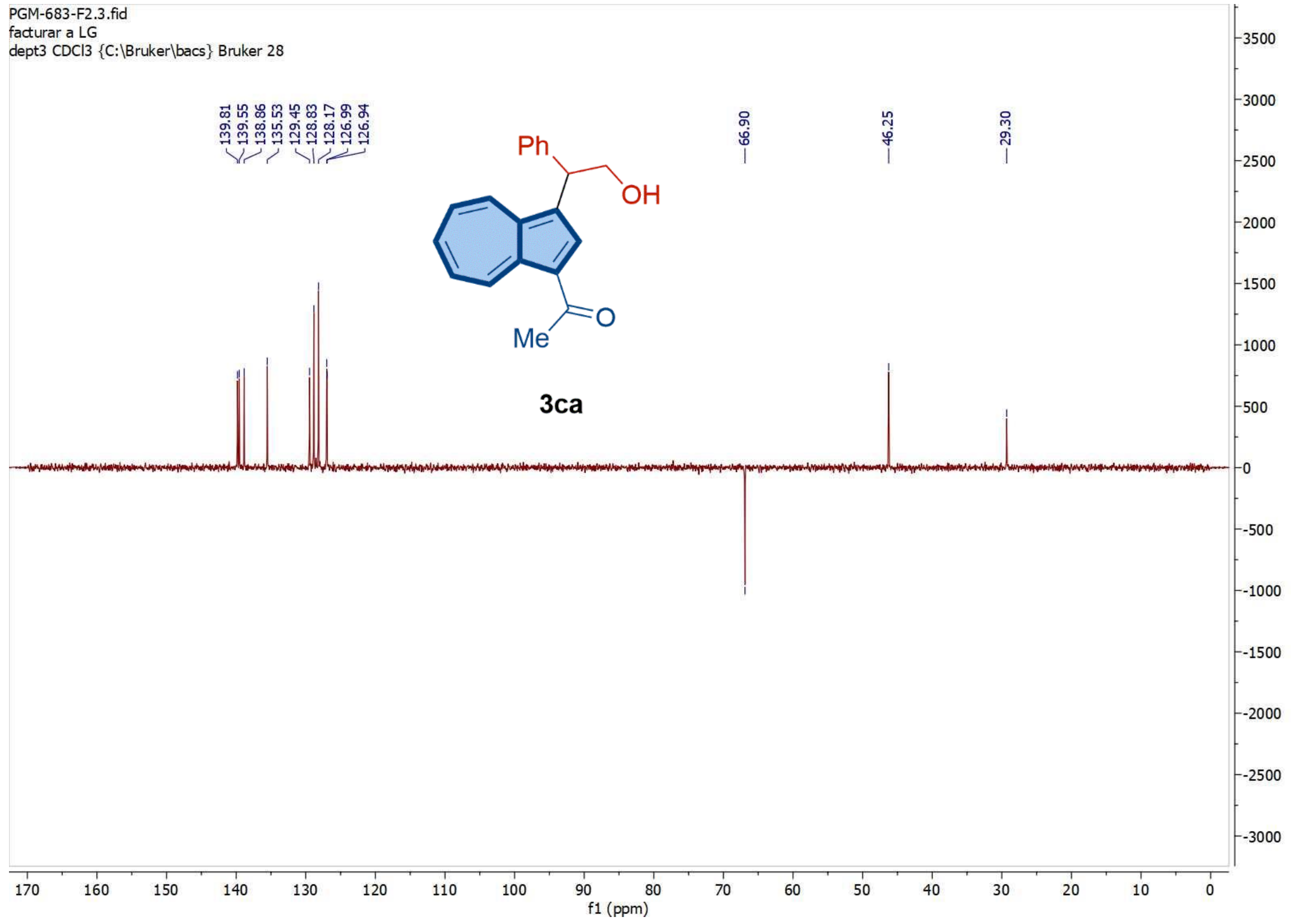

^{1}H NMR of compound 3aa’ (300 MHz, $CDCl_3$)

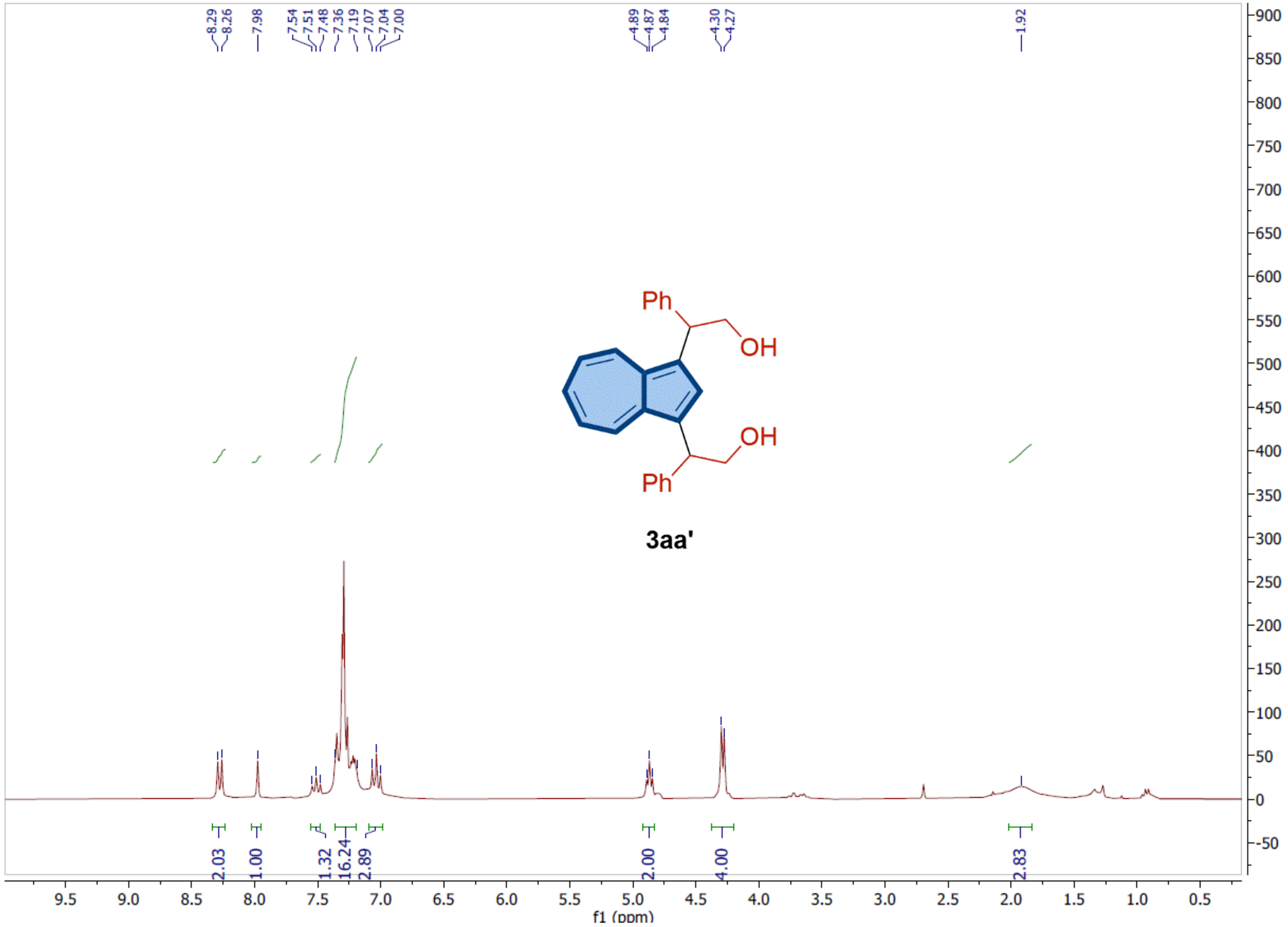

^{13}C NMR of compound 3aa' (75 MHz, $CDCl_3$)

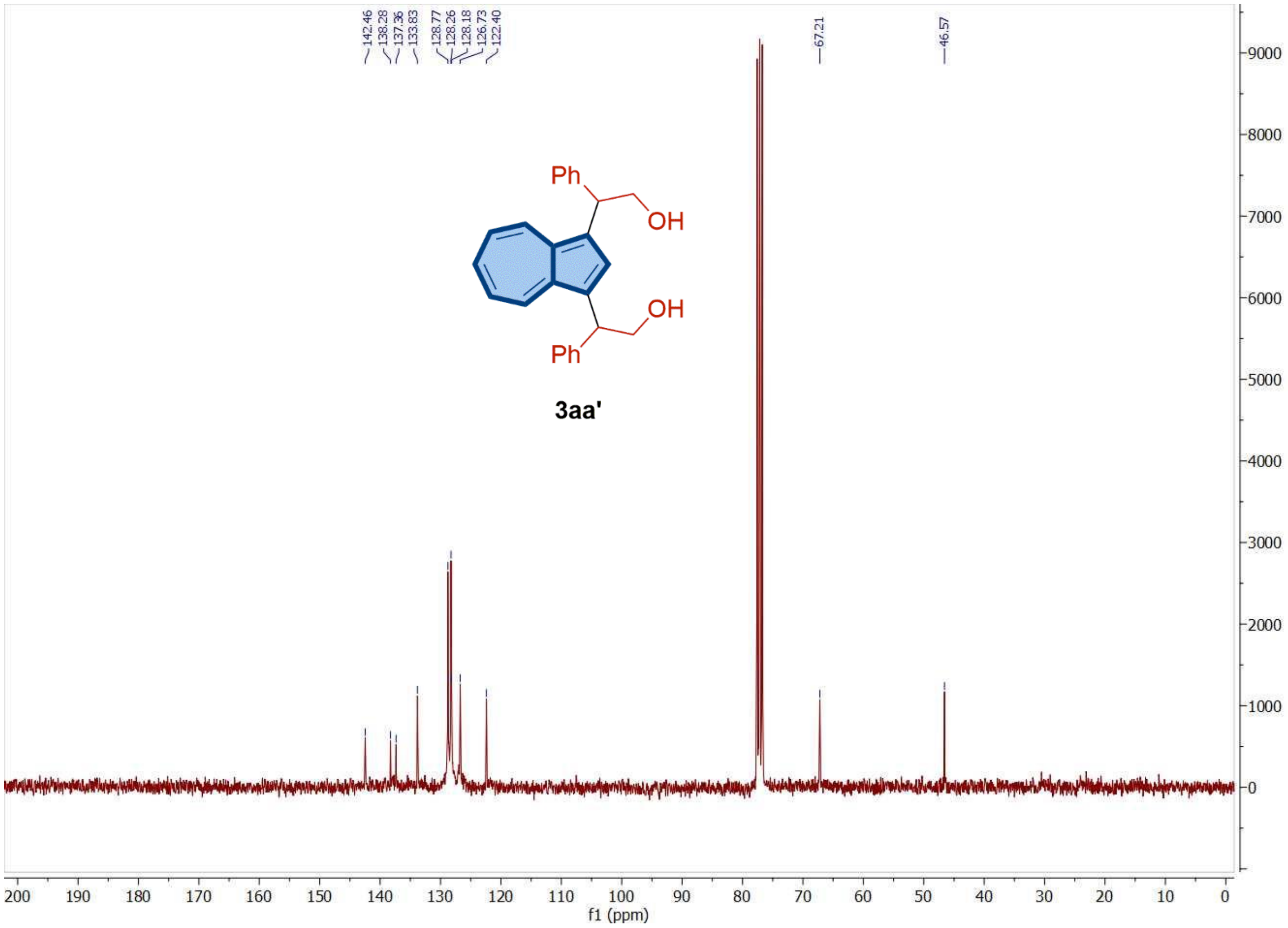

^{1}H NMR of compound 5aa (300 MHz, $CDCl_3$)

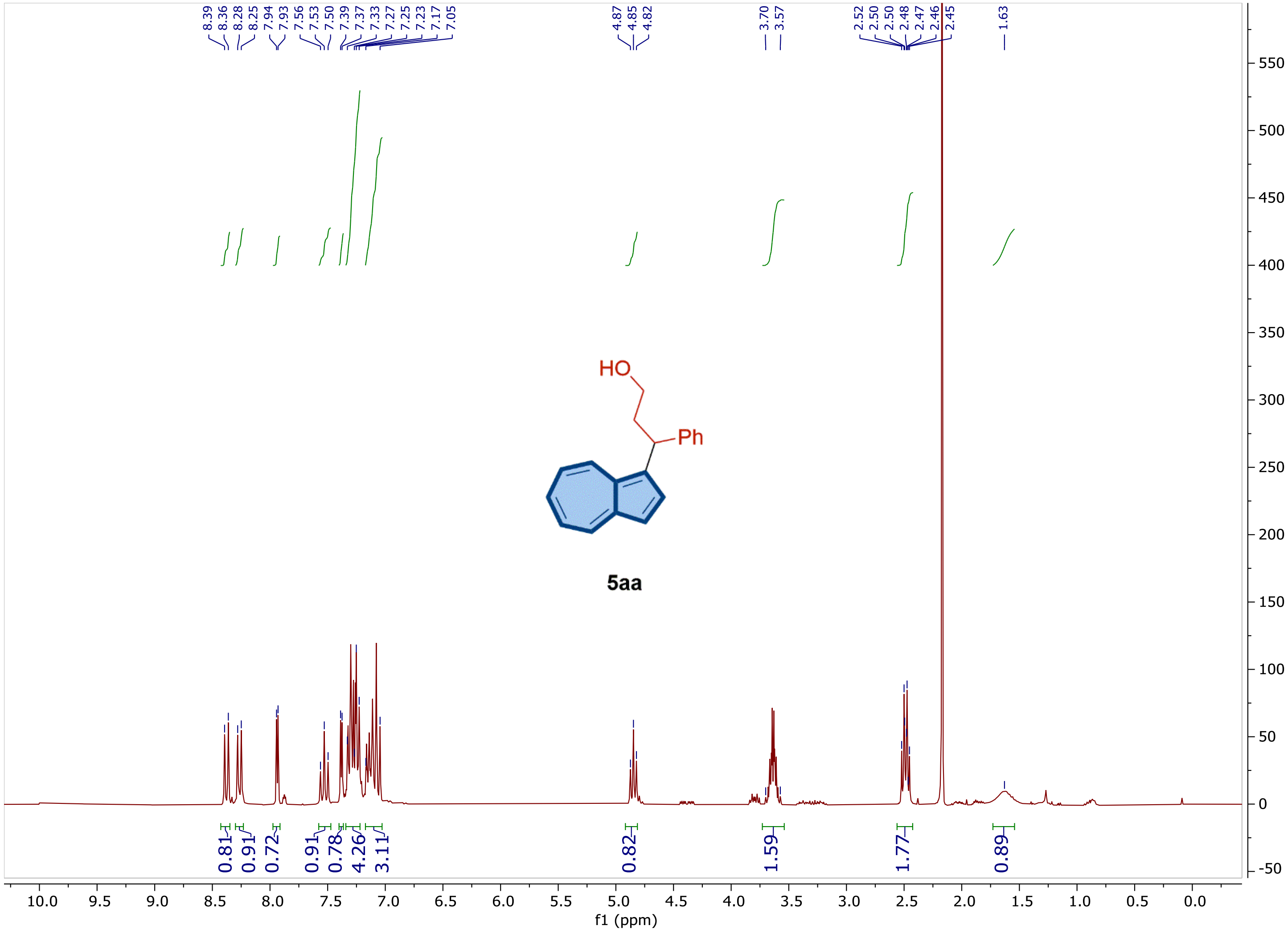

^{13}C NMR of compound 5aa (75 MHz, $CDCl_3$)

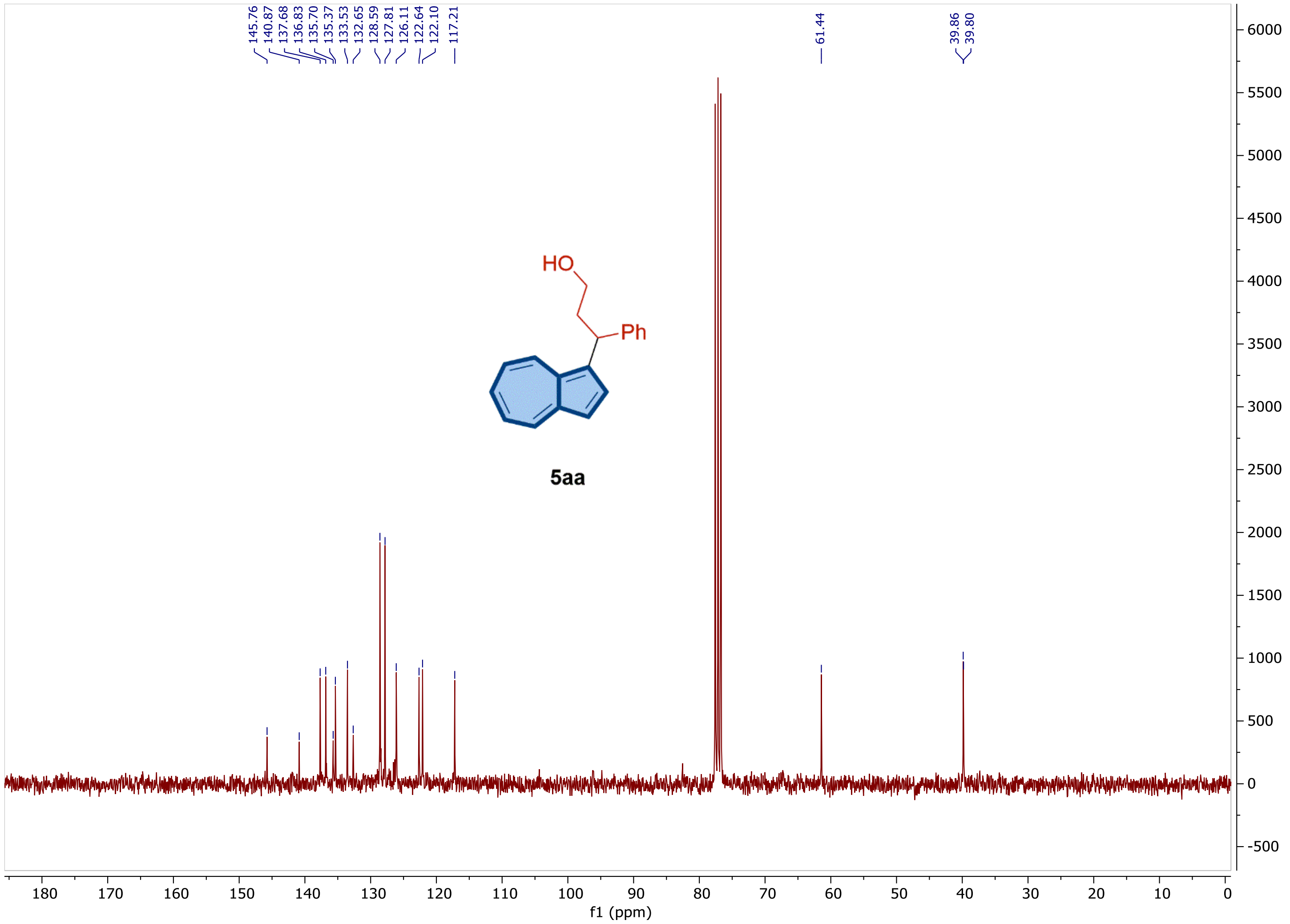

DEPT ^{13}C NMR of compound 5aa (75 MHz, $CDCl_3$)

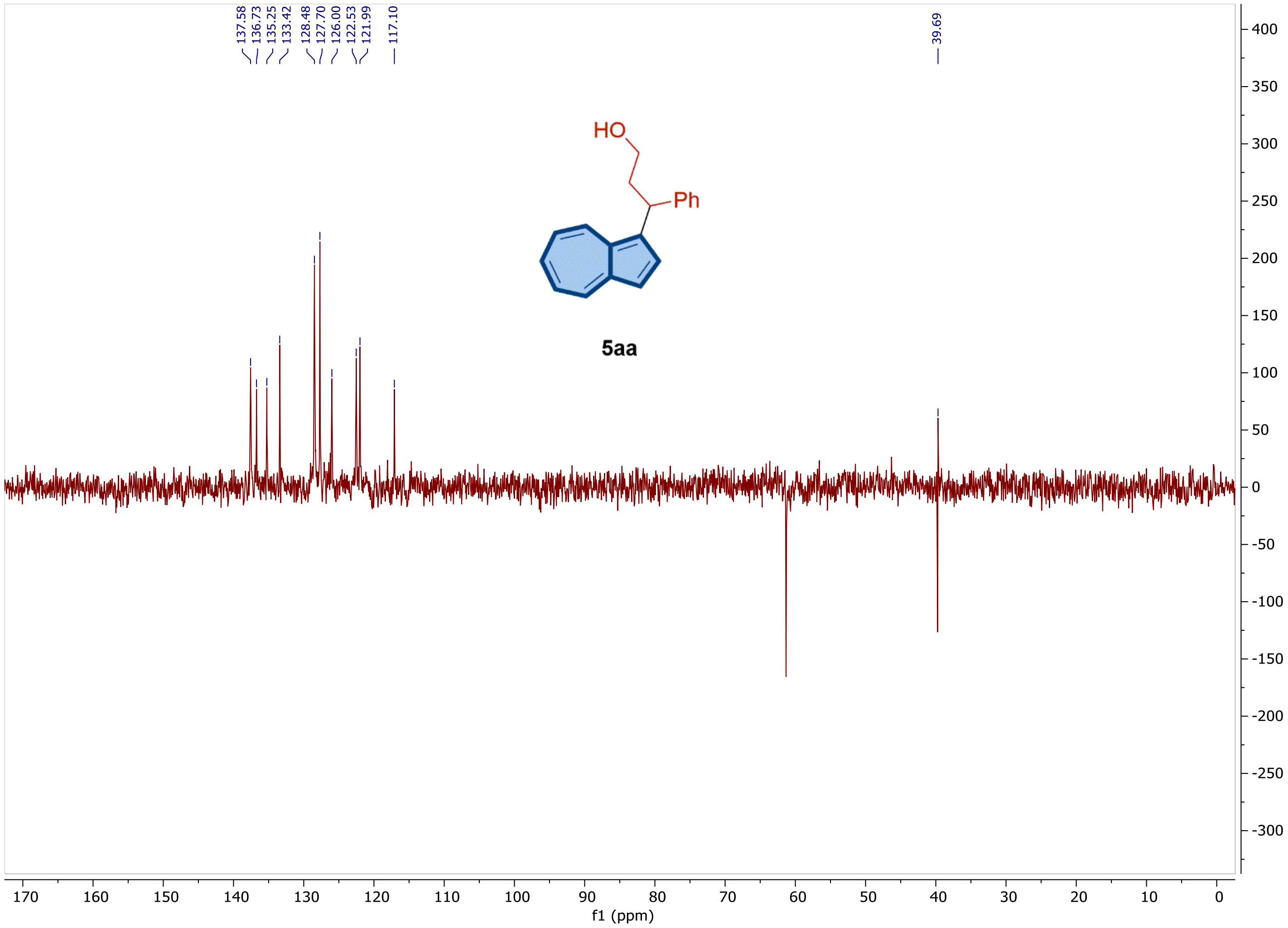

^{1}H NMR of compound 5ba (300 MHz, $CDCl_3$)

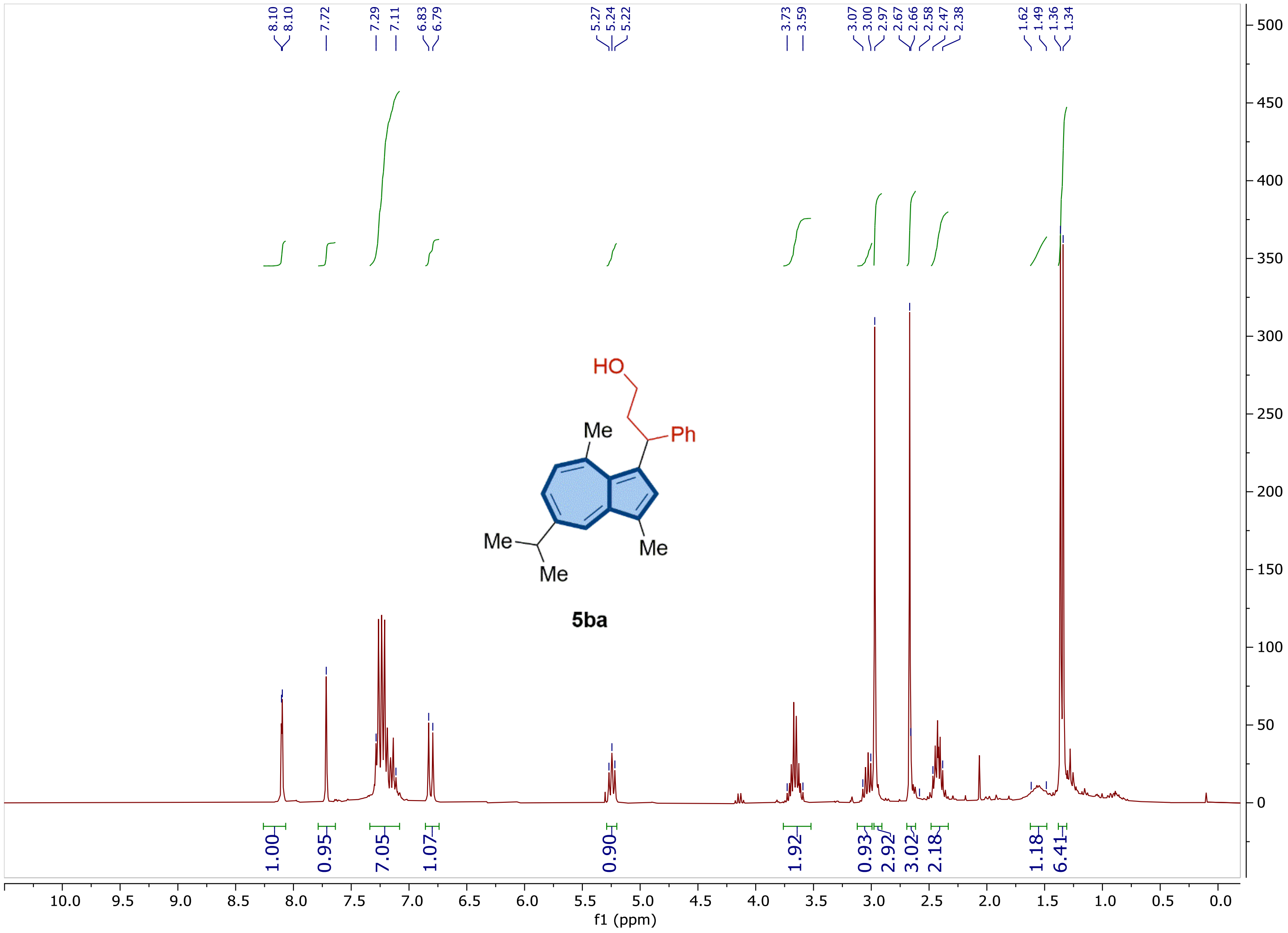

^{13}C NMR of compound 5ba (75 MHz, $CDCl_3$)

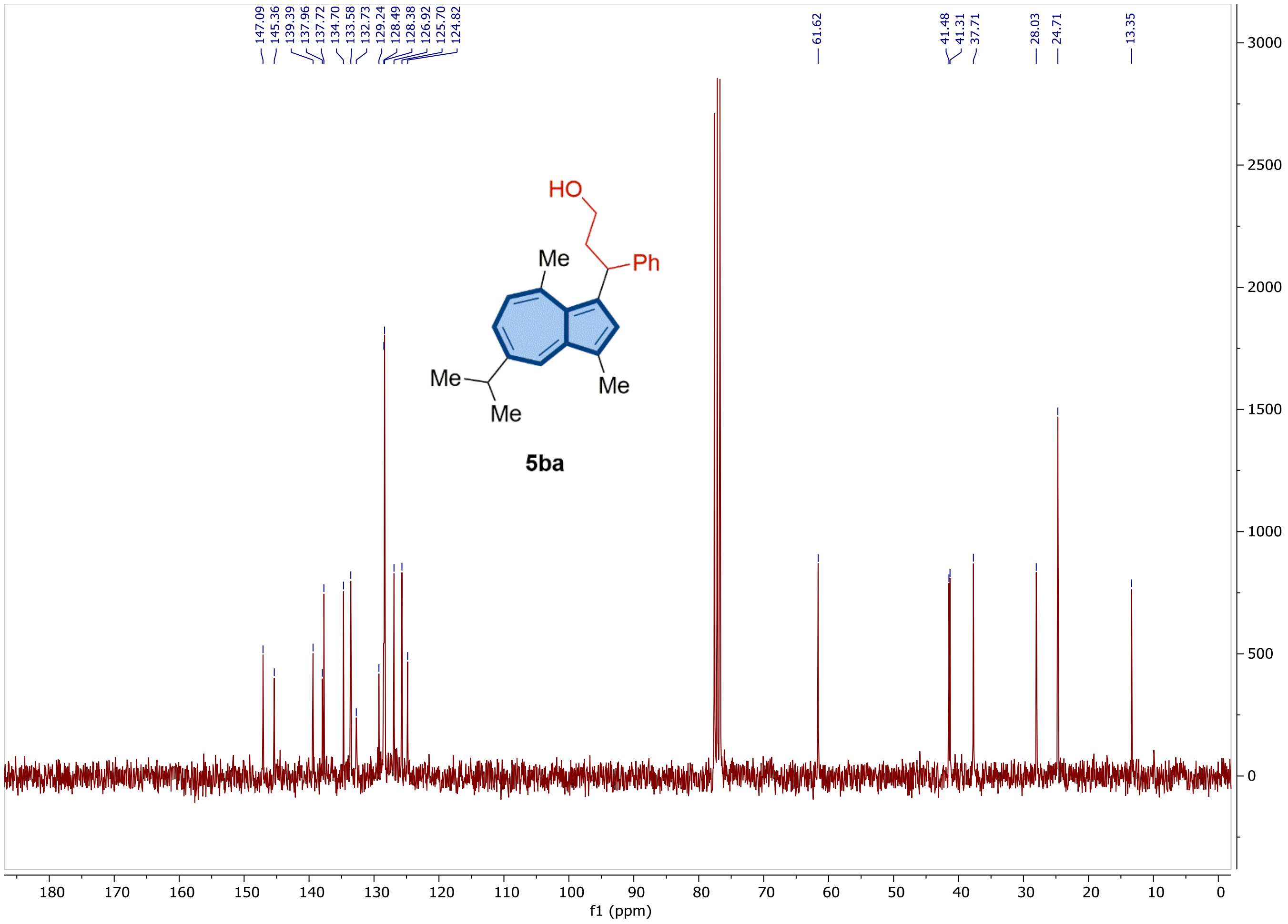

DEPT ^{13}C NMR of compound 5ba (75 MHz, $CDCl_3$)

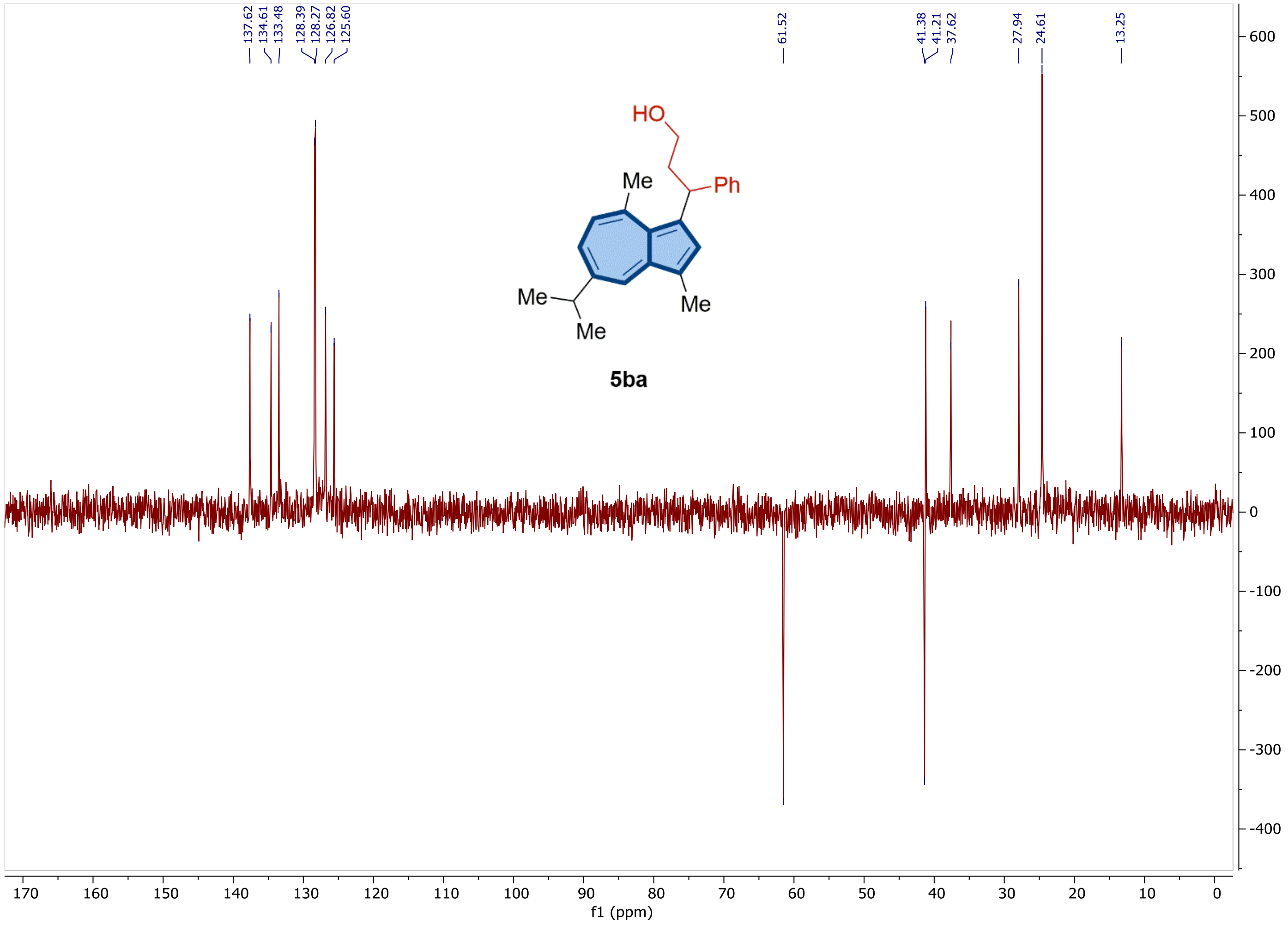

^{1}H NMR of compound 6 (300 MHz, $CDCl_3$)

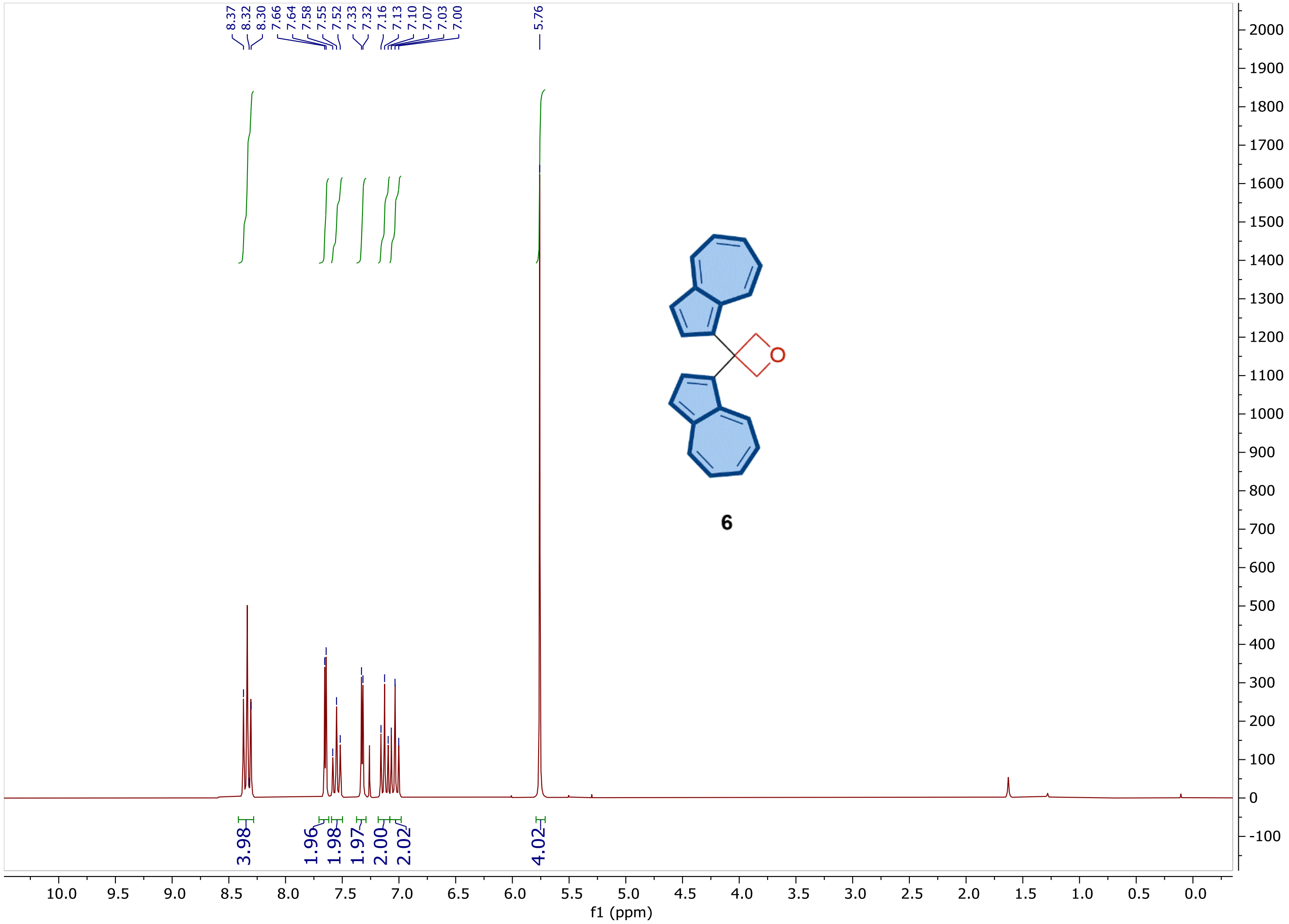

^{13}C NMR of compound 6 (75 MHz, $CDCl_3$)

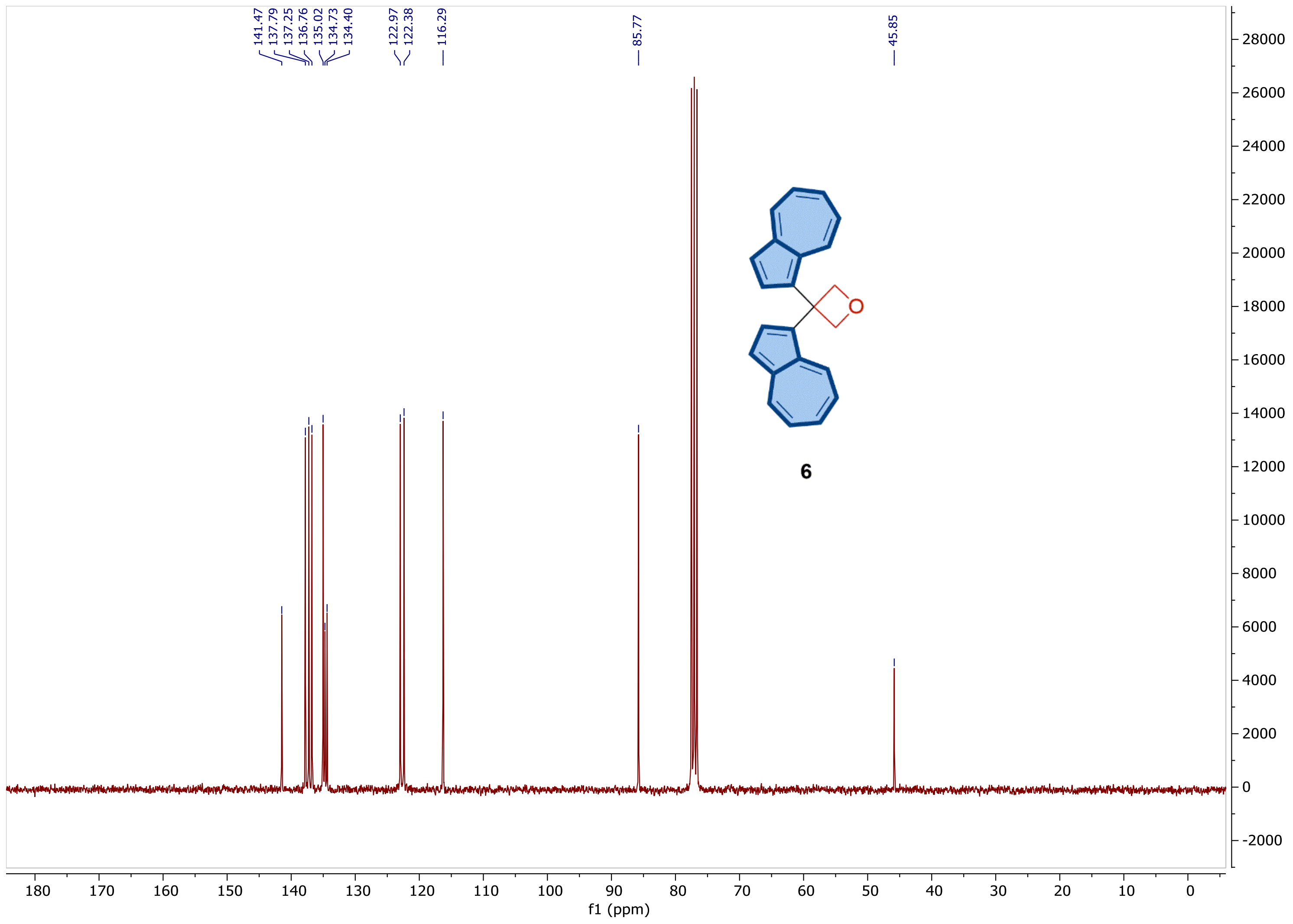

DEPT ^{13}C NMR of compound 6 (75 MHz, $CDCl_3$)

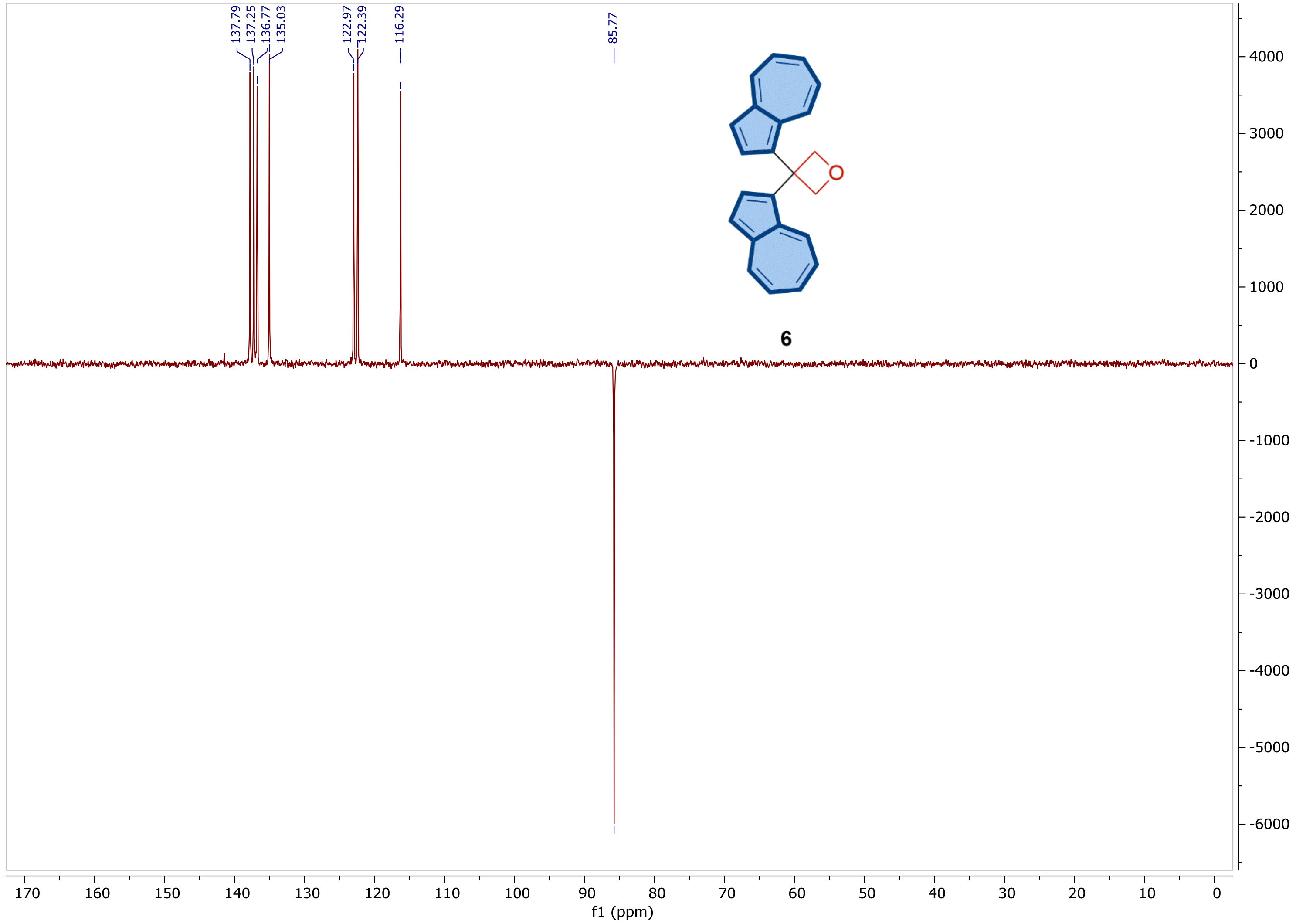

Supplement: Supplementary file 1 [file ol5c02648_si_001.pdf]
